# Supplementary material for: Isolation and molecular characterization of West Nile Virus with evidence of vertical transmission in the Coastal region, Kenya
Source: Microbiol Spectr. 2026 Aug 25;14(10):e04205-25. doi: 10.1128/spectrum.04205-25 (PMC13688926; doi:10.1128/spectrum.04205-25)
Supplement: Supplementary file 1 [file reviewer-comments.pdf]

ASM | Microbiology Spectrum

Detection and Characterization of West Nile Virus with evidence of Transovarial Transmission in Coastal Kenya

Tabitha Wanjiru, Solomon Langat, Hellen Koka, Gladys Kerich, Janet Ambale, Santos Yalwala, Jaree Johnson, Eric Garges, Gerald Kellar, Robert Haynes, John Eads, and Fredrick Eyase

Corresponding Author(s): Tabitha Wanjiru, Kenya Medical Research Institute

Review Timeline:

Submission Date:	January 6, 2026
Editorial Decision:	February 25, 2026
Revision Received:	April 15, 2026
Editorial Decision:	June 15, 2026
Revision Received:	July 16, 2026
Accepted:	July 23, 2026

Editor: My Phan

Reviewer(s): Disclosure of reviewer identity is with reference to reviewer comments included in decision letter(s). The following individuals involved in review of your submission have agreed to reveal their identity: Luciano M. Tantely (Reviewer #2)

Transaction Report:

(Note: With the exception of the correction of typographical or spelling errors that could be a source of ambiguity, letters and reports are not edited. The original formatting of letters and referee reports may not be reflected in this compilation.)

DOI: https://doi.org/10.1128/spectrum.04205-25

Re: Spectrum04205-25 (**Detection and Characterization of West Nile Virus with evidence of Transovarial Transmission in Coastal Kenya**)

Dear Dr. Tabitha Wanjiru:

Thank you for submitting your manuscript to Spectrum. Below you will find my editorial comments, instructions from our office, and the feedback from the reviewers.

Data Availability: As noted by the reviewers, several required datasets are currently inaccessible. This includes the WNV genome sequences, COI sequences used for species typing, and the corresponding raw sequence data for both. According to our Data Availability policy, all sequencing reads and assembled sequences must be deposited in a public repository and made publicly available before publication. Please make sure you address this data availability in your revision.

The reviewers have raised major concerns regarding the WNV culture, detection and sequencing, the COI and WNV sequence analyses, and the investigation into transovarial transmission. I recommend that you revise your manuscript to address these points in detail.

Please return the manuscript within 60 days; if you cannot complete the modification within this time period, please contact me. If you do not wish to modify the manuscript and prefer to submit it to another journal, notify me immediately so that the manuscript may be formally withdrawn from consideration by Spectrum.

Revision Guidelines
To submit your modified manuscript, log into the submission site at https://spectrum.msubmit.net/cgi-bin/main.plex. Go to Author Tasks and click the appropriate manuscript title to begin. The information you entered when you first submitted the paper will be displayed; update this as necessary. Note the following requirements:

• Upload point-by-point responses to the issues raised by the reviewers in a file named "Response to Reviewers," NOT in your cover letter.
• Upload a compare copy of the manuscript (without figures) as a "Marked-Up Manuscript" file.
• Upload a clean .DOC/.DOCX version of the revised manuscript and remove the previous version.
• Each figure must be uploaded as a separate, editable, high-resolution file (TIFF or EPS preferred), and any multipanel figures must be assembled into one file.
• Any supplemental material intended for posting by ASM should be uploaded with their legends separate from the main manuscript. You can combine all supplemental material into one file (preferred) or split it into a maximum of 10 files with all associated legends included.

For complete guidelines on revision requirements, see our Submission and Review Process webpage. Submission of a paper that does not conform to guidelines may delay acceptance of your manuscript.

Data availability: ASM policy requires that data be available to the public upon online posting of the article, so please verify all links to sequence records, if present, and make sure that each number retrieves the full record of the data. If a new accession number is not linked or a link is broken, provide Spectrum production staff with the correct URL for the record. If the accession numbers for new data are not publicly accessible before the expected online posting of the article, publication may be delayed; please contact production staff (Spectrum@asmusa.org) immediately with the expected release date.

Publication Fees: For information on publication fees and which article types are subject to charges, visit our website. If your manuscript is accepted for publication and any fees apply, you will be contacted separately about payment during the production process; please follow the instructions in that e-mail. Arrangements for payment must be made before your article is published.

ASM Membership: Corresponding authors may join or renew ASM membership to obtain discounts on publication fees. Need to upgrade your membership level? Please contact Customer Service at Service@asmusa.org.

The ASM Journals program strives for constant improvement in our submission and publication process. Please tell us how we can improve your experience by taking this quick Author Survey.

Thank you for submitting your paper to Spectrum.

Sincerely,
My Phan
Editor
Microbiology Spectrum

Reviewer #1 (Comments for the Author):

Comments on the manuscript by Tabitha Wanjiru et al.)Spectrum04205-25) Detection and Characterization of West Nile Virus with evidence of Transovarial Transmission in the Coastal region, Kenya

The authors have performed a large survey for West Nile Virus in Kenyan mosquitoes. 1596 mosquito pools (organized by species and sex and location) were tested for WNV in cell culture, 10 positive pools were identified and the virus was sequenced. Two lineages were found, some variation in the genome was observed and used to calculated selection pressure. WNV was found in one male pool and the author use this as evidence of transovarial transmission. The following points should be considered by the authors.

Point 1. The WNV genome sequences, the mosquito COI sequences and the raw sequence data should have been deposited in GenBank and a short read archive at the time of submission of the manuscript.

Line 587 "The sequences of the viruses identified in this study have been submitted to GenBank with
accession numbers: PX619830, PX619831, PX619832, PX619833, PX619834, PX619835,
PX619836, PX619837, PX619838, PX619839."
I see that these sequences are deposited but have not been made accessible. It is not possible to adequately review a sequence-based paper without providing the sequences. These should have been made available at the time of submission.

The raw sequence data before genome assembly should be deposited in one of the short read archives.
The COI sequences used to classify the mosquitoes should be described in better detail and deposited in a public data base.

The raw data for the COI sequencing should be deposited in a short sequence archive.

Point 2. A summary table should be provided describing the 10 full genomes. The table should list genome ID, poolID, species, sex, plus all read metrics for the genome (total reads for pool, number of WNV reads). GenBank accession number for the raw reads and GenBank accession number for the final genome sequence.

Point 3. The authors report processing and testing of a very large number of mosquito pools. Line 175 "One thousand five hundred ninety-six (1,596) mosquito pools were homogenized..."

The authors need to provide a table with the details of all 1,596 pools (number of mosquitoes species, sex, collection location (latitude, longitude), collection date) and indicate the pools that yielded a genome (provide GenBank and NRA accession numbers) and the male mosquito pool that yield a genome. The number of mosquitoes in that pool should be clearly indicated.

As described, the authors write that 1596 pools of mosquitoes were tested for WNV. The logistics of performing 1596 cell culture tests should be presented. How was this performed? The cost of medium and plasticware alone would be substantial (I estimate >$200,000 just for single round of CPE testing) However the authors may have done this in batches and then deconvoluted positive pools or something like that.

The following details should be provided: what size of cell culture, number of cells, volume of medium, growth conditions, source of cells, volume of inoculum used for passage, source of medium and supplements, source of cells. How was this screening managed?. Was each pool tested only once (1596 cell cultures) or were multiple test performed. The cost and logistics of this work would be substantial and I don't see this described in any detail.

If testing one pool (three passages) takes 3 weeks, and 20 pools were tested per round that would be 1596/20 =79.8 batches = 240 weeks = 4.6 years of constant testing. This is impressive and it would be important to describe the details of how this was organized.

Line 348 "Ten (10) pools exhibited cytopathic effects (CPE) after three successive passages in Vero E6 cells. Infected cultures developed CPE by day 5 post-inoculation, characterized by cell rounding, detachment, and loss of the monolayer."
Photographs of uninfected and CPE displaying cells should be provided and list of features of CPE provided.

Point 4. WNV was detected by cell culture. The authors should add comments in the discussion about possible missed diagnosis using cell culture compared to PCR or NGS on pooled or individual mosquitoes. Also, if only 10 pools out of 1596 were positive this can give a frequency values for WNV infected mosquitoes. Using the number of mosquitoes/pool would allows an upper and lower bound to this estimate. This should be performed and a discussion of how these WNV frequencies compare to surveillance values using PCR or sequencing and surveillance values from other regions?

Point 5. Better description of the mosquito species determination should be provided including the following details:
A summary table for the species determination should be provided with Pool ID, (final genome accession number ID), total number of reads, number of COI reads found in that pool, accession number for the COI sequences identified and accessions

number for the raw sequencing data

How did the authors rule out mixed pools? Suppose there was a mixed species mosquito pool? What is the detection limit for an minor mosquito species in the pool? This should be discussed.

The primers sequences for the COI amplification should be provided.

The concordance of sequencing COI with visual taxonomy should be shown.

How many mosquitoes were present per pool? This should have been included in a master table.

The alignment used for species identification should be provided or the method used for identification should be better described. Simply stating (Line 340) "..and taxonomic classification using the 'What's in my Pot' (WIMP) workflow. " is not sufficient.

The reference sequences used to classify the COI sequences should be listed (GenBank accession numbers).

Does the QIMP workflow contain COI sequence from all known mosquito species? How are these validated? Because the COI sequences generated were not made available for review it is difficult to assess this classification quality.

Point 6 concerns the conclusion that this study provides evidence of transovarial transmission.
Title (line 1) "...with evidence of Transovarial Transmission..."
Line 22 "and evidence of transovarial transmission in Aedes aegypti was observed"
Line 32 'Notably, one Lineage 1a isolate from a male Aedes aegypti confirmed transovarial transmission. "
Line 35 "WNV in coastal Kenyan mosquitoes revealed transovarial transmission in Aedes aegypti. " and Line 549 "The recovery of a full genomic sequence of Lineage 1 from a male Aedes aegypti further supports the possibility of transovarial transmission within Kenyan populations, strengthening the inference that non-blood acquisition pathways contribute to local maintenance in the region."

How does this observation support the conclusion? What is that evidence that the pool yielding the genome was uniquely male mosquito? How many mosquitoes were in the pool? The details of sex determination for this species were not provided.

At best the data may be evidence of vertical transmission but the evidence that this is transovarial is weak and alternate possibilities (e.g. trans-egg transmission infection, venereal infection) need to be discussed. Alternate trivial explanations (e.g. contamination of a male pool with female mosquitoes needs to be ruled out. This statement "evidence of transovarial transmission" should be removed from the title, abstract and elsewhere and replaced with "evidence of vertical transmission, consistent with but not proving transovarial transmission".

Reviewer #2 (Comments for the Author):
Title: Detection and Characterization of West Nile Virus with evidence of Transovarial 1 Transmission in the Coastal region, Kenya
Corresponding Authors: Tabitha Wanjiru
I read with great interest the manuscript "Detection and Characterization of West Nile Virus with evidence of Transovarial 1 Transmission in the Coastal region, Kenya" by Wanjiru et al. submitted to the Spectrum Journal, and I congratulate the authors for their efforts and results.
The manuscript should be completely rewritten, as the title does not fit with the introduction, discussion and conclusion. I suggest as title :" New insight on the West Nile vector circuit in the coastal regions of Kenya" for example. The title talks about transovarial transmission, but the article does not seem to focus on this type of transmission. In the abstract does not clearly state the problematic and objective of this study. In the summary, how did you collect the mosquitoes?
If the author maintains his own title of the manuscript, I suggest to submit it as short report and to present only the method, result and discussion focusing essentially on this vector transmission. The introduction is too long, occupying most of the article; there are several unnecessary paragraphs; it should be drastically shortened. The introduction should follow a clear structure, properly presenting the problematic and the objectives. For example, provide the problematic related to knowing the presence of vertical transmission? And state what are the objective associated with vertical transmission. There are many interesting results, but these results are not discussed in the main body of the article. The majority of its results are not important for understanding the vertical transmission mechanism. But if the title is changed, these results are very relevant according to the new title. Please kindly put all species and genus names in italics, pages are need to be added.
Many passages need to be shortened and clarified to avoid confusion. After making the necessary revisions to improve the clarity of the study, grammatical accuracy, and methodological descriptions, and considering the value of the research presented. It is suggested that this article be accepted for publication after major revision.

Good look

Reviewer #3 (Comments for the Author):

This study sampled more than 14,000 mosquitoes across multiple sites in Kenya and detected WNV from two lineages in 10 mosquito pools, providing insights into the WNV landscape in East Africa. Virus characterisation was comprehensive, with sequence analysis and growth kinetics. The use of CPE for WNV identification without a parallel RT-qPCR screen raises concerns about detection sensitivity and potential bias.

Major comments:
1. WMV detection in this paper relied solely on observed CPE effects. What's the rationale for this rather than incorporating parallel RT-qPCR screening? Although culture-based detection can confirm the presence of live and infectious virus particles, RT-qPCR is much more sensitive and a standard approach.
2. All sequenced genomes were from viruses that produced CPE in Vero cells and were collected after 3 passages in Vero cells prior to sequencing. Could these steps introduce bias (i.e. adaptive mutations) in the selection pressure and the observed amino acid variation?
3. Were any negative controls included during the virus isolation and sequencing process?
4. What climate/season were the mosquitoes collected in? Is there a list of the pool information? How many pools were in different areas? What's the landscape like where the mosquitoes were collected? Urban/rural area?
5. Were all available genome sequences from GenBank selected for BEAST analysis? Was any down-sampling performed? As this may affect the robustness of the BEAST analysis, a more detailed description should be included.
6. Would any other possible transmission route (e.g. mating) be able to explain the detection of WNV in a male mosquito? Is transovarial transmission the only possible conclusion?
7. Although mosquitoes were pooled by species, sex and collection site, sample pooling reduces the granularity of the observation.
8. Were there any limitations in the study?

Minor comments:
Line 226: How did you identify reference sequences that are closely related to your viral sequences? Line 229: "Closely related gene sequences..."? How closely related? Did you use a threshold?
Trees: Could you add the corresponding accession numbers to the tips of your trees for your samples?
Were sequencing data (fastq) uploaded to any archive (e.g. SRA)?

Revision

Title: Detection and Characterization of West Nile Virus with evidence of Transovarial 1 Transmission in the Coastal region, Kenya

Corresponding Authors: Tabitha Wanjiru

Report January 2026:

I read with great interest the manuscript "Detection and Characterization of West Nile Virus with evidence of Transovarial 1 Transmission in the Coastal region, Kenya" by Wanjiru et al. submitted to the Spectrum Journal, and I congratulate the authors for their efforts and results.

The manuscript should be completely rewritten, as the title does not fit with the introduction, discussion and conclusion. I suggest as title :" New insight on the West Nile vector circuit in the coastal regions of Kenya" for example. The title talks about transovarial transmission, but the article does not seem to focus on this type of transmission. In the abstract does not clearly state the problematic and objective of this study. In the summary, how did you collect the mosquitoes?

If the author maintains his own title of the manuscript, I suggest to submit it as short report and to present only the method, result and discussion focusing essentially on this vector transmission. The introduction is too long, occupying most of the article; there are several unnecessary paragraphs; it should be drastically shortened. The introduction should follow a clear structure, properly presenting the problematic and the objectives. For example, provide the problematic related to knowing the presence of vertical transmission? And state what are the objective associated with vertical transmission. There are many interesting results, but these results are not discussed in the main body of the article. The majority of its results are not important for understanding the vertical transmission mechanism. But if the title is changed, these results are very relevant according to the new title. Please kindly put all species and genus names in italics, pages are need to be added.

Many passages need to be shortened and clarified to avoid confusion. After making the necessary revisions to improve the clarity of the study, grammatical accuracy, and methodological descriptions, and considering the value of the research presented, it is suggested that this article be accepted for publication.

Good look

Reviewer #1 (Comments for the Author):

Comments on the manuscript by Tabitha Wanjiru et al.)Spectrum04205-25) Detection and Characterization of West Nile Virus with evidence of Transovarial Transmission in the Coastal region, Kenya

The authors have performed a large survey for West Nile Virus in Kenyan mosquitoes. 1596 mosquito pools (organized by species and sex and location) were tested for WNV in cell culture, 10 positive pools were identified and the virus was sequenced. Two lineages were found, some variation in the genome was observed and used to calculated selection pressure. WNV was found in one male pool and the author use this as evidence of transovarial transmission. The following points should be considered by the authors.

Point 1. The WNV genome sequences, the mosquito COI sequences and the raw sequence data should have been deposited in GenBank and a short read archive at the time of submission of the manuscript.

Line 587 "The sequences of the viruses identified in this study have been submitted to GenBank with
accession numbers: PX619830, PX619831, PX619832, PX619833, PX619834, PX619835, PX619836, PX619837, PX619838, PX619839."

I see that these sequences are deposited but have not been made accessible. It is not possible to adequately review a sequence-based paper without providing the sequences. These should have been made available at the time of submission.

The raw sequence data before genome assembly should be deposited in one of the short read archives.

Response

In the revised manuscript, all WNV genome sequences have now been made publicly accessible in GenBank, and the associated raw sequencing reads have been deposited in the Short Read Archive (SRA).

The COI sequences used to classify the mosquitoes should be described in better detail and deposited in a public data base.

Response

We expanded the description of COI sequencing, including methodology and accession details, and ensured that these sequences are also deposited in a public repository. These updates are now described in Lines 629–635.

The raw data for the COI sequencing should be deposited in a short sequence archive.

Response

The raw data have been deposited in the SRA and has been indicated in Line 629-635.

Point 2. A summary table should be provided describing the 10 full genomes. The table should list genome ID, poolID, species, sex, plus all read metrics for the genome (total reads for pool, number of WNV reads). GenBank accession number for the raw reads and GenBank accession number for the final genome sequence.

Response

To improve clarity and data accessibility, we have included a comprehensive summary table (Table 2) listing each genome alongside pool ID, mosquito species, sex, sequencing metrics (total reads and WNV reads), and accession numbers for both raw and assembled data. (Line 382)

Point 3. The authors report processing and testing of a very large number of mosquito pools. Line 175 "One thousand five hundred ninety-six (1,596) mosquito pools were homogenized..."

The authors need to provide a table with the details of all 1,596 pools (number of mosquitoes species, sex, collection location (latitude, longitude), collection date) and indicate the pools that yielded a genome (provide GenBank and NRA accession numbers) and the male mosquito pool that yield a genome. The number of mosquitoes in that pool should be clearly indicated.

Response

We have now included a detailed dataset (Supplementary Material 1) describing all 1,596 mosquito pools, including species, sex, collection location, and dates. Positive pools are clearly highlighted in red.

As described, the authors write that 1596 pools of mosquitoes were tested for WNV. The logistics of performing 1596 cell culture tests should be presented. How was this performed? The cost of medium and plasticware alone would be substantial (I estimate >$200,000 just for single round of CPE testing) However the authors may have done this in batches and then deconvoluted positive pools or something like that.

Response

To address concerns about feasibility and logistics, we have expanded the Methods section. Lines 195–199.

The following details should be provided: what size of cell culture, number of cells, volume of medium, growth conditions, source of cells, volume of inoculum used for passage, source of medium and supplements, source of cells. How was this screening managed?. Was each pool tested only once (1596 cell cultures) or were multiple test performed. The cost and logistics of this work would be substantial and I don't see this described in any detail.

Response

The workflow in detail, including cell culture conditions, screening strategy, batching of samples, and overall organization of the experiments have been described in detail in Line 181–203.

If testing one pool (three passages) takes 3 weeks, and 20 pools were tested per round that would be 1596/20 =79.8 batches = 240 weeks = 4.6 years of constant testing. This is impressive and it would be important to describe the details of how this was organized.

Response

Screening of mosquito pools was performed in batches, with approximately 220 mosquito pools processed per week. Each 24-well plate accommodated 22 mosquito pool samples, with the remaining wells serving as negative controls. Each mosquito pool was tested once during the primary screening, and cultures exhibiting CPE were harvested for further analysis. Lines 195–199.

Line 348 "Ten (10) pools exhibited cytopathic effects (CPE) after three successive passages in Vero E6 cells. Infected cultures developed CPE by day 5 post-inoculation, characterized by cell rounding, detachment, and loss of the monolayer."
Photographs of uninfected and CPE displaying cells should be provided and list of features of CPE provided.

Response

We have included representative images of cytopathic effects (CPE) in Figure 2 and expanded the description of observed morphological changes.

Point 4. WNV was detected by cell culture. The authors should add comments in the discussion about possible missed diagnosis using cell culture compared to PCR or NGS on pooled or individual mosquitoes. Also, if only 10 pools out of 1596 were positive this can give a frequency values for WNV infected mosquitoes. Using the number of mosquitoes/pool would allows an upper and lower bound to this estimate. This should be performed and a discussion of how these WNV frequencies compare to surveillance values using PCR or sequencing and surveillance values from other regions?

Response

We acknowledge that cell culture-based detection may underestimate viral prevalence compared to molecular methods such as RT-PCR. We have added a detailed discussion (Lines 573–582) addressing this limitation, including potential under-detection and comparison with other surveillance approaches.

Point 5. Better description of the mosquito species determination should be provided including the following details:
A summary table for the species determination should be provided with Pool ID, (final genome accession number ID), total number of reads, number of COI reads found in that pool, accession number for the COI sequences identified and accessions number for the raw sequencing data

Response

We have significantly expanded this section to improve clarity and reproducibility. A new summary table (Table 4) now presents COI sequencing results, including read counts and accession numbers. (Line 486)

How did the authors rule out mixed pools? Suppose there was a mixed species mosquito pool? What is the detection limit for an minor mosquito species in the pool? This should be discussed.

Response

We have included a discussion (Lines 583–588) addressing the possibility of mixed-species pools and the detection limits for minor species, acknowledging this as a limitation.

The primers sequences for the COI amplification should be provided.

Response

Primer sequences used for COI amplification are provided in Table 1. (Line 358)

The concordance of sequencing COI with visual taxonomy should be shown.

Response

We have also demonstrated concordance between morphological and molecular identification and listed the reference sequences used for classification in Table 4 (Line 486)

How many mosquitoes were present per pool? This should have been included in a master table.

Response

We have included this information in Supplementary Material 1, which now serves as a comprehensive master table for all mosquito pools analyzed in the study.

The alignment used for species identification should be provided or the method used for identification should be better described. Simply stating (Line 340) "..and taxonomic classification using the 'What's in my Pot' (WIMP) workflow. " is not sufficient.

The reference sequences used to classify the COI sequences should be listed (GenBank accession numbers).

Does the QIMP workflow contain COI sequence from all known mosquito species? How are these validated? Because the COI sequences generated were not made available for review it is difficult to assess this classification quality.

In the revised manuscript, we have substantially expanded the methodological description (Lines 339–355) to provide a clearer and more reproducible account of the approach used. We have included the accession numbers of the COI references used.

Point 6 concerns the conclusion that this study provides evidence of transovarial transmission.
Title (line 1) "...with evidence of Transovarial Transmission..."
Line 22 "and evidence of transovarial transmission in Aedes aegypti was observed"
Line 32 'Notably, one Lineage 1a isolate from a male Aedes aegypti confirmed transovarial transmission. "
Line 35 "WNV in coastal Kenyan mosquitoes revealed transovarial transmission in Aedes

aegypti. " and Line 549 "The recovery of a full genomic sequence of Lineage 1 from a male Aedes aegypti further supports the possibility of transovarial transmission within Kenyan populations, strengthening the inference that non-blood acquisition pathways
contribute to local maintenance in the region."

How does this observation support the conclusion? What is that evidence that the pool yielding the genome was uniquely male mosquito? How many mosquitoes were in the pool? The details of sex determination for this species were not provided.

At best the data may be evidence of vertical transmission but the evidence that this is transovarial is weak and alternate possibilities (e.g. trans-egg transmission infection, venereal infection) need to be discussed. Alternate trivial explanations (e.g. contamination of a male pool with female mosquitoes needs to be ruled out. This statement "evidence of transovarial transmission" should be removed from the title, abstract and elsewhere and replaced with "evidence of vertical transmission, consistent with but not proving transovarial transmission".

Response

In response, we have revised the manuscript throughout to replace the term "transovarial transmission" with the more appropriate and cautious term "vertical transmission." This change has been applied consistently in the title, abstract, results, and discussion sections.

Reviewer #2 (Comments for the Author):
Title: Detection and Characterization of West Nile Virus with evidence of Transovarial 1 Transmission in the Coastal region, Kenya
Corresponding Authors: Tabitha Wanjiru
I read with great interest the manuscript "Detection and Characterization of West Nile Virus with evidence of Transovarial 1 Transmission in the Coastal region, Kenya" by Wanjiru et al. submitted to the Spectrum Journal, and I congratulate the authors for their efforts and results.

The manuscript should be completely rewritten, as the title does not fit with the introduction, discussion and conclusion. I suggest as title :" New insight on the West Nile vector circuit in the coastal regions of Kenya" for example. The title talks about transovarial transmission, but the article does not seem to focus on this type of transmission. In the abstract does not clearly state the problematic and objective of this study. In the summary, how did you collect the mosquitoes?

- With respect to the abstract, we agree that the study objective and context required clearer articulation. The abstract has been revised (Lines 20–25) to explicitly state the research problem, the objectives of the study, and the approach used.

- In addition, we have clarified the mosquito collection methodology in both the abstract and Methods section to ensure that readers can clearly understand how samples were obtained and processed.

- We have undertaken substantial revisions to improve coherence between the title, introduction, results, and discussion. The introduction has been streamlined, and the discussion has been reframed to better reflect the primary aim of arboviral surveillance, with vertical transmission presented as a secondary observation.

If the author maintains his own title of the manuscript, I suggest to submit it as short report and to present only the method, result and discussion focusing essentially on this vector transmission. The introduction is too long, occupying most of the article; there are several unnecessary paragraphs; it should be drastically shortened. The introduction should follow a clear structure, properly presenting the problematic and the objectives.

Response

Thank you for the comment. The primary objective of this study was arboviral surveillance. We have revised and streamlined the section to improve clarity, structure, and conciseness while retaining the relevant background.

For example, provide the problematic related to knowing the presence of vertical transmission? And state what are the objective associated with vertical transmission. There are many interesting results, but these results are not discussed in the main body of the article. The majority of its results are not important for understanding the vertical transmission mechanism. But if the title is changed, these results are very relevant according to the new title. Please kindly put all species and genus names in italics, pages are need to be added.

Response

Thank you for this concern. The manuscript does not extensively explore the mechanisms or implications of vertical transmission, and we have revised the text to frame the findings within the context of surveillance.

Many passages need to be shortened and clarified to avoid confusion. After making the necessary revisions to improve the clarity of the study, grammatical accuracy, and methodological descriptions, and considering the value of the research presented. It is suggested that this article be accepted for publication after major revision.

Response

In response, we have carefully revised the manuscript to improve clarity, conciseness, and overall readability.

Reviewer #3 (Comments for the Author):

This study sampled more than 14,000 mosquitoes across multiple sites in Kenya and detected WNV from two lineages in 10 mosquito pools, providing insights into the WNV landscape in East Africa. Virus characterisation was comprehensive, with sequence analysis and growth kinetics. The use of CPE for WNV identification without a parallel

RT-qPCR screen raises concerns about detection sensitivity and potential bias.

Major comments:
1. WMV detection in this paper relied solely on observed CPE effects. What's the rationale for this rather than incorporating parallel RT-qPCR screening? Although culture-based detection can confirm the presence of live and infectious virus particles, RT-qPCR is much more sensitive and a standard approach.

Response

We acknowledge that RT-qPCR is more sensitive and widely used for rapid detection, the aim of this surveillance study was to prioritize the detection of infectious virus rather than viral RNA alone.

2. All sequenced genomes were from viruses that produced CPE in Vero cells and were collected after 3 passages in Vero cells prior to sequencing. Could these steps introduce bias (i.e. adaptive mutations) in the selection pressure and the observed amino acid variation?

Response

Limiting to three passages minimizes cell culture–adaptive mutations while confirming that the observed CPE reflects genuine viral replication rather than a false-positive effect

3. Were any negative controls included during the virus isolation and sequencing process?

Response

In the revised manuscript, we have clarified this in the Methods section (Lines 196–197), where we describe the inclusion of uninfected cell culture controls during virus isolation to verify that any observed cytopathic effects were attributable to viral infection rather than external contamination or culture artifacts.

4. What climate/season were the mosquitoes collected in? Is there a list of the pool information? How many pools were in different areas? What's the landscape like where the mosquitoes were collected? Urban/rural area?

Response
We have included comprehensive information addressing these aspects in Supplementary Material 1, which serves as a master dataset for all mosquito pools analyzed in the study.

5. Were all available genome sequences from GenBank selected for BEAST analysis?

Was any down-sampling performed? As this may affect the robustness of the BEAST analysis, a more detailed description should be included.

Response

We have now expanded the Methods section (Lines 251–252) to explicitly describe the selection criteria, inclusion strategy, and any down-sampling procedures used. This clarification ensures transparency in dataset construction and supports the robustness and reproducibility of the BEAST analysis.

6. Would any other possible transmission route (e.g. mating) be able to explain the detection of WNV in a male mosquito? Is transovarial transmission the only possible conclusion?

Response
This concern has been addressed and changed from transovarial to vertical transmission in the whole document

7. Although mosquitoes were pooled by species, sex and collection site, sample pooling reduces the granularity of the observation.

Response
Thank you for the comment. In arboviral surveillance studies, pooling allow efficient screening of large numbers of samples while maintaining representation across key biological and ecological categories.

8. Were there any limitations in the study?
Response
We have addressed this concern by adding limitations paragraph in the revised manuscript (Lines 573–582).

Minor comments:
Line 226: How did you identify reference sequences that are closely related to your viral sequences? Line 229: "Closely related gene sequences..."? How closely related? Did you use a threshold?

Response
In the revised manuscript, we have expanded the Methods section (Lines 232–235) to clearly describe how closely related reference sequences were identified.
Trees: Could you add the corresponding accession numbers to the tips of your trees for your samples?

Response
Accessions have added to the tips

Were sequencing data (fastq) uploaded to any archive (e.g. SRA)?

Response

All raw sequencing data (FASTQ files) generated in this study have been deposited in a public repository, specifically the Sequence Read Archive (SRA), to ensure full transparency and accessibility.

Re: Spectrum04205-25R1 (**Detection and Characterization of West Nile Virus with evidence of Transovarial Transmission in Coastal Kenya**)

Dear Dr. Tabitha Wanjiru:

Thank you for submitting the revision. Below you will find the reviewer comments and instructions from the Spectrum editorial office.

Please return the manuscript within 60 days; if you cannot complete the modification within this time period, please contact me. If you do not wish to modify the manuscript and prefer to submit it to another journal, notify me immediately so that the manuscript may be formally withdrawn from consideration by Spectrum.

Revision Guidelines
To submit your modified manuscript, log into the submission site at https://spectrum.msubmit.net/cgi-bin/main.plex. Go to Author Tasks and click the appropriate manuscript title to begin. The information you entered when you first submitted the paper will be displayed; update this as necessary. Note the following requirements:

• Upload point-by-point responses to the issues raised by the reviewers in a file named "Response to Reviewers," NOT in your cover letter.
• Upload a compare copy of the manuscript (without figures) as a "Marked-Up Manuscript" file.
• Upload a clean .DOC/.DOCX version of the revised manuscript and remove the previous version.
• Each figure must be uploaded as a separate, editable, high-resolution file (TIFF or EPS preferred), and any multipanel figures must be assembled into one file.
• Any supplemental material intended for posting by ASM should be uploaded with their legends separate from the main manuscript. You can combine all supplemental material into one file (preferred) or split it into a maximum of 10 files with all associated legends included.

For complete guidelines on revision requirements, see our Submission and Review Process webpage. Submission of a paper that does not conform to guidelines may delay acceptance of your manuscript.

Data availability: ASM policy requires that data be available to the public upon online posting of the article, so please verify all links to sequence records, if present, and make sure that each number retrieves the full record of the data. If a new accession number is not linked or a link is broken, provide Spectrum production staff with the correct URL for the record. If the accession numbers for new data are not publicly accessible before the expected online posting of the article, publication may be delayed; please contact production staff (Spectrum@asmusa.org) immediately with the expected release date.

Publication Fees: For information on publication fees and which article types are subject to charges, visit ourwebsite. If your manuscript is accepted for publication and any fees apply, you will be contacted separately about payment during the production process; please follow the instructions in that e-mail. Arrangements for payment must be made before your article is published.

ASM Membership: Corresponding authors may join or renew ASM membership to obtain discounts on publication fees. Need to upgrade your membership level? Please contact Customer Service at Service@asmusa.org.

The ASM Journals program strives for constant improvement in our submission and publication process. Please tell us how we can improve your experience by taking this quick Author Survey.

Thank you for submitting your paper to Spectrum.

Sincerely,
My Phan
Editor
Microbiology Spectrum

Reviewer #1 (Comments for the Author):

The authors have adequately addressed my previous concerns and the manuscript is much improved.

Reviewer #3 (Comments for the Author):

Since RT-qPCR was not used for primary detection, the manuscript title may benefit from a minor adjustment, as "detection" is

more typically used for molecular or antigen-based assays, whereas cell culture is more commonly associated with isolation. Tips of the tree. The order of the accession number/date/country is inconsistent between samples from this study and external sequences.

Reviewer's Response

Reviewer #1

Comment: The authors have adequately addressed my previous concerns and the manuscript is much improved.

Response: We thank the reviewer for their positive evaluation and for confirming that our previous revisions have adequately addressed their concerns.

Reviewer #3

Comment: Since RT-qPCR was not used for primary detection, the manuscript title may benefit from a minor adjustment, as "detection" is more typically used for molecular or antigen-based assays, whereas cell culture is more commonly associated with isolation.

Response: We have revised the title to more accurately reflect our methodology. The title now reads:

"Isolation and Molecular Characterization of West Nile Virus with Evidence of Vertical Transmission in the Coastal Region, Kenya"

Comment: Tips of the tree. The order of the accession number/date/country is inconsistent between samples from this study and external sequences.

Response: We have standardized the tip labels throughout the phylogenetic tree to follow a consistent format (Accession number/Country/Date) for both study samples and external reference sequences, ensuring uniformity across the entire tree.

Re: Spectrum04205-25R2 (**Detection and Characterization of West Nile Virus with evidence of Transovarial Transmission in Coastal Kenya**)

Dear Dr. Tabitha Wanjiru:

Congratulations! Your manuscript has been accepted, and I am forwarding it to the ASM production staff for publication. Your paper will first be checked to make sure all elements meet the technical requirements. ASM staff will contact you if anything needs to be revised before copyediting and production can begin. Otherwise, you will be notified when your proofs are ready to be viewed.

Data Availability: ASM policy requires that data be available to the public upon online posting of the article, so please verify all links to sequence records, if present, and make sure that each number retrieves the full record of the data. If a new accession number is not linked or a link is broken, provide production staff with the correct URL for the record. If the accession numbers for new data are not publicly accessible before the expected online posting of the article, publication may be delayed; please contact ASM production staff immediately with the expected release date.

Publication Fees: For information on publication fees and which article types have charges, please visit our website. We have partnered with Copyright Clearance Center (CCC) to collect author charges. If fees apply to your paper, you will receive a message from no-reply@copyright.com with further instructions. For questions related to paying charges through RightsLink, please contact CCC at ASM_Support@copyright.com or toll free at +1-877-622-5543. CCC makes every attempt to respond to all emails within 24 hours.

ASM Membership: Corresponding authors may join or renew ASM membership to obtain discounts on publication fees. Need to upgrade your membership level? Please contact Customer Service at Service@asmusa.org.

Embargo Policy: A press release may be issued as soon as the manuscript is posted on the Spectrum Latest Articles webpage. The corresponding author will receive an email with the subject line "ASM Journals Author Services Notification" when the article is available online.

Cover Image Submissions: If you would like to submit a potential Cover Image, please email a file and a short legend to spectrum@asmusa.org. Please note that we can only consider images that (i) the authors created or own and (ii) have not been previously published. By submitting, you agree that the image can be used under the same terms as the published article. Image File requirements: TIF/EPS, 7.5 inches wide by 8.25 inches tall (at least 2,250 pixels wide by 2,475 pixels tall), minimum 300 dpi resolution (600 dpi preferred), RGB, and no figure elements, e.g., arrows or panel labels. The legend should be a short description of the image, 1-2 sentences recommended. Please download and use this interactive template in Adobe the https://journals.asm.org/pb-assets/html/asm-cover-sizing-tool.html to ensure that your proposed cover image meets our size requirements (https://journals.asm.org/pb-assets/pdf-text-excel-files/ASM-Interactive-Sizing-Cover-Template-1715689791.pdf).

The ASM Journals program strives for constant improvement in our submission and publication process. Please tell us how we can improve your experience by taking this quick Author Survey.

Thank you for submitting your paper to Spectrum.

Sincerely,
My Phan
Editor
Microbiology Spectrum
