# Supplementary material for: Isolation and molecular characterization of West Nile Virus with evidence of vertical transmission in the Coastal region, Kenya
Source: Microbiol Spectr. 2026 Aug 25;14(10):e04205-25. doi: 10.1128/spectrum.04205-25 (PMC13688926; doi:10.1128/spectrum.04205-25)
Supplement: Supplementary file 2 [file spectrum.04205-25-s0001.pdf]

Sample Metadata

Coll. Date	Area	Pool number	Sex	Pool Size	*Species*	Target Pathogen	Results
3/16/2023	Isiolo	ISL/S5/43229	F	3	*Cx.annulioris*	Flavi/Alphavirus	Negative
3/16/2023	Isiolo	ISL/S5/43230	M	1	*Cx.annulioris*	Flavi/Alphavirus	Negative
3/16/2023	Isiolo	ISL/S5/43231	F	26	*Cx.univittatus*	Flavi/Alphavirus	Negative
3/16/2023	Isiolo	ISL/S5/43232	F	1	*Ae.aegypti*	Flavi/Alphavirus	Negative
3/16/2023	Isiolo	ISL/S5/43233	F	1	*Cx.pipiens*	Flavi/Alphavirus	Negative
3/16/2023	Isiolo	ISL/S5/43234	M	1	*Cx.pipiens*	Flavi/Alphavirus	Negative
3/16/2023	Isiolo	ISL/S5/43235	F	25	*An.gambiae*	Flavi/Alphavirus	Negative
3/16/2023	Isiolo	ISL/S5/43236	F	15	*An.gambiae*	Flavi/Alphavirus	Negative
3/16/2023	Isiolo	ISL/S5/43237	F	1	*Cx.theileri*	Flavi/Alphavirus	Negative
3/16/2023	Isiolo	ISL/S5/43238	F	7	*Cx.univittatus*	Flavi/Alphavirus	Negative
3/16/2023	Isiolo	ISL/S5/43239	F	6	*An.gambiae*	Flavi/Alphavirus	Negative
3/16/2023	Isiolo	ISL/S5/43240	F	1	*Cx.duttoni*	Flavi/Alphavirus	Negative
3/16/2023	Isiolo	ISL/S5/43241	F	1	*Cx.bitaeniorhynchus*	Flavi/Alphavirus	Negative
3/16/2023	Isiolo	ISL/S5/43242	F	1	*Cx.pipiens*	Flavi/Alphavirus	Negative
3/16/2023	Isiolo	ISL/S5/43243	F	4	*Cx.univittatus*	Flavi/Alphavirus	Negative
3/16/2023	Isiolo	ISL/S5/43244	M	3	*Cx.univittatus*	Flavi/Alphavirus	Negative
3/16/2023	Isiolo	ISL/S5/43245	M	5	*An.gambiae*	Flavi/Alphavirus	Negative
3/16/2023	Isiolo	ISL/S5/43246	F	8	*An.gambiae*	Flavi/Alphavirus	Negative
3/16/2023	Isiolo	ISL/S5/43247	F	1	*An.pretoriensis*	Flavi/Alphavirus	Negative
3/16/2023	Isiolo	ISL/S5/43248	F	5	*An.gambiae*	Flavi/Alphavirus	Negative
3/16/2023	Isiolo	ISL/S5/43249	F	1	*Cx.pipiens*	Flavi/Alphavirus	Negative
3/16/2023	Isiolo	ISL/S5/43250	M	1	*Cx.pipiens*	Flavi/Alphavirus	Negative
3/16/2023	Isiolo	ISL/S5/43251	F	2	*Cx.univittatus*	Flavi/Alphavirus	Negative
3/16/2023	Isiolo	ISL/S5/43252	M	5	*Cx.univittatus*	Flavi/Alphavirus	Negative
3/16/2023	Isiolo	ISL/S5/43253	F	10	*An.gambiae*	Flavi/Alphavirus	Negative
3/16/2023	Isiolo	ISL/S5/43254	M	2	*An.gambiae*	Flavi/Alphavirus	Negative
3/16/2023	Isiolo	ISL/S5/43255	F	2	*An.gambiae*	Flavi/Alphavirus	Negative
3/16/2023	Isiolo	ISL/S5/43256	F	1	*Cx.univittatus*	Flavi/Alphavirus	Negative
3/16/2023	Isiolo	ISL/S5/43257	F	1	*Cx.pipiens*	Flavi/Alphavirus	Negative
3/16/2023	Isiolo	ISL/S5/43258	F	1	*Cx.bitaeniorhynchus*	Flavi/Alphavirus	Negative
3/16/2023	Isiolo	ISL/S5/43259	F	2	*Cx.pipiens*	Flavi/Alphavirus	Negative
3/16/2023	Isiolo	ISL/S5/43260	M	4	*Cx.univittatus*	Flavi/Alphavirus	Negative
3/16/2023	Isiolo	ISL/S5/43261	F	1	*Cx.univittatus*	Flavi/Alphavirus	Negative
3/16/2023	Isiolo	ISL/S5/43262	M	1	*An.gambiae*	Flavi/Alphavirus	Negative
3/16/2023	Isiolo	ISL/S5/43263	F	8	*An.gambiae*	Flavi/Alphavirus	Negative
3/16/2023	Isiolo	ISL/S5/43264	F	1	*An.pretoriensis*	Flavi/Alphavirus	Negative
3/16/2023	Isiolo	ISL/S5/43265	M	1	*Cx.pipiens*	Flavi/Alphavirus	Negative
3/16/2023	Isiolo	ISL/S5/43266	M	1	*An.gambiae*	Flavi/Alphavirus	Negative
3/16/2023	Isiolo	ISL/S5/43267	F	15	*An.gambiae*	Flavi/Alphavirus	Negative
3/16/2023	Isiolo	ISL/S5/43268	F	8	*Cx.univittatus*	Flavi/Alphavirus	Negative
3/16/2023	Isiolo	ISL/S5/43269	M	1	*Cx.univittatus*	Flavi/Alphavirus	Negative
3/16/2023	Isiolo	ISL/S5/43270	F	1	*Cx.theileri*	Flavi/Alphavirus	Negative
3/16/2023	Isiolo	ISL/S5/43271	F	1	*Cx.bitaeniorhynchus*	Flavi/Alphavirus	Negative
3/16/2023	Isiolo	ISL/S5/43272	F	6	*An.gambiae*	Flavi/Alphavirus	Negative
3/16/2023	Isiolo	ISL/S5/43273	M	2	*Cx.pipiens*	Flavi/Alphavirus	Negative
3/16/2023	Isiolo	ISL/S5/43274	M	4	*Cx.univittatus*	Flavi/Alphavirus	Negative
3/16/2023	Isiolo	ISL/S5/43275	F	13	*Cx.univittatus*	Flavi/Alphavirus	Negative
3/16/2023	Isiolo	ISL/S5/43276	F	7	*An.gambiae*	Flavi/Alphavirus	Negative
3/16/2023	Isiolo	ISL/S5/43277	F	1	*Cx.univittatus*	Flavi/Alphavirus	Negative

3/16/2023	Isiolo	ISL/S5/43278	M	3	*Cx.univittatus*	Flavi/Alphavirus	Negative
3/17/2023	Isiolo	ISL/S6/43279	F	2	*Cx.annulioris*	Flavi/Alphavirus	Negative
3/17/2023	Isiolo	ISL/S6/43280	F	2	*An.pretoriensis*	Flavi/Alphavirus	Negative
3/17/2023	Isiolo	ISL/S6/43281	F	3	*An.gambiae*	Flavi/Alphavirus	Negative
3/17/2023	Isiolo	ISL/S6/43282	F	16	*Cx.univittatus*	Flavi/Alphavirus	Negative
3/17/2023	Isiolo	ISL/S6/43283	F	1	*An.funestus*	Flavi/Alphavirus	Negative
3/17/2023	Isiolo	ISL/S6/43284	F	1	*An.gambiae*	Flavi/Alphavirus	Negative
3/17/2023	Isiolo	ISL/S6/43285	F	1	*An.pretoriensis*	Flavi/Alphavirus	Negative
3/17/2023	Isiolo	ISL/S6/43286	F	2	*An.gambiae*	Flavi/Alphavirus	Negative
3/17/2023	Isiolo	ISL/S6/43287	F	3	*Cx.univittatus*	Flavi/Alphavirus	Negative
3/17/2023	Isiolo	ISL/S6/43288	F	1	*An.pretoriensis*	Flavi/Alphavirus	Negative
3/17/2023	Isiolo	ISL/S6/43289	M	1	*An.pretoriensis*	Flavi/Alphavirus	Negative
3/17/2023	Isiolo	ISL/S6/43290	F	7	*Cx.univittatus*	Flavi/Alphavirus	Negative
3/17/2023	Isiolo	ISL/S6/43291	F	1	*Cx.pipiens*	Flavi/Alphavirus	Negative
3/17/2023	Isiolo	ISL/S6/43292	F	2	*Cx.univittatus*	Flavi/Alphavirus	Negative
3/17/2023	Isiolo	ISL/S6/43293	F	1	*An.pretoriensis*	Flavi/Alphavirus	Negative
3/17/2023	Isiolo	ISL/S6/43294	F	2	*An.gambiae*	Flavi/Alphavirus	Negative
3/17/2023	Isiolo	ISL/S6/43295	F	5	*Cx.univittatus*	Flavi/Alphavirus	Negative
3/17/2023	Isiolo	ISL/S6/43296	M	1	*Cx.univittatus*	Flavi/Alphavirus	Negative
3/17/2023	Isiolo	ISL/S6/43297	F	3	*Cx.pipiens*	Flavi/Alphavirus	Negative
3/17/2023	Isiolo	ISL/S6/43298	F	15	*An.gambiae*	Flavi/Alphavirus	Negative
3/17/2023	Isiolo	ISL/S6/43299	F	24	*Cx.univittatus*	Flavi/Alphavirus	Negative
3/17/2023	Isiolo	ISL/S6/43300	F	4	*An.pretoriensis*	Flavi/Alphavirus	Negative
3/17/2023	Isiolo	ISL/S6/43301	F	9	*An.gambiae*	Flavi/Alphavirus	Negative
3/17/2023	Isiolo	ISL/S6/43302	F	18	*Cx.univittatus*	Flavi/Alphavirus	Negative
3/17/2023	Isiolo	ISL/S6/43303	M	1	*Cx.univittatus*	Flavi/Alphavirus	Negative
3/17/2023	Isiolo	ISL/S6/43304	F	1	*Cx.pipiens*	Flavi/Alphavirus	Negative
3/17/2023	Isiolo	ISL/S6/43305	F	1	*Cx.bitaeniorhynchus*	Flavi/Alphavirus	Negative
3/17/2023	Isiolo	ISL/S6/43306	F	3	*Cx.univittatus*	Flavi/Alphavirus	Negative
3/17/2023	Isiolo	ISL/S6/43307	F	1	*Cx.pipiens*	Flavi/Alphavirus	Negative
3/17/2023	Isiolo	ISL/S6/43308	F	1	*An.pretoriensis*	Flavi/Alphavirus	Negative
3/17/2023	Isiolo	ISL/S6/43309	F	1	*An.gambiae*	Flavi/Alphavirus	Negative
3/18/2023	Isiolo	ISL/S8/43310	F	1	*Cx.univittatus*	Flavi/Alphavirus	Negative
3/18/2023	Isiolo	ISL/S8/43311	F	1	*Cx.univittatus*	Flavi/Alphavirus	Negative
3/18/2023	Isiolo	ISL/S8/43312	F	1	*An.gambiae*	Flavi/Alphavirus	Negative
3/18/2023	Isiolo	ISL/S8/43313	F	1	*Cx.univittatus*	Flavi/Alphavirus	Negative
3/18/2023	Isiolo	ISL/S8/43314	F	4	*Cx.univittatus*	Flavi/Alphavirus	Negative
3/18/2023	Isiolo	ISL/S8/43315	F	1	*An.funestus*	Flavi/Alphavirus	Negative
3/18/2023	Isiolo	ISL/S8/43316	F	1	*Cx.univittatus*	Flavi/Alphavirus	Negative
3/19/2023	Isiolo	ISL/S7/43317	F	2	*Cx.bitaeniorhynchus*	Flavi/Alphavirus	Negative
3/19/2023	Isiolo	ISL/S7/43318	F	1	*Cx.univittatus*	Flavi/Alphavirus	Negative
3/19/2023	Isiolo	ISL/S7/43319	F	1	*An.funestus*	Flavi/Alphavirus	Negative
3/19/2023	Isiolo	ISL/S7/43320	F	1	*Ae.aegypti*	Flavi/Alphavirus	Negative
3/19/2023	Isiolo	ISL/S7/43321	F	1	*Cx.annulioris*	Flavi/Alphavirus	Negative
3/19/2023	Isiolo	ISL/S7/43322	F	1	*An.funestus*	Flavi/Alphavirus	Negative
3/19/2023	Isiolo	ISL/S7/43323	F	1	*Cx.univittatus*	Flavi/Alphavirus	Negative
3/19/2023	Isiolo	ISL/S7/43324	M	1	*Cx.univittatus*	Flavi/Alphavirus	Negative
3/19/2023	Isiolo	ISL/S7/43325	M	1	*Ae.aegypti*	Flavi/Alphavirus	Negative
3/19/2023	Isiolo	ISL/S7/43326	F	1	*An.gambiae*	Flavi/Alphavirus	Negative
3/19/2023	Isiolo	ISL/S7/43327	F	7	*Cx.univittatus*	Flavi/Alphavirus	Negative

3/19/2023	Isiolo	ISL/S7/43328	M	1	*Cx.univittatus*	Flavi/Alphavirus	Negative
3/19/2023	Isiolo	ISL/S7/43329	F	1	*Cx.theileri*	Flavi/Alphavirus	Negative
3/19/2023	Isiolo	ISL/S7/43330	F	1	*An.pretoriensis*	Flavi/Alphavirus	Negative
3/19/2023	Isiolo	ISL/S7/43331	F	8	*Cx.univittatus*	Flavi/Alphavirus	Negative
3/19/2023	Isiolo	ISL/S7/43332	F	3	*Cx.univittatus*	Flavi/Alphavirus	Negative
3/19/2023	Isiolo	ISL/S7/43333	F	1	*Ae.aegypti*	Flavi/Alphavirus	Negative
3/19/2023	Isiolo	ISL/S7/43334	F	1	*An.funestus*	Flavi/Alphavirus	Negative
3/19/2023	Isiolo	ISL/S7/43335	F	1	*An.gambiae*	Flavi/Alphavirus	Negative
3/19/2023	Isiolo	ISL/S7/43336	M	1	*Cx.univittatus*	Flavi/Alphavirus	Negative
3/19/2023	Isiolo	ISL/S7/43337	F	1	*Ae.aegypti*	Flavi/Alphavirus	Negative
3/19/2023	Isiolo	ISL/S7/43338	F	1	*An.funestus*	Flavi/Alphavirus	Negative
3/19/2023	Isiolo	ISL/S7/43339	F	2	*An.pretoriensis*	Flavi/Alphavirus	Negative
3/19/2023	Isiolo	ISL/S7/43340	F	5	*Cx.univittatus*	Flavi/Alphavirus	Negative
3/19/2023	Isiolo	ISL/S7/43341	F	1	*Cx.theileri*	Flavi/Alphavirus	Negative
3/19/2023	Isiolo	ISL/S7/43342	F	2	*An.pretoriensis*	Flavi/Alphavirus	Negative
3/19/2023	Isiolo	ISL/S7/43343	F	5	*Cx.univittatus*	Flavi/Alphavirus	Negative
3/19/2023	Isiolo	ISL/S7/43344	F	1	*Cx.cinereus*	Flavi/Alphavirus	Negative
3/19/2023	Isiolo	ISL/S7/43345	F	1	*Cx.theileri*	Flavi/Alphavirus	Negative
3/19/2023	Isiolo	ISL/S7/43346	M	1	*Cx.theileri*	Flavi/Alphavirus	Negative
3/19/2023	Isiolo	ISL/S7/43347	F	5	*Cx.univittatus*	Flavi/Alphavirus	Negative
3/19/2023	Isiolo	ISL/S7/43348	M	5	*Cx.univittatus*	Flavi/Alphavirus	Negative
3/19/2023	Isiolo	ISL/S7/43349	F	1	*Cx.pipiens*	Flavi/Alphavirus	Negative
3/19/2023	Isiolo	ISL/S7/43350	F	1	*An.pretoriensis*	Flavi/Alphavirus	Negative
3/19/2023	Isiolo	ISL/S7/43351	F	2	*Cx.univittatus*	Flavi/Alphavirus	Negative
3/19/2023	Isiolo	ISL/S7/43352	F	1	*An.pretoriensis*	Flavi/Alphavirus	Negative
3/20/2023	Isiolo	ISL/S9/43353	F	11	*Cx.univittatus*	Flavi/Alphavirus	Negative
3/20/2023	Isiolo	ISL/S9/43354	F	1	*An.gambiae*	Flavi/Alphavirus	Negative
3/20/2023	Isiolo	ISL/S9/43355	F	1	*Cx.theileri*	Flavi/Alphavirus	Negative
3/20/2023	Isiolo	ISL/S9/43356	F	3	*Cx.univittatus*	Flavi/Alphavirus	Negative
3/20/2023	Isiolo	ISL/S9/43357	F	2	*Cx.pipiens*	Flavi/Alphavirus	Negative
3/20/2023	Isiolo	ISL/S9/43358	F	1	*An.pretoriensis*	Flavi/Alphavirus	Negative
3/20/2023	Isiolo	ISL/S9/43359	F	1	*An.gambiae*	Flavi/Alphavirus	Negative
3/20/2023	Isiolo	ISL/S9/43360	F	1	*An.funestus*	Flavi/Alphavirus	Negative
3/20/2023	Isiolo	ISL/S9/43361	F	3	*Cx.pipiens*	Flavi/Alphavirus	Negative
3/20/2023	Isiolo	ISL/S9/43362	F	11	*Cx.univittatus*	Flavi/Alphavirus	Negative
3/20/2023	Isiolo	ISL/S9/43363	M	1	*Cx.univittatus*	Flavi/Alphavirus	Negative
3/20/2023	Isiolo	ISL/S9/43364	F	1	*An.pretoriensis*	Flavi/Alphavirus	Negative
3/20/2023	Isiolo	ISL/S9/43365	F	4	*An.gambiae*	Flavi/Alphavirus	Negative
3/20/2023	Isiolo	ISL/S9/43366	F	8	*Cx.pipiens*	Flavi/Alphavirus	Negative
3/20/2023	Isiolo	ISL/S9/43367	F	5	*Cx.theileri*	Flavi/Alphavirus	Negative
3/20/2023	Isiolo	ISL/S9/43368	F	11	*Cx.univittatus*	Flavi/Alphavirus	Negative
3/20/2023	Isiolo	ISL/S9/43369	F	1	*Cx.duttoni*	Flavi/Alphavirus	Negative
3/20/2023	Isiolo	ISL/S9/43370	F	4	*Cx.pipiens*	Flavi/Alphavirus	Negative
3/20/2023	Isiolo	ISL/S9/43371	F	2	*Cx.theileri*	Flavi/Alphavirus	Negative
3/20/2023	Isiolo	ISL/S9/43372	F	1	*An.gambiae*	Flavi/Alphavirus	Negative
3/20/2023	Isiolo	ISL/S9/43373	F	11	*Cx.univittatus*	Flavi/Alphavirus	Negative
3/20/2023	Isiolo	ISL/S9/43374	F	1	*Cx.theileri*	Flavi/Alphavirus	Negative
3/20/2023	Isiolo	ISL/S9/43375	F	1	*An.gambiae*	Flavi/Alphavirus	Negative
3/20/2023	Isiolo	ISL/S9/43376	F	2	*Cx.pipiens*	Flavi/Alphavirus	Negative
3/20/2023	Isiolo	ISL/S9/43377	F	3	*Cx.univittatus*	Flavi/Alphavirus	Negative

3/20/2023	Isiolo	ISL/S9/43378	F	4	*Cx.univittatus*	Flavi/Alphavirus	Negative
3/20/2023	Isiolo	ISL/S9/43379	F	7	*Cx.pipiens*	Flavi/Alphavirus	Negative
3/20/2023	Isiolo	ISL/S9/43380	F	2	*An.gambiae*	Flavi/Alphavirus	Negative
3/20/2023	Isiolo	ISL/S9/43381	F	10	*Cx.univittatus*	Flavi/Alphavirus	Negative
3/20/2023	Isiolo	ISL/S9/43382	F	3	*Cx.theileri*	Flavi/Alphavirus	Negative
3/21/2023	Isiolo	ISL/S10/43383	F	1	*Cx.univittatus*	Flavi/Alphavirus	Negative
3/21/2023	Isiolo	ISL/S10/43384	F	1	*Cx.theileri*	Flavi/Alphavirus	Negative
3/21/2023	Isiolo	ISL/S10/43385	F	4	*Cx.univittatus*	Flavi/Alphavirus	Negative
3/21/2023	Isiolo	ISL/S10/43386	F	1	*Ae.aegypti*	Flavi/Alphavirus	Negative
3/21/2023	Isiolo	ISL/S10/43387	F	1	*An.gambiae*	Flavi/Alphavirus	Negative
3/21/2023	Isiolo	ISL/S10/43388	F	2	*Cx.theileri*	Flavi/Alphavirus	Negative
3/21/2023	Isiolo	ISL/S10/43389	F	6	*Cx.univittatus*	Flavi/Alphavirus	Negative
3/21/2023	Isiolo	ISL/S10/43390	M	1	*Cx.univittatus*	Flavi/Alphavirus	Negative
3/21/2023	Isiolo	ISL/S10/43391	F	1	*Cx.theileri*	Flavi/Alphavirus	Negative
3/21/2023	Isiolo	ISL/S10/43392	F	1	*An.funestus*	Flavi/Alphavirus	Negative
3/21/2023	Isiolo	ISL/S10/43393	F	7	*Cx.univittatus*	Flavi/Alphavirus	Negative
3/21/2023	Isiolo	ISL/S10/43394	M	1	*Cx.univittatus*	Flavi/Alphavirus	Negative
3/21/2023	Isiolo	ISL/S10/43395	F	4	*Cx.univittatus*	Flavi/Alphavirus	Negative
3/21/2023	Isiolo	ISL/S10/43396	M	1	*Cx.univittatus*	Flavi/Alphavirus	Negative
3/21/2023	Isiolo	ISL/S10/43397	F	1	*Cx.theileri*	Flavi/Alphavirus	Negative
3/21/2023	Isiolo	ISL/S10/43398	F	1	*Cx.pipiens*	Flavi/Alphavirus	Negative
3/21/2023	Isiolo	ISL/S10/43399	F	1	*An.gambiae*	Flavi/Alphavirus	Negative
3/21/2023	Isiolo	ISL/S10/43400	F	1	*Cx.theileri*	Flavi/Alphavirus	Negative
3/21/2023	Isiolo	ISL/S10/43401	F	10	*Cx.univittatus*	Flavi/Alphavirus	Negative
3/21/2023	Isiolo	ISL/S10/43402	F	4	*An.gambiae*	Flavi/Alphavirus	Negative
3/21/2023	Isiolo	ISL/S10/43403	M	1	*An.gambiae*	Flavi/Alphavirus	Negative
3/21/2023	Isiolo	ISL/S10/43404	F	2	*Cx.pipiens*	Flavi/Alphavirus	Negative
3/21/2023	Isiolo	ISL/S10/43405	F	10	*Cx.univittatus*	Flavi/Alphavirus	Negative
3/21/2023	Isiolo	ISL/S10/43406	F	3	*Ae.aegypti*	Flavi/Alphavirus	Negative
3/21/2023	Isiolo	ISL/S10/43407	M	3	*Ae.aegypti*	Flavi/Alphavirus	Negative
3/21/2023	Isiolo	ISL/S10/43408	M	7	*Culex spps*	Flavi/Alphavirus	Negative
3/21/2023	Isiolo	ISL/S10/43409	F	2	*An.pretoriensis*	Flavi/Alphavirus	Negative
3/21/2023	Isiolo	ISL/S10/43410	F	3	*An.gambiae*	Flavi/Alphavirus	Negative
3/21/2023	Isiolo	ISL/S10/43411	M	2	*Anopheles spps*	Flavi/Alphavirus	Negative
3/21/2023	Isiolo	ISL/S10/43412	F	2	*Cx.theileri*	Flavi/Alphavirus	Negative
3/21/2023	Isiolo	ISL/S10/43413	F	10	*Cx.univittatus*	Flavi/Alphavirus	Negative
6/18/2023	Malindi	MAL/S2/43414	F	1	*Cx.pipiens*	Flavi/Alphavirus	Negative
6/18/2023	Malindi	MAL/S2/43415	F	1	*Cx.univittatus*	Flavi/Alphavirus	Negative
6/18/2023	Malindi	MAL/S2/43416	F	1	*Ae.dentatus*	Flavi/Alphavirus	Negative
6/18/2023	Malindi	MAL/S2/43417	F	1	*Ae.aegypti*	Flavi/Alphavirus	Negative
6/18/2023	Malindi	MAL/S2/43418	F	2	*Ae.tricholabis*	Flavi/Alphavirus	Negative
6/18/2023	Malindi	MAL/S2/43419	F	9	*An.gambiae*	Flavi/Alphavirus	Negative
6/18/2023	Malindi	MAL/S2/43420	F	25	*Cx.annulioris*	Flavi/Alphavirus	Negative
6/18/2023	Malindi	MAL/S2/43421	F	25	*Cx.annulioris*	Flavi/Alphavirus	Negative
6/18/2023	Malindi	MAL/S2/43422	F	25	*Cx.annulioris*	Flavi/Alphavirus	Negative
6/18/2023	Malindi	MAL/S2/43423	F	25	*Cx.annulioris*	Flavi/Alphavirus	Negative
6/18/2023	Malindi	MAL/S2/43424	F	25	*Cx.annulioris*	Flavi/Alphavirus	Negative
6/18/2023	Malindi	MAL/S2/43425	F	25	*Ae.pembaensis*	Flavi/Alphavirus	Negative
6/18/2023	Malindi	MAL/S2/43426	F	25	*Ae.pembaensis*	Flavi/Alphavirus	Negative
6/18/2023	Malindi	MAL/S2/43427	F	25	*Ae.pembaensis*	Flavi/Alphavirus	Negative

6/18/2023	Malindi	MAL/S2/43428	F	25	*Ae.pembaensis*	Flavi/Alphavirus	Negative
6/18/2023	Malindi	MAL/S2/43429	F	25	*Ae.pembaensis*	Flavi/Alphavirus	Negative
6/18/2023	Malindi	MAL/S2/43430	F	25	*Ae.pembaensis*	Flavi/Alphavirus	Negative
6/18/2023	Malindi	MAL/S2/43431	F	25	*Ae.pembaensis*	Flavi/Alphavirus	Negative
6/18/2023	Malindi	MAL/S2/43432	F	25	*Ae.pembaensis*	Flavi/Alphavirus	Negative
6/18/2023	Malindi	MAL/S2/43433	F	25	*Ae.pembaensis*	Flavi/Alphavirus	Negative
6/18/2023	Malindi	MAL/S2/43434	F	25	*Ae.pembaensis*	Flavi/Alphavirus	Negative
6/18/2023	Malindi	MAL/S2/43435	F	25	*Ae.pembaensis*	Flavi/Alphavirus	Negative
6/18/2023	Malindi	MAL/S2/43436	F	25	*Ae.pembaensis*	Flavi/Alphavirus	Negative
6/18/2023	Malindi	MAL/S2/43437	F	25	*Ae.pembaensis*	Flavi/Alphavirus	Negative
6/18/2023	Malindi	MAL/S2/43438	F	25	*Ae.pembaensis*	Flavi/Alphavirus	Negative
6/18/2023	Malindi	MAL/S2/43439	F	25	*Ae.pembaensis*	Flavi/Alphavirus	Negative
6/18/2023	Malindi	MAL/S2/43440	F	25	*Ae.pembaensis*	Flavi/Alphavirus	Negative
6/18/2023	Malindi	MAL/S2/43441	F	25	*Ae.pembaensis*	Flavi/Alphavirus	Negative
6/18/2023	Malindi	MAL/S2/43442	F	25	*Ae.pembaensis*	Flavi/Alphavirus	Negative
6/18/2023	Malindi	MAL/S2/43443	F	2	*Cx.univittatus*	Flavi/Alphavirus	Negative
6/18/2023	Malindi	MAL/S2/43444	F	1	*Cx.pipiens*	Flavi/Alphavirus	Negative
6/18/2023	Malindi	MAL/S2/43445	F	2	*Ae.dentatus*	Flavi/Alphavirus	Negative
6/18/2023	Malindi	MAL/S2/43446	F	1	*Cx.univittatus*	Flavi/Alphavirus	Negative
6/18/2023	Malindi	MAL/S2/43447	F	25	*Cx.annulioris*	Flavi/Alphavirus	Negative
6/18/2023	Malindi	MAL/S2/43448	F	25	*Cx.annulioris*	Flavi/Alphavirus	Negative
6/18/2023	Malindi	MAL/S2/43449	F	25	*Cx.annulioris*	Flavi/Alphavirus	Negative
6/18/2023	Malindi	MAL/S2/43450	F	25	*Cx.annulioris*	Flavi/Alphavirus	Negative
6/18/2023	Malindi	MAL/S2/43451	F	25	*Ae.pembaensis*	Flavi/Alphavirus	Negative
6/18/2023	Malindi	MAL/S2/43452	F	25	*Ae.pembaensis*	Flavi/Alphavirus	Negative
6/18/2023	Malindi	MAL/S2/43453	F	25	*Ae.pembaensis*	Flavi/Alphavirus	Negative
6/18/2023	Malindi	MAL/S2/43454	F	25	*Ae.pembaensis*	Flavi/Alphavirus	Negative
6/18/2023	Malindi	MAL/S2/43455	F	25	*Ae.pembaensis*	Flavi/Alphavirus	Negative
6/18/2023	Malindi	MAL/S2/43456	F	25	*Ae.pembaensis*	Flavi/Alphavirus	Negative
6/18/2023	Malindi	MAL/S2/43457	F	25	*Ae.pembaensis*	Flavi/Alphavirus	Negative
6/18/2023	Malindi	MAL/S2/43458	F	25	*Ae.pembaensis*	Flavi/Alphavirus	Negative
6/18/2023	Malindi	MAL/S2/43459	F	25	*Ae.pembaensis*	Flavi/Alphavirus	Negative
6/18/2023	Malindi	MAL/S2/43460	F	25	*Ae.pembaensis*	Flavi/Alphavirus	Negative
6/18/2023	Malindi	MAL/S2/43461	F	21	*Ae.pembaensis*	Flavi/Alphavirus	Negative
6/19/2023	Malindi	MAL/S5/43462	F	9	*Cx.poicilipes*	Flavi/Alphavirus	Negative
6/19/2023	Malindi	MAL/S5/43463	F	3	*Cx.pipiens*	Flavi/Alphavirus	Negative
6/19/2023	Malindi	MAL/S5/43464	F	1	*Ae.sudanensis*	Flavi/Alphavirus	Negative
6/19/2023	Malindi	MAL/S5/43465	F	2	*Coq.aurites*	Flavi/Alphavirus	Negative
6/19/2023	Malindi	MAL/S5/43466	F	12	*Cx.annulioris*	Flavi/Alphavirus	Negative
6/19/2023	Malindi	MAL/S5/43467	F	10	*Ae.circumluteolus*	Flavi/Alphavirus	Negative
6/19/2023	Malindi	MAL/S5/43468	F	2	*Cx.univittatus*	Flavi/Alphavirus	Negative
6/19/2023	Malindi	MAL/S5/43469	F	3	*Mn.uniformis*	Flavi/Alphavirus	Negative
6/19/2023	Malindi	MAL/S5/43470	F	25	*Ae.pembaensis*	Flavi/Alphavirus	Negative
6/19/2023	Malindi	MAL/S5/43471	F	25	*Ae.pembaensis*	Flavi/Alphavirus	Negative
6/19/2023	Malindi	MAL/S5/43472	F	25	*Ae.pembaensis*	Flavi/Alphavirus	Negative
6/19/2023	Malindi	MAL/S5/43473	F	25	*Ae.pembaensis*	Flavi/Alphavirus	Negative
6/19/2023	Malindi	MAL/S5/43474	F	25	*Ae.pembaensis*	Flavi/Alphavirus	Negative
6/19/2023	Malindi	MAL/S5/43475	F	25	*Ae.pembaensis*	Flavi/Alphavirus	Negative
6/19/2023	Malindi	MAL/S5/43476	F	25	*Ae.pembaensis*	Flavi/Alphavirus	Negative
6/19/2023	Malindi	MAL/S5/43477	F	25	*Ae.pembaensis*	Flavi/Alphavirus	Negative

6/19/2023	Malindi	MAL/S5/43478	F	25	*Ae.pembaensis*	Flavi/Alphavirus	Negative
6/19/2023	Malindi	MAL/S5/43479	F	25	*Ae.pembaensis*	Flavi/Alphavirus	Negative
6/19/2023	Malindi	MAL/S5/43480	F	25	*Ae.pembaensis*	Flavi/Alphavirus	Negative
6/19/2023	Malindi	MAL/S5/43481	F	25	*Ae.pembaensis*	Flavi/Alphavirus	Negative
6/19/2023	Malindi	MAL/S5/43482	F	25	*Ae.pembaensis*	Flavi/Alphavirus	Negative
6/19/2023	Malindi	MAL/S5/43483	F	25	*Ae.pembaensis*	Flavi/Alphavirus	Negative
6/19/2023	Malindi	MAL/S5/43484	F	25	*Ae.pembaensis*	Flavi/Alphavirus	Negative
6/19/2023	Malindi	MAL/S5/43485	F	25	*Ae.pembaensis*	Flavi/Alphavirus	Negative
6/19/2023	Malindi	MAL/S5/43486	F	2	*Cx.pipiens*	Flavi/Alphavirus	Negative
6/19/2023	Malindi	MAL/S5/43487	F	7	*Cx.annulioris*	Flavi/Alphavirus	Negative
6/19/2023	Malindi	MAL/S5/43488	F	2	*Mn.uniformis*	Flavi/Alphavirus	Negative
6/19/2023	Malindi	MAL/S5/43489	F	2	*Ae.circumluteolus*	Flavi/Alphavirus	Negative
6/19/2023	Malindi	MAL/S5/43490	F	25	*Ae.pembaensis*	Flavi/Alphavirus	Negative
6/19/2023	Malindi	MAL/S5/43572	F	25	*Ae.pembaensis*	Flavi/Alphavirus	Negative
6/19/2023	Malindi	MAL/S5/43573	F	13	*Ae.pembaensis*	Flavi/Alphavirus	Negative
6/20/2023	Malindi	MAL/S7/43574	M	7	*Ae.aegypti*	Flavi/Alphavirus	Negative
6/20/2023	Malindi	MAL/S7/43575	F	11	*Ae.aegypti*	Flavi/Alphavirus	Negative
6/20/2023	Malindi	MAL/S7/43576	F	20	*Cx.annulioris*	Flavi/Alphavirus	Negative
6/20/2023	Malindi	MAL/S7/43577	F	2	*Ae.simpsoni*	Flavi/Alphavirus	Negative
6/20/2023	Malindi	MAL/S7/43578	F	1	*Cx.univittatus*	Flavi/Alphavirus	Negative
6/20/2023	Malindi	MAL/S7/43579	F	1	*An.gambiae*	Flavi/Alphavirus	Negative
6/20/2023	Malindi	MAL/S7/43580	F	25	*Cx.pipiens*	Flavi/Alphavirus	Negative
6/20/2023	Malindi	MAL/S7/43581	F	9	*Cx.pipiens*	Flavi/Alphavirus	Negative
6/20/2023	Malindi	MAL/S7/43582	F	1	*Cx.univittatus*	Flavi/Alphavirus	Negative
6/20/2023	Malindi	MAL/S7/43583	F	2	*Ae.metallicus*	Flavi/Alphavirus	Negative
6/20/2023	Malindi	MAL/S7/43584	F	4	*Uranotaenia spps*	Flavi/Alphavirus	Negative
6/20/2023	Malindi	MAL/S7/43585	F	1	*Eret.chryogaster*	Flavi/Alphavirus	Negative
6/20/2023	Malindi	MAL/S7/43586	F	25	*Ae.aegypti*	Flavi/Alphavirus	Negative
6/20/2023	Malindi	MAL/S7/43587	F	25	*Ae.aegypti*	Flavi/Alphavirus	Negative
6/20/2023	Malindi	MAL/S7/43588	F	25	*Ae.aegypti*	Flavi/Alphavirus	Negative
6/20/2023	Malindi	MAL/S7/43589	M	18	*Ae.aegypti*	Flavi/Alphavirus	Negative
6/20/2023	Malindi	MAL/S7/43590	F	25	*Cx.pipiens*	Flavi/Alphavirus	Negative
6/20/2023	Malindi	MAL/S7/43591	F	25	*Cx.pipiens*	Flavi/Alphavirus	Negative
6/20/2023	Malindi	MAL/S7/43592	F	20	*Cx.annulioris*	Flavi/Alphavirus	Negative
6/20/2023	Malindi	MAL/S7/43593	F	25	*Cx.annulioris*	Flavi/Alphavirus	Negative
6/20/2023	Malindi	MAL/S7/43594	F	11	*Cx.annulioris*	Flavi/Alphavirus	Negative
6/20/2023	Malindi	MAL/S7/43595	M	1	*Ae.metallicus*	Flavi/Alphavirus	Negative
6/20/2023	Malindi	MAL/S7/43596	F	1	*Ae.pembaensis*	Flavi/Alphavirus	Negative
6/20/2023	Malindi	MAL/S7/43597	M	21	*Ae.aegypti*	Flavi/Alphavirus	Negative
6/20/2023	Malindi	MAL/S7/43598	F	25	*Ae.aegypti*	Flavi/Alphavirus	Negative
6/20/2023	Malindi	MAL/S7/43599	F	25	*Ae.aegypti*	Flavi/Alphavirus	Negative
6/20/2023	Malindi	MAL/S7/43600	F	7	*Ae.aegypti*	Flavi/Alphavirus	Negative
6/20/2023	Malindi	MAL/S7/43601	F	25	*Cx.pipiens*	Flavi/Alphavirus	Negative
6/20/2023	Malindi	MAL/S7/43602	F	18	*Cx.pipiens*	Flavi/Alphavirus	Negative
6/20/2023	Malindi	MAL/S7/43603	F	2	*Cx.univittatus*	Flavi/Alphavirus	Negative
6/20/2023	Malindi	MAL/S7/43604	F	2	*Ae.simpsoni*	Flavi/Alphavirus	Negative
6/20/2023	Malindi	MAL/S7/43605	F	6	*Ae.aegypti*	Flavi/Alphavirus	Negative
6/20/2023	Malindi	MAL/S7/43606	F	11	*Cx.annulioris*	Flavi/Alphavirus	Negative
6/20/2023	Malindi	MAL/S7/43607	F	4	*Cx.rubinotus*	Flavi/Alphavirus	Negative
6/20/2023	Malindi	MAL/S7/43608	F	13	*Cx.pipiens*	Flavi/Alphavirus	Negative

6/20/2023	Malindi	MAL/S7/43609	F	2	*Cx.univittatus*	Flavi/Alphavirus	Negative
6/20/2023	Malindi	MAL/S7/43610	F	1	*Ae.aegypti*	Flavi/Alphavirus	Negative
6/20/2023	Malindi	MAL/S7/43611	F	6	*Cx.annulioris*	Flavi/Alphavirus	Negative
6/20/2023	Malindi	MAL/S7/43612	F	17	*Cx.pipiens*	Flavi/Alphavirus	Negative
6/20/2023	Malindi	MAL/S7/43613	F	24	*Cx.pipiens*	Flavi/Alphavirus	Negative
6/20/2023	Malindi	MAL/S7/43614	F	10	*Ae.aegypti*	Flavi/Alphavirus	Negative
6/20/2023	Malindi	MAL/S7/43615	F	3	*Cx.univittatus*	Flavi/Alphavirus	Negative
6/20/2023	Malindi	MAL/S7/43616	F	3	*An.gambiae*	Flavi/Alphavirus	Negative
6/20/2023	Malindi	MAL/S7/43617	F	10	*Ae.pembaensis*	Flavi/Alphavirus	Negative
6/20/2023	Malindi	MAL/S7/43618	F	25	*Cx.annulioris*	Flavi/Alphavirus	Negative
6/20/2023	Malindi	MAL/S7/43619	F	21	*Cx.univittatus*	Flavi/Alphavirus	Negative
6/20/2023	Malindi	MAL/S7/43620	F	1	*Ae.metallicus*	Flavi/Alphavirus	Negative
6/20/2023	Malindi	MAL/S7/43621	F	9	*Cx.annulioris*	Flavi/Alphavirus	Negative
6/20/2023	Malindi	MAL/S7/43622	F	1	*Cx.univittatus*	Flavi/Alphavirus	Negative
6/20/2023	Malindi	MAL/S7/43623	M	2	*Ae.aegypti*	Flavi/Alphavirus	Negative
6/20/2023	Malindi	MAL/S7/43624	F	21	*Ae.aegypti*	Flavi/Alphavirus	Negative
6/20/2023	Malindi	MAL/S7/43625	F	25	*Cx.pipiens*	Flavi/Alphavirus	Negative
6/20/2023	Malindi	MAL/S7/43626	F	20	*Cx.pipiens*	Flavi/Alphavirus	Negative
6/20/2023	Malindi	MAL/S7/43627	M	2	*Ae.aegypti*	Flavi/Alphavirus	Negative
6/20/2023	Malindi	MAL/S7/43628	F	25	*Ae.aegypti*	Flavi/Alphavirus	Negative
6/20/2023	Malindi	MAL/S7/43629	F	2	*Ae.simpsoni*	Flavi/Alphavirus	Negative
6/20/2023	Malindi	MAL/S7/43630	F	1	*Cx.univittatus*	Flavi/Alphavirus	Negative
6/20/2023	Malindi	MAL/S7/43631	F	17	*Cx.annulioris*	Flavi/Alphavirus	Negative
6/20/2023	Malindi	MAL/S7/43632	F	25	*Cx.pipiens*	Flavi/Alphavirus	Negative
6/20/2023	Malindi	MAL/S7/43633	F	25	*Cx.pipiens*	Flavi/Alphavirus	Negative
6/20/2023	Malindi	MAL/S7/43634	F	25	*Cx.pipiens*	Flavi/Alphavirus	Negative
6/20/2023	Malindi	MAL/S7/43635	F	7	*Cx.pipiens*	Flavi/Alphavirus	Negative
6/20/2023	Malindi	MAL/S7/43636	F	12	*Ae.aegypti*	Flavi/Alphavirus	Negative
6/20/2023	Malindi	MAL/S7/43637	F	4	*Cx.univittatus*	Flavi/Alphavirus	Negative
6/20/2023	Malindi	MAL/S7/43638	F	25	*Cx.annulioris*	Flavi/Alphavirus	Negative
6/20/2023	Malindi	MAL/S7/43639	F	6	*Cx.annulioris*	Flavi/Alphavirus	Negative
6/20/2023	Malindi	MAL/S7/43640	F	25	*Cx.pipiens*	Flavi/Alphavirus	Negative
6/20/2023	Malindi	MAL/S7/43641	F	25	*Cx.pipiens*	Flavi/Alphavirus	Negative
6/20/2023	Malindi	MAL/S7/43642	F	2	*Cx.pipiens*	Flavi/Alphavirus	Negative
6/20/2023	Malindi	MAL/S7/43643	F	2	*Cx.univittatus*	Flavi/Alphavirus	Negative
6/20/2023	Malindi	MAL/S7/43644	F	1	*Mn.uniformis*	Flavi/Alphavirus	Negative
6/20/2023	Malindi	MAL/S7/43645	F	2	*Uranotaenia spps*	Flavi/Alphavirus	Negative
6/20/2023	Malindi	MAL/S7/43646	F	2	*Ae.simpsoni*	Flavi/Alphavirus	Negative
6/20/2023	Malindi	MAL/S7/43647	F	2	*Cx.duttoni*	Flavi/Alphavirus	Negative
6/20/2023	Malindi	MAL/S7/43648	F	14	*Cx.annulioris*	Flavi/Alphavirus	Negative
6/20/2023	Malindi	MAL/S7/43649	F	25	*Ae.aegypti*	Flavi/Alphavirus	Negative
6/20/2023	Malindi	MAL/S7/43650	F	25	*Ae.aegypti*	Flavi/Alphavirus	Negative
6/20/2023	Malindi	MAL/S7/43651	F	25	*Ae.aegypti*	Flavi/Alphavirus	Negative
6/20/2023	Malindi	MAL/S7/43652	F	25	*Ae.aegypti*	Flavi/Alphavirus	Negative
6/20/2023	Malindi	MAL/S7/43653	F	3	*Ae.aegypti*	Flavi/Alphavirus	Negative
6/20/2023	Malindi	MAL/S7/43654	M	25	*Ae.aegypti*	Flavi/Alphavirus	Negative
6/20/2023	Malindi	MAL/S7/43655	M	25	*Ae.aegypti*	Flavi/Alphavirus	Negative
6/20/2023	Malindi	MAL/S7/43656	F	25	*Cx.pipiens*	Flavi/Alphavirus	Negative
6/20/2023	Malindi	MAL/S7/43657	F	25	*Cx.pipiens*	Flavi/Alphavirus	Negative
6/20/2023	Malindi	MAL/S7/43658	F	25	*Cx.pipiens*	Flavi/Alphavirus	Negative

6/20/2023	Malindi	MAL/S7/43659	F	25	*Cx.pipiens*	Flavi/Alphavirus	Negative
6/20/2023	Malindi	MAL/S7/43660	F	10	*Cx.pipiens*	Flavi/Alphavirus	Negative
6/21/2023	Malindi	MAL/S8/43661	F	1	*Cx.univittatus*	Flavi/Alphavirus	Negative
6/21/2023	Malindi	MAL/S8/43662	F	4	*Ae.aegypti*	Flavi/Alphavirus	Negative
6/21/2023	Malindi	MAL/S8/43663	F	13	*Cx.pipiens*	Flavi/Alphavirus	Negative
6/21/2023	Malindi	MAL/S8/43664	F	1	*An.gambiae*	Flavi/Alphavirus	Negative
6/21/2023	Malindi	MAL/S8/43665	F	1	*Ae.aegypti*	Flavi/Alphavirus	Negative
6/21/2023	Malindi	MAL/S8/43666	M	1	*Ae.aegypti*	Flavi/Alphavirus	Negative
6/21/2023	Malindi	MAL/S8/43667	F	1	*Cx.annulioris*	Flavi/Alphavirus	Negative
6/21/2023	Malindi	MAL/S8/43668	F	13	*Cx.pipiens*	Flavi/Alphavirus	Negative
6/21/2023	Malindi	MAL/S8/43669	F	1	*Mn.uniformis*	Flavi/Alphavirus	Negative
6/21/2023	Malindi	MAL/S8/43670	F	4	*Cx.pipiens*	Flavi/Alphavirus	Negative
6/21/2023	Malindi	MAL/S8/43671	M	6	*Ae.aegypti*	Flavi/Alphavirus	Negative
6/21/2023	Malindi	MAL/S8/43672	F	25	*Ae.aegypti*	Flavi/Alphavirus	Negative
6/21/2023	Malindi	MAL/S8/43673	F	1	*Cx.annulioris*	Flavi/Alphavirus	Negative
6/21/2023	Malindi	MAL/S8/43674	M	1	*Ae.aegypti*	Flavi/Alphavirus	Negative
6/21/2023	Malindi	MAL/S8/43675	F	6	*Ae.aegypti*	Flavi/Alphavirus	Negative
6/21/2023	Malindi	MAL/S8/43676	F	18	*Cx.pipiens*	Flavi/Alphavirus	Negative
6/21/2023	Malindi	MAL/S8/43677	F	1	*Ae.aegypti*	Flavi/Alphavirus	Negative
6/21/2023	Malindi	MAL/S8/43678	M	1	*Ae.aegypti*	Flavi/Alphavirus	Negative
6/21/2023	Malindi	MAL/S8/43679	F	1	*Cx.annulioris*	Flavi/Alphavirus	Negative
6/21/2023	Malindi	MAL/S8/43680	F	16	*Cx.pipiens*	Flavi/Alphavirus	Negative
6/21/2023	Malindi	MAL/S8/43681	F	1	*Ae.pembaensis*	Flavi/Alphavirus	Negative
6/21/2023	Malindi	MAL/S8/43682	F	1	*Cx.annulioris*	Flavi/Alphavirus	Negative
6/21/2023	Malindi	MAL/S8/43683	F	2	*Cx.univittatus*	Flavi/Alphavirus	Negative
6/21/2023	Malindi	MAL/S8/43684	F	1	*Ae.aegypti*	Flavi/Alphavirus	Negative
6/21/2023	Malindi	MAL/S8/43685	M	1	*Ae.aegypti*	Flavi/Alphavirus	Negative
6/21/2023	Malindi	MAL/S8/43686	F	1	*Ae.circumluteolus*	Flavi/Alphavirus	Negative
6/21/2023	Malindi	MAL/S8/43687	F	23	*Cx.pipiens*	Flavi/Alphavirus	Negative
6/21/2023	Malindi	MAL/S8/43688	F	2	*Cx.annulioris*	Flavi/Alphavirus	Negative
6/21/2023	Malindi	MAL/S8/43689	F	4	*Cx.univittatus*	Flavi/Alphavirus	Negative
6/21/2023	Malindi	MAL/S8/43690	F	14	*Ae.aegypti*	Flavi/Alphavirus	Negative
6/21/2023	Malindi	MAL/S8/43691	F	25	*Cx.pipiens*	Flavi/Alphavirus	Negative
6/21/2023	Malindi	MAL/S8/43692	F	3	*Cx.pipiens*	Flavi/Alphavirus	Negative
6/21/2023	Malindi	MAL/S8/43693	F	1	*An.gambiae*	Flavi/Alphavirus	Negative
6/21/2023	Malindi	MAL/S8/43694	F	1	*Ae.circumluteolus*	Flavi/Alphavirus	Negative
6/21/2023	Malindi	MAL/S8/43695	F	11	*Cx.annulioris*	Flavi/Alphavirus	Negative
6/21/2023	Malindi	MAL/S8/43696	F	25	*Cx.pipiens*	Flavi/Alphavirus	Negative
6/21/2023	Malindi	MAL/S8/43697	F	25	*Cx.pipiens*	Flavi/Alphavirus	Negative
6/21/2023	Malindi	MAL/S8/43698	F	25	*Cx.pipiens*	Flavi/Alphavirus	Negative
6/22/2023	Malindi	MAL/S3/43699	F	8	*Cx.pipiens*	Flavi/Alphavirus	Negative
6/22/2023	Malindi	MAL/S3/43700	F	1	*Ae.pembaensis*	Flavi/Alphavirus	Negative
6/22/2023	Malindi	MAL/S3/43701	F	2	*Cx.annulioris*	Flavi/Alphavirus	Negative
6/22/2023	Malindi	MAL/S3/43702	F	1	*Cx.univittatus*	Flavi/Alphavirus	Negative
6/22/2023	Malindi	MAL/S3/43703	F	1	*Cx.pipiens*	Flavi/Alphavirus	Negative
6/22/2023	Malindi	MAL/S3/43704	F	2	*Cx.pipiens*	Flavi/Alphavirus	Negative
6/22/2023	Malindi	MAL/S3/43705	F	1	*Ae.aegypti*	Flavi/Alphavirus	Negative
6/22/2023	Malindi	MAL/S3/43706	F	1	*Cx.annulioris*	Flavi/Alphavirus	Negative
6/22/2023	Malindi	MAL/S3/43707	F	12	*Cx.pipiens*	Flavi/Alphavirus	Negative
6/22/2023	Malindi	MAL/S3/43708	F	1	*An.gambiae*	Flavi/Alphavirus	Negative

6/22/2023	Malindi	MAL/S3/43709	F	6	*Cx.univittatus*	Flavi/Alphavirus	Negative
6/22/2023	Malindi	MAL/S3/43710	F	6	*Cx.univittatus*	Flavi/Alphavirus	Negative
6/22/2023	Malindi	MAL/S3/43711	F	20	*Cx.pipiens*	Flavi/Alphavirus	Negative
6/22/2023	Malindi	MAL/S3/43712	F	2	*Cx.annulioris*	Flavi/Alphavirus	Negative
6/22/2023	Malindi	MAL/S3/43713	F	1	*Ae.circumluteolus*	Flavi/Alphavirus	Negative
6/22/2023	Malindi	MAL/S3/43714	F	8	*Ae.aegypti*	Flavi/Alphavirus	Negative
6/22/2023	Malindi	MAL/S3/43715	F	6	*Ae.aegypti*	Flavi/Alphavirus	Negative
6/22/2023	Malindi	MAL/S3/43716	M	1	*Ae.aegypti*	Flavi/Alphavirus	Negative
6/22/2023	Malindi	MAL/S3/43717	F	2	*Ae.simpsoni*	Flavi/Alphavirus	Negative
6/22/2023	Malindi	MAL/S3/43718	F	1	*Cx.rubinotus*	Flavi/Alphavirus	Negative
6/22/2023	Malindi	MAL/S3/43719	F	25	*Cx.univittatus*	Flavi/Alphavirus	Negative
6/22/2023	Malindi	MAL/S3/43720	F	3	*Cx.annulioris*	Flavi/Alphavirus	Negative
6/22/2023	Malindi	MAL/S3/43721	F	1	*Mn.uniformis*	Flavi/Alphavirus	Negative
6/22/2023	Malindi	MAL/S3/43722	F	14	*Cx.pipiens*	Flavi/Alphavirus	Negative
6/22/2023	Malindi	MAL/S3/43723	F	2	*An.gambiae*	Flavi/Alphavirus	Negative
6/22/2023	Malindi	MAL/S3/43724	F	2	*Cx.annulioris*	Flavi/Alphavirus	Negative
6/22/2023	Malindi	MAL/S3/43725	F	1	*Ae.simpsoni*	Flavi/Alphavirus	Negative
6/22/2023	Malindi	MAL/S3/43726	F	1	*Ae.aegypti*	Flavi/Alphavirus	Negative
6/22/2023	Malindi	MAL/S3/43727	F	19	*Cx.univittatus*	Flavi/Alphavirus	Negative
6/22/2023	Malindi	MAL/S3/43728	F	25	*Cx.pipiens*	Flavi/Alphavirus	Negative
6/22/2023	Malindi	MAL/S3/43729	F	17	*Cx.pipiens*	Flavi/Alphavirus	Negative
6/22/2023	Malindi	MAL/S3/43730	F	1	*Cx.univittatus*	Flavi/Alphavirus	Negative
6/22/2023	Malindi	MAL/S3/43731	F	6	*Cx.pipiens*	Flavi/Alphavirus	Negative
10/21/2023	Malindi	MAL/S2/43732	F	25	*Ae.pembaensis*	Flavi/Alphavirus	Negative
10/21/2023	Malindi	MAL/S2/43733	F	25	*Ae.pembaensis*	Flavi/Alphavirus	Negative
10/22/2023	Malindi	MAL/S5/43977	F	25	*Ae.pembaensis*	Flavi/Alphavirus	Negative
10/22/2023	Malindi	MAL/S5/43978	F	25	*Ae.pembaensis*	Flavi/Alphavirus	Negative
10/22/2023	Malindi	MAL/S5/43979	F	25	*Ae.pembaensis*	Flavi/Alphavirus	Negative
10/22/2023	Malindi	MAL/S5/43980	F	25	*Ae.pembaensis*	Flavi/Alphavirus	Negative
10/22/2023	Malindi	MAL/S5/43981	F	25	*Ae.pembaensis*	Flavi/Alphavirus	Negative
10/22/2023	Malindi	MAL/S5/43982	F	25	*Ae.pembaensis*	Flavi/Alphavirus	Negative
10/22/2023	Malindi	MAL/S5/43983	F	25	*Ae.pembaensis*	Flavi/Alphavirus	Negative
10/22/2023	Malindi	MAL/S5/43984	F	25	*Ae.pembaensis*	Flavi/Alphavirus	Negative
10/22/2023	Malindi	MAL/S5/43985	F	25	*Ae.pembaensis*	Flavi/Alphavirus	Negative
10/22/2023	Malindi	MAL/S5/43986	F	25	*Ae.pembaensis*	Flavi/Alphavirus	Negative
10/22/2023	Malindi	MAL/S5/43987	F	25	*Ae.pembaensis*	Flavi/Alphavirus	Negative
10/22/2023	Malindi	MAL/S5/43988	F	25	*Ae.pembaensis*	Flavi/Alphavirus	Negative
10/22/2023	Malindi	MAL/S5/43989	F	25	*Ae.pembaensis*	Flavi/Alphavirus	Negative
10/22/2023	Malindi	MAL/S5/43990	F	25	*Ae.pembaensis*	Flavi/Alphavirus	Negative
10/22/2023	Malindi	MAL/S5/43991	F	19	*Ae.pembaensis*	Flavi/Alphavirus	Negative
10/23/2023	Malindi	MAL/S3/43992	F	1	*Ae.metallicus*	Flavi/Alphavirus	Negative
10/23/2023	Malindi	MAL/S3/43993	F	6	*Cx.annulioris*	Flavi/Alphavirus	Negative
10/23/2023	Malindi	MAL/S3/43994	F	20	*Cx.pipiens*	Flavi/Alphavirus	Negative
10/23/2023	Malindi	MAL/S3/43995	F	25	*Cx.univittatus*	Flavi/Alphavirus	Negative
10/23/2023	Malindi	MAL/S3/43996	F	25	*Cx.univittatus*	Flavi/Alphavirus	Negative
10/23/2023	Malindi	MAL/S3/43997	F	20	*Cx.univittatus*	Flavi/Alphavirus	Negative
10/23/2023	Malindi	MAL/S3/43998	F	2	*Ae.pembaensis*	Flavi/Alphavirus	Negative
10/23/2023	Malindi	MAL/S3/43999	F	2	*Cx.annulioris*	Flavi/Alphavirus	Negative
10/23/2023	Malindi	MAL/S3/44000	F	8	*Cx.univittatus*	Flavi/Alphavirus	Negative
10/23/2023	Malindi	MAL/S3/44001	F	6	*Cx.pipiens*	Flavi/Alphavirus	Negative

10/23/2023	Malindi	MAL/S3/44002	F	25	*Ae.pembaensis*	Flavi/Alphavirus	Negative
10/23/2023	Malindi	MAL/S3/44003	F	7	*Ae.pembaensis*	Flavi/Alphavirus	Negative
10/23/2023	Malindi	MAL/S3/44004	F	3	*Cx.annulioris*	Flavi/Alphavirus	Negative
10/23/2023	Malindi	MAL/S3/44005	F	1	*Ae.aegypti*	Flavi/Alphavirus	Negative
10/23/2023	Malindi	MAL/S3/44006	F	10	*Cx.univittatus*	Flavi/Alphavirus	Negative
10/23/2023	Malindi	MAL/S3/44007	F	2	*Cx.pipiens*	Flavi/Alphavirus	Negative
10/23/2023	Malindi	MAL/S3/44008	F	1	*Cx.tigripes*	Flavi/Alphavirus	Negative
10/23/2023	Malindi	MAL/S3/44009	F	2	*Ae.aegypti*	Flavi/Alphavirus	Negative
10/23/2023	Malindi	MAL/S3/44010	F	3	*Cx.annulioris*	Flavi/Alphavirus	Negative
10/23/2023	Malindi	MAL/S3/44011	F	25	*Cx.pipiens*	Flavi/Alphavirus	Negative
10/23/2023	Malindi	MAL/S3/44012	F	5	*Cx.pipiens*	Flavi/Alphavirus	Negative
10/23/2023	Malindi	MAL/S3/44013	F	25	*Cx.univittatus*	Flavi/Alphavirus	Negative
10/23/2023	Malindi	MAL/S3/44014	F	25	*Cx.univittatus*	Flavi/Alphavirus	Negative
10/23/2023	Malindi	MAL/S3/44015	F	25	*Cx.univittatus*	Flavi/Alphavirus	Negative
10/23/2023	Malindi	MAL/S3/44016	F	25	*Cx.univittatus*	Flavi/Alphavirus	Negative
10/23/2023	Malindi	MAL/S3/44017	F	5	*Cx.univittatus*	Flavi/Alphavirus	Negative
10/23/2023	Malindi	MAL/S3/44018	F	2	*Cx.pipiens*	Flavi/Alphavirus	Negative
10/23/2023	Malindi	MAL/S3/44019	F	1	*Ae.tricholabis*	Flavi/Alphavirus	Negative
10/23/2023	Malindi	MAL/S3/44020	F	1	*Ae.circumluteolus*	Flavi/Alphavirus	Negative
10/23/2023	Malindi	MAL/S3/44021	F	11	*Cx.univittatus*	Flavi/Alphavirus	Negative
10/23/2023	Malindi	MAL/S3/44022	F	1	*Ae.tricholabis*	Flavi/Alphavirus	Negative
10/23/2023	Malindi	MAL/S3/44023	F	1	*Cx.pipiens*	Flavi/Alphavirus	Negative
10/23/2023	Malindi	MAL/S3/44024	F	2	*Ae.aegypti*	Flavi/Alphavirus	Negative
10/23/2023	Malindi	MAL/S3/44025	F	1	*Cx.annulioris*	Flavi/Alphavirus	Negative
10/23/2023	Malindi	MAL/S3/44026	F	17	*Cx.univittatus*	Flavi/Alphavirus	Negative
10/23/2023	Malindi	MAL/S3/44027	F	1	*Cx.rubinotus*	Flavi/Alphavirus	Negative
10/23/2023	Malindi	MAL/S3/44028	F	1	*Cx.annulioris*	Flavi/Alphavirus	Negative
10/23/2023	Malindi	MAL/S3/44029	F	1	*Ae.aegypti*	Flavi/Alphavirus	Negative
10/23/2023	Malindi	MAL/S3/44030	F	8	*Cx.pipiens*	Flavi/Alphavirus	Negative
10/23/2023	Malindi	MAL/S3/44031	F	25	*Cx.univittatus*	Flavi/Alphavirus	Negative
10/23/2023	Malindi	MAL/S3/44032	F	11	*Cx.univittatus*	Flavi/Alphavirus	Negative
10/24/2023	Malindi	MAL/S7/44033	M	1	*Ae.simpsoni*	Flavi/Alphavirus	Negative
10/24/2023	Malindi	MAL/S7/44034	F	7	*Ae.simpsoni*	Flavi/Alphavirus	Negative
10/24/2023	Malindi	MAL/S7/44035	F	1	*Ae.aegypti*	Flavi/Alphavirus	Negative
10/24/2023	Malindi	MAL/S7/44036	F	18	*Cx.pipiens*	Flavi/Alphavirus	Negative
10/24/2023	Malindi	MAL/S7/44037	F	20	*Cx.annulioris*	Flavi/Alphavirus	Negative
10/24/2023	Malindi	MAL/S7/44038	M	4	*Ae.aegypti*	Flavi/Alphavirus	Negative
10/24/2023	Malindi	MAL/S7/44039	F	7	*Ae.aegypti*	Flavi/Alphavirus	Negative
10/24/2023	Malindi	MAL/S7/44040	F	4	*Ae.simpsoni*	Flavi/Alphavirus	Negative
10/24/2023	Malindi	MAL/S7/44041	F	7	*Ae.pembaensis*	Flavi/Alphavirus	Negative
10/24/2023	Malindi	MAL/S7/44042	F	15	*Cx.annulioris*	Flavi/Alphavirus	Negative
10/24/2023	Malindi	MAL/S7/44043	F	14	*Cx.pipiens*	Flavi/Alphavirus	Negative
10/24/2023	Malindi	MAL/S7/44044	F	5	*Cx.univittatus*	Flavi/Alphavirus	Negative
10/24/2023	Malindi	MAL/S7/44045	F	2	*Ae.aegypti*	Flavi/Alphavirus	Negative
10/24/2023	Malindi	MAL/S7/44046	M	1	*Ae.aegypti*	Flavi/Alphavirus	Negative
10/24/2023	Malindi	MAL/S7/44047	F	7	*Cx.pipiens*	Flavi/Alphavirus	Negative
10/24/2023	Malindi	MAL/S7/44048	F	3	*Cx.univittatus*	Flavi/Alphavirus	Negative
10/24/2023	Malindi	MAL/S7/44049	F	5	*Cx.annulioris*	Flavi/Alphavirus	Negative
10/24/2023	Malindi	MAL/S7/44050	F	5	*Cx.univittatus*	Flavi/Alphavirus	Negative
10/24/2023	Malindi	MAL/S7/44051	F	1	*Ae.simpsoni*	Flavi/Alphavirus	Negative

10/24/2023	Malindi	MAL/S7/44052	M	8	*Ae.aegypti*	Flavi/Alphavirus	Negative
10/24/2023	Malindi	MAL/S7/44053	F	16	*Ae.aegypti*	Flavi/Alphavirus	Negative
10/24/2023	Malindi	MAL/S7/44054	F	17	*Cx.pipiens*	Flavi/Alphavirus	Negative
10/24/2023	Malindi	MAL/S7/44055	F	15	*Cx.annulioris*	Flavi/Alphavirus	Negative
10/24/2023	Malindi	MAL/S7/44056	F	8	*Cx.pipiens*	Flavi/Alphavirus	Negative
10/24/2023	Malindi	MAL/S7/44057	F	6	*Cx.annulioris*	Flavi/Alphavirus	Negative
10/24/2023	Malindi	MAL/S7/44058	F	1	*Cx.rubinotus*	Flavi/Alphavirus	Negative
10/24/2023	Malindi	MAL/S7/44059	F	19	*Ae.aegypti*	Flavi/Alphavirus	Negative
10/24/2023	Malindi	MAL/S7/44060	M	6	*Ae.aegypti*	Flavi/Alphavirus	Negative
10/24/2023	Malindi	MAL/S7/44061	F	16	*Cx.annulioris*	Flavi/Alphavirus	Negative
10/24/2023	Malindi	MAL/S7/44062	F	22	*Cx.pipiens*	Flavi/Alphavirus	Negative
10/24/2023	Malindi	MAL/S7/44063	F	4	*Ae.simpsoni*	Flavi/Alphavirus	Negative
10/24/2023	Malindi	MAL/S7/44064	M	1	*Ae.simpsoni*	Flavi/Alphavirus	Negative
10/24/2023	Malindi	MAL/S7/44065	F	17	*Cx.univittatus*	Flavi/Alphavirus	Negative
10/21/2023	Malindi	MAL/S3/44066	F	15	*Cx.pipiens*	Flavi/Alphavirus	Positive - WNV
10/21/2023	Malindi	MAL/S3/44067	F	7	*Ae.aegypti*	Flavi/Alphavirus	Negative
10/21/2023	Malindi	MAL/S3/44068	M	14	*Ae.aegypti*	Flavi/Alphavirus	Negative
12/7/2023	Malindi	MAL/S5/44069	F	25	*Cx.annulioris*	Flavi/Alphavirus	Negative
12/7/2023	Malindi	MAL/S5/44070	F	25	*Cx.annulioris*	Flavi/Alphavirus	Negative
12/7/2023	Malindi	MAL/S5/44071	F	25	*Cx.annulioris*	Flavi/Alphavirus	Negative
12/7/2023	Malindi	MAL/S5/44072	F	25	*Cx.annulioris*	Flavi/Alphavirus	Negative
12/7/2023	Malindi	MAL/S5/44073	F	25	*Cx.annulioris*	Flavi/Alphavirus	Negative
12/7/2023	Malindi	MAL/S5/44074	F	25	*Cx.annulioris*	Flavi/Alphavirus	Negative
12/7/2023	Malindi	MAL/S5/44075	F	25	*Cx.annulioris*	Flavi/Alphavirus	Negative
12/7/2023	Malindi	MAL/S5/44076	F	25	*Cx.annulioris*	Flavi/Alphavirus	Negative
12/7/2023	Malindi	MAL/S5/44077	F	24	*Cx.annulioris*	Flavi/Alphavirus	Negative
12/7/2023	Malindi	MAL/S5/44078	F	2	*An.coustani*	Flavi/Alphavirus	Negative
12/7/2023	Malindi	MAL/S5/44079	F	6	*Cx.pipiens*	Flavi/Alphavirus	Negative
12/7/2023	Malindi	MAL/S5/44080	F	1	*Cx.tigripes*	Flavi/Alphavirus	Negative
12/7/2023	Malindi	MAL/S5/44081	F	9	*Cx.duttoni*	Flavi/Alphavirus	Negative
12/7/2023	Malindi	MAL/S5/44082	F	4	*Ae.circumluteolus*	Flavi/Alphavirus	Negative
12/7/2023	Malindi	MAL/S5/44083	F	4	*Ae.pembaensis*	Flavi/Alphavirus	Negative
12/7/2023	Malindi	MAL/S5/44084	F	8	*Mn.uniformis*	Flavi/Alphavirus	Negative
12/7/2023	Malindi	MAL/S5/44085	F	2	*An.gambiae*	Flavi/Alphavirus	Negative
12/7/2023	Malindi	MAL/S5/44086	F	25	*Cx.annulioris*	Flavi/Alphavirus	Negative
12/7/2023	Malindi	MAL/S5/44087	F	25	*Cx.annulioris*	Flavi/Alphavirus	Negative
12/7/2023	Malindi	MAL/S5/44088	F	25	*Cx.annulioris*	Flavi/Alphavirus	Negative
12/7/2023	Malindi	MAL/S5/44089	F	25	*Cx.annulioris*	Flavi/Alphavirus	Negative
12/7/2023	Malindi	MAL/S5/44090	F	25	*Cx.annulioris*	Flavi/Alphavirus	Negative
12/7/2023	Malindi	MAL/S5/44091	F	25	*Cx.annulioris*	Flavi/Alphavirus	Negative
12/7/2023	Malindi	MAL/S5/44092	F	7	*Ae.quasiunivittatus*	Flavi/Alphavirus	Negative
12/7/2023	Malindi	MAL/S5/44093	F	12	*An.coustani*	Flavi/Alphavirus	Negative
12/7/2023	Malindi	MAL/S5/44094	F	4	*Ae.sudanensis*	Flavi/Alphavirus	Negative
12/7/2023	Malindi	MAL/S5/44095	F	7	*Cx.duttoni*	Flavi/Alphavirus	Negative
12/7/2023	Malindi	MAL/S5/44096	F	23	*Mn.uniformis*	Flavi/Alphavirus	Negative
12/7/2023	Malindi	MAL/S5/44097	F	10	*Cx.univittatus*	Flavi/Alphavirus	Negative
12/7/2023	Malindi	MAL/S5/44098	F	1	*An.gambiae*	Flavi/Alphavirus	Negative
12/7/2023	Malindi	MAL/S5/44099	F	22	*Ae.pembaensis*	Flavi/Alphavirus	Negative
12/7/2023	Malindi	MAL/S5/44100	F	3	*Cx.pipiens*	Flavi/Alphavirus	Negative
12/7/2023	Malindi	MAL/S5/44101	F	5	*Ae.circumluteolus*	Flavi/Alphavirus	Negative

12/7/2023	Malindi	MAL/S5/44102	F	1	*Cx.bitaeniorhynchus*	Flavi/Alphavirus	Negative
12/7/2023	Malindi	MAL/S5/44103	F	1	*An.pharoensis*	Flavi/Alphavirus	Negative
12/7/2023	Malindi	MAL/S5/44104	F	1	*Ae.metallicus*	Flavi/Alphavirus	Negative
12/8/2023	Malindi	MAL/S7/44105	F	1	*Mn.uniformis*	Flavi/Alphavirus	Negative
12/8/2023	Malindi	MAL/S7/44106	F	14	*Cx.pipiens*	Flavi/Alphavirus	Negative
12/8/2023	Malindi	MAL/S7/44107	M	1	*Ae.aegypti*	Flavi/Alphavirus	Negative
12/8/2023	Malindi	MAL/S7/44108	F	1	*Cx.annulioris*	Flavi/Alphavirus	Negative
12/8/2023	Malindi	MAL/S7/44109	F	11	*Ae.aegypti*	Flavi/Alphavirus	Negative
12/8/2023	Malindi	MAL/S7/44110	F	12	*Cx.univittatus*	Flavi/Alphavirus	Negative
12/8/2023	Malindi	MAL/S7/44111	F	17	*Cx.pipiens*	Flavi/Alphavirus	Negative
12/8/2023	Malindi	MAL/S7/44112	F	1	*Cx.annulioris*	Flavi/Alphavirus	Negative
12/8/2023	Malindi	MAL/S7/44113	F	3	*Ae.aegypti*	Flavi/Alphavirus	Negative
12/8/2023	Malindi	MAL/S7/44114	F	6	*Cx.univittatus*	Flavi/Alphavirus	Negative
12/8/2023	Malindi	MAL/S7/44115	F	1	*Cx.rubinotus*	Flavi/Alphavirus	Negative
12/8/2023	Malindi	MAL/S7/44116	F	2	*Cx.univittatus*	Flavi/Alphavirus	Negative
12/8/2023	Malindi	MAL/S7/44117	F	1	*An.coustani*	Flavi/Alphavirus	Negative
12/8/2023	Malindi	MAL/S7/44118	F	8	*Ae.aegypti*	Flavi/Alphavirus	Negative
12/8/2023	Malindi	MAL/S7/44119	F	25	*Cx.pipiens*	Flavi/Alphavirus	Negative
12/8/2023	Malindi	MAL/S7/44120	F	7	*Cx.pipiens*	Flavi/Alphavirus	Negative
12/8/2023	Malindi	MAL/S7/44121	F	1	*Ae.aegypti*	Flavi/Alphavirus	Negative
12/8/2023	Malindi	MAL/S7/44122	M	4	*Ae.aegypti*	Flavi/Alphavirus	Negative
12/8/2023	Malindi	MAL/S7/44123	F	5	*Cx.univittatus*	Flavi/Alphavirus	Negative
12/8/2023	Malindi	MAL/S7/44124	F	25	*Cx.pipiens*	Flavi/Alphavirus	Negative
12/8/2023	Malindi	MAL/S7/44125	F	3	*Cx.pipiens*	Flavi/Alphavirus	Negative
12/8/2023	Malindi	MAL/S7/44126	F	1	*Ae.aegypti*	Flavi/Alphavirus	Negative
12/8/2023	Malindi	MAL/S7/44127	F	1	*Ae.simpsoni*	Flavi/Alphavirus	Negative
12/8/2023	Malindi	MAL/S7/44128	F	1	*Cx.univittatus*	Flavi/Alphavirus	Negative
12/8/2023	Malindi	MAL/S7/44129	F	7	*Cx.pipiens*	Flavi/Alphavirus	Negative
12/8/2023	Malindi	MAL/S7/44130	F	1	*Cx.rubinotus*	Flavi/Alphavirus	Negative
12/8/2023	Malindi	MAL/S7/44131	F	1	*An.coustani*	Flavi/Alphavirus	Negative
12/8/2023	Malindi	MAL/S7/44132	F	7	*Cx.univittatus*	Flavi/Alphavirus	Negative
12/8/2023	Malindi	MAL/S7/44133	F	25	*Cx.pipiens*	Flavi/Alphavirus	Negative
12/8/2023	Malindi	MAL/S7/44134	F	2	*Cx.pipiens*	Flavi/Alphavirus	Negative
12/8/2023	Malindi	MAL/S7/44135	F	1	*Cx.rubinotus*	Flavi/Alphavirus	Negative
12/8/2023	Malindi	MAL/S7/44136	F	1	*Cx.annulioris*	Flavi/Alphavirus	Negative
12/8/2023	Malindi	MAL/S7/44137	F	17	*Cx.pipiens*	Flavi/Alphavirus	Negative
12/8/2023	Malindi	MAL/S7/44138	F	23	*Cx.univittatus*	Flavi/Alphavirus	Negative
12/9/2023	Malindi	MAL/S2/44220	F	25	*Ae.pembaensis*	Flavi/Alphavirus	Negative
12/9/2023	Malindi	MAL/S2/44221	F	25	*Ae.pembaensis*	Flavi/Alphavirus	Negative
12/9/2023	Malindi	MAL/S2/44222	F	25	*Ae.pembaensis*	Flavi/Alphavirus	Negative
12/9/2023	Malindi	MAL/S2/44223	F	25	*Ae.pembaensis*	Flavi/Alphavirus	Negative
12/9/2023	Malindi	MAL/S2/44224	F	25	*Ae.pembaensis*	Flavi/Alphavirus	Negative
12/9/2023	Malindi	MAL/S2/44225	F	25	*Ae.pembaensis*	Flavi/Alphavirus	Negative
12/9/2023	Malindi	MAL/S2/44226	F	25	*Ae.pembaensis*	Flavi/Alphavirus	Negative
12/9/2023	Malindi	MAL/S2/44227	F	25	*Ae.pembaensis*	Flavi/Alphavirus	Negative
12/9/2023	Malindi	MAL/S2/44228	F	23	*Ae.pembaensis*	Flavi/Alphavirus	Negative
12/10/2023	Malindi	MAL/S9/44229	F	11	*Ae.quasiunivittatus*	Flavi/Alphavirus	Negative
12/10/2023	Malindi	MAL/S9/44230	F	2	*An.pharoensis*	Flavi/Alphavirus	Negative
12/10/2023	Malindi	MAL/S9/44231	F	2	*An.squamosus*	Flavi/Alphavirus	Negative
12/10/2023	Malindi	MAL/S9/44232	F	15	*Mn.uniformis*	Flavi/Alphavirus	Negative

12/10/2023	Malindi	MAL/S9/44233	F	13	*Cx.univittatus*	Flavi/Alphavirus	Negative
12/10/2023	Malindi	MAL/S9/44234	F	17	*Cx.pipiens*	Flavi/Alphavirus	Negative
12/10/2023	Malindi	MAL/S9/44235	F	17	*An.funestus*	Flavi/Alphavirus	Positive - WNV
12/10/2023	Malindi	MAL/S9/44236	F	1	*Ae.circumluteolus*	Flavi/Alphavirus	Negative
12/10/2023	Malindi	MAL/S9/44237	F	3	*Ae.simpsoni*	Flavi/Alphavirus	Negative
12/10/2023	Malindi	MAL/S9/44238	F	1	*Cx.rubinotus*	Flavi/Alphavirus	Negative
12/10/2023	Malindi	MAL/S9/44239	F	25	*Cx.annulioris*	Flavi/Alphavirus	Negative
12/10/2023	Malindi	MAL/S9/44240	F	25	*Cx.annulioris*	Flavi/Alphavirus	Negative
12/10/2023	Malindi	MAL/S9/44241	F	25	*Cx.annulioris*	Flavi/Alphavirus	Negative
12/10/2023	Malindi	MAL/S9/44242	F	25	*Cx.annulioris*	Flavi/Alphavirus	Negative
12/10/2023	Malindi	MAL/S9/44243	F	25	*Cx.annulioris*	Flavi/Alphavirus	Negative
12/10/2023	Malindi	MAL/S9/44244	F	25	*Cx.annulioris*	Flavi/Alphavirus	Negative
12/10/2023	Malindi	MAL/S9/44245	F	25	*Cx.annulioris*	Flavi/Alphavirus	Negative
12/10/2023	Malindi	MAL/S9/44246	F	25	*Cx.annulioris*	Flavi/Alphavirus	Negative
12/10/2023	Malindi	MAL/S9/44247	F	25	*Cx.annulioris*	Flavi/Alphavirus	Negative
12/10/2023	Malindi	MAL/S9/44248	F	25	*Cx.annulioris*	Flavi/Alphavirus	Negative
12/10/2023	Malindi	MAL/S9/44249	F	25	*Cx.annulioris*	Flavi/Alphavirus	Negative
12/10/2023	Malindi	MAL/S9/44250	F	25	*Cx.annulioris*	Flavi/Alphavirus	Negative
12/10/2023	Malindi	MAL/S9/44251	F	25	*Cx.annulioris*	Flavi/Alphavirus	Negative
12/10/2023	Malindi	MAL/S9/44252	F	25	*Cx.annulioris*	Flavi/Alphavirus	Negative
12/10/2023	Malindi	MAL/S9/44253	F	11	*Cx.annulioris*	Flavi/Alphavirus	Negative
12/10/2023	Malindi	MAL/S9/44254	F	1	*Ae.sudanensis*	Flavi/Alphavirus	Negative
12/10/2023	Malindi	MAL/S9/44255	F	1	*Ae.hirsutus*	Flavi/Alphavirus	Negative
12/10/2023	Malindi	MAL/S9/44256	F	1	*Ae.simpsoni*	Flavi/Alphavirus	Negative
12/10/2023	Malindi	MAL/S9/44257	F	3	*Ae.aegypti*	Flavi/Alphavirus	Negative
12/10/2023	Malindi	MAL/S9/44258	F	9	*An.funestus*	Flavi/Alphavirus	Negative
12/10/2023	Malindi	MAL/S9/44259	F	20	*Ae.circumluteolus*	Flavi/Alphavirus	Negative
12/10/2023	Malindi	MAL/S9/44260	F	17	*Cx.univittatus*	Flavi/Alphavirus	Negative
12/10/2023	Malindi	MAL/S9/44261	F	21	*Cx.pipiens*	Flavi/Alphavirus	Negative
12/10/2023	Malindi	MAL/S9/44262	F	10	*Ae.quasiunivittatus*	Flavi/Alphavirus	Negative
12/10/2023	Malindi	MAL/S9/44263	F	10	*Mn.uniformis*	Flavi/Alphavirus	Negative
12/10/2023	Malindi	MAL/S9/44264	F	5	*An.coustani*	Flavi/Alphavirus	Negative
12/10/2023	Malindi	MAL/S9/44265	F	7	*An.gambiae*	Flavi/Alphavirus	Negative
12/10/2023	Malindi	MAL/S9/44266	F	25	*Cx.annulioris*	Flavi/Alphavirus	Negative
12/10/2023	Malindi	MAL/S9/44267	F	25	*Cx.annulioris*	Flavi/Alphavirus	Negative
12/10/2023	Malindi	MAL/S9/44268	F	25	*Cx.annulioris*	Flavi/Alphavirus	Negative
12/10/2023	Malindi	MAL/S9/44269	F	25	*Cx.annulioris*	Flavi/Alphavirus	Negative
12/10/2023	Malindi	MAL/S9/44270	F	25	*Cx.annulioris*	Flavi/Alphavirus	Negative
12/10/2023	Malindi	MAL/S9/44271	F	25	*Cx.annulioris*	Flavi/Alphavirus	Negative
12/10/2023	Malindi	MAL/S9/44272	F	25	*Cx.annulioris*	Flavi/Alphavirus	Negative
12/10/2023	Malindi	MAL/S9/44273	F	18	*Cx.annulioris*	Flavi/Alphavirus	Negative
12/12/2023	Mombasa	MBSA/S1/44274	F	1	*Ae.metallicus*	Flavi/Alphavirus	Negative
12/12/2023	Mombasa	MBSA/S1/44275	F	3	*Ae.aegypti*	Flavi/Alphavirus	Negative
12/12/2023	Mombasa	MBSA/S1/44276	M	3	*Ae.aegypti*	Flavi/Alphavirus	Negative
12/12/2023	Mombasa	MBSA/S1/44277	F	1	*An.gambiae*	Flavi/Alphavirus	Negative
12/12/2023	Mombasa	MBSA/S1/44278	F	2	*Cx.univittatus*	Flavi/Alphavirus	Negative
12/12/2023	Mombasa	MBSA/S1/44279	F	1	*Cx.pipiens*	Flavi/Alphavirus	Negative
12/12/2023	Mombasa	MBSA/S1/44280	F	1	*An.coustani*	Flavi/Alphavirus	Negative
12/12/2023	Mombasa	MBSA/S1/44281	F	3	*Ae.metallicus*	Flavi/Alphavirus	Negative
12/12/2023	Mombasa	MBSA/S1/44282	F	11	*Ae.aegypti*	Flavi/Alphavirus	Negative

12/12/2023	Mombasa	MBSA/S1/44283	M	1	*Ae.aegypti*	Flavi/Alphavirus	Negative
12/12/2023	Mombasa	MBSA/S1/44284	F	4	*Ae.vittatus*	Flavi/Alphavirus	Negative
12/12/2023	Mombasa	MBSA/S1/44285	M	2	*Ae.vittatus*	Flavi/Alphavirus	Negative
12/12/2023	Mombasa	MBSA/S1/44286	F	3	*Cx.annulioris*	Flavi/Alphavirus	Negative
12/12/2023	Mombasa	MBSA/S1/44287	F	7	*Cx.pipiens*	Flavi/Alphavirus	Negative
12/12/2023	Mombasa	MBSA/S1/44288	F	13	*Cx.univittatus*	Flavi/Alphavirus	Positive - WNV
12/12/2023	Mombasa	MBSA/S1/44289	F	1	*Ae.metallicus*	Flavi/Alphavirus	Negative
12/12/2023	Mombasa	MBSA/S1/44290	F	1	*An.funestus*	Flavi/Alphavirus	Negative
12/12/2023	Mombasa	MBSA/S1/44291	M	1	*Ae.aegypti*	Flavi/Alphavirus	Negative
12/12/2023	Mombasa	MBSA/S1/44292	F	7	*Ae.aegypti*	Flavi/Alphavirus	Negative
12/12/2023	Mombasa	MBSA/S1/44293	F	3	*Cx.annulioris*	Flavi/Alphavirus	Negative
12/12/2023	Mombasa	MBSA/S1/44294	F	4	*Ae.vittatus*	Flavi/Alphavirus	Negative
12/12/2023	Mombasa	MBSA/S1/44295	F	1	*Mn.uniformis*	Flavi/Alphavirus	Negative
12/12/2023	Mombasa	MBSA/S1/44296	F	7	*Cx.univittatus*	Flavi/Alphavirus	Negative
12/12/2023	Mombasa	MBSA/S1/44297	F	12	*Cx.pipiens*	Flavi/Alphavirus	Negative
12/12/2023	Mombasa	MBSA/S1/44298	F	2	*An.coustani*	Flavi/Alphavirus	Negative
12/12/2023	Mombasa	MBSA/S1/44299	F	3	*Cx.pipiens*	Flavi/Alphavirus	Negative
12/12/2023	Mombasa	MBSA/S1/44300	F	8	*Cx.annulioris*	Flavi/Alphavirus	Negative
12/12/2023	Mombasa	MBSA/S1/44301	F	3	*Ae.dentatus*	Flavi/Alphavirus	Negative
12/12/2023	Mombasa	MBSA/S1/44302	F	15	*Cx.univittatus*	Flavi/Alphavirus	Negative
12/12/2023	Mombasa	MBSA/S1/44303	M	1	*Ae.aegypti*	Flavi/Alphavirus	Negative
12/12/2023	Mombasa	MBSA/S1/44304	F	7	*Ae.aegypti*	Flavi/Alphavirus	Negative
12/12/2023	Mombasa	MBSA/S1/44305	F	21	*Ae.vittatus*	Flavi/Alphavirus	Negative
12/12/2023	Mombasa	MBSA/S1/44306	M	4	*Ae.vittatus*	Flavi/Alphavirus	Negative
12/12/2023	Mombasa	MBSA/S1/44307	F	2	*Ae.metallicus*	Flavi/Alphavirus	Negative
12/12/2023	Mombasa	MBSA/S1/44308	F	1	*Ae.circumluteolus*	Flavi/Alphavirus	Negative
12/12/2023	Mombasa	MBSA/S1/44309	F	1	*Uranotaenia spps*	Flavi/Alphavirus	Negative
12/12/2023	Mombasa	MBSA/S1/44310	F	1	*An.gambiae*	Flavi/Alphavirus	Negative
12/12/2023	Mombasa	MBSA/S1/44311	F	6	*Cx.pipiens*	Flavi/Alphavirus	Negative
12/12/2023	Mombasa	MBSA/S1/44312	F	1	*Eret.chryogaster*	Flavi/Alphavirus	Negative
12/12/2023	Mombasa	MBSA/S1/44313	F	8	*Cx.annulioris*	Flavi/Alphavirus	Negative
12/12/2023	Mombasa	MBSA/S1/44314	F	6	*Cx.univittatus*	Flavi/Alphavirus	Negative
12/12/2023	Mombasa	MBSA/S1/44315	M	1	*Ae.vittatus*	Flavi/Alphavirus	Negative
12/12/2023	Mombasa	MBSA/S1/44316	F	7	*Ae.vittatus*	Flavi/Alphavirus	Negative
12/12/2023	Mombasa	MBSA/S1/44317	M	13	*Ae.aegypti*	Flavi/Alphavirus	Negative
12/12/2023	Mombasa	MBSA/S1/44318	F	25	*Ae.aegypti*	Flavi/Alphavirus	Negative
12/12/2023	Mombasa	MBSA/S1/44319	F	4	*Ae.aegypti*	Flavi/Alphavirus	Negative
12/12/2023	Mombasa	MBSA/S1/44320	F	1	*Uranotaenia spps*	Flavi/Alphavirus	Negative
12/12/2023	Mombasa	MBSA/S1/44321	F	1	*Ae.dentatus*	Flavi/Alphavirus	Negative
12/12/2023	Mombasa	MBSA/S1/44322	F	1	*Ae.circumluteolus*	Flavi/Alphavirus	Negative
12/12/2023	Mombasa	MBSA/S1/44323	F	3	*Ae.metallicus*	Flavi/Alphavirus	Negative
12/12/2023	Mombasa	MBSA/S1/44324	F	1	*An.pharoensis*	Flavi/Alphavirus	Negative
12/12/2023	Mombasa	MBSA/S1/44325	F	16	*Cx.pipiens*	Flavi/Alphavirus	Negative
12/12/2023	Mombasa	MBSA/S1/44326	F	5	*Cx.univittatus*	Flavi/Alphavirus	Negative
12/12/2023	Mombasa	MBSA/S1/44327	F	12	*Cx.annulioris*	Flavi/Alphavirus	Negative
12/12/2023	Mombasa	MBSA/S1/44328	M	2	*Ae.aegypti*	Flavi/Alphavirus	Negative
12/12/2023	Mombasa	MBSA/S1/44329	F	5	*Ae.aegypti*	Flavi/Alphavirus	Negative
12/12/2023	Mombasa	MBSA/S1/44330	M	2	*Ae.vittatus*	Flavi/Alphavirus	Negative
12/12/2023	Mombasa	MBSA/S1/44331	F	25	*Ae.vittatus*	Flavi/Alphavirus	Negative
12/12/2023	Mombasa	MBSA/S1/44332	F	2	*Mn.uniformis*	Flavi/Alphavirus	Negative

12/12/2023	Mombasa	MBSA/S1/44333	F	1	*Ae.circumluteolus*	Flavi/Alphavirus	Negative
12/12/2023	Mombasa	MBSA/S1/44334	F	1	*An.funestus*	Flavi/Alphavirus	Negative
12/12/2023	Mombasa	MBSA/S1/44335	F	5	*Cx.annulioris*	Flavi/Alphavirus	Negative
12/12/2023	Mombasa	MBSA/S1/44336	F	9	*Cx.univittatus*	Flavi/Alphavirus	Negative
12/12/2023	Mombasa	MBSA/S1/44337	F	7	*Cx.pipiens*	Flavi/Alphavirus	Negative
12/12/2023	Mombasa	MBSA/S1/44338	M	2	*Ae.vittatus*	Flavi/Alphavirus	Negative
12/12/2023	Mombasa	MBSA/S1/44339	F	12	*Ae.vittatus*	Flavi/Alphavirus	Negative
12/12/2023	Mombasa	MBSA/S1/44340	M	2	*Ae.aegypti*	Flavi/Alphavirus	Negative
12/12/2023	Mombasa	MBSA/S1/44341	F	15	*Ae.aegypti*	Flavi/Alphavirus	Negative
12/12/2023	Mombasa	MBSA/S1/44342	F	1	*An.coustani*	Flavi/Alphavirus	Negative
12/12/2023	Mombasa	MBSA/S1/44343	F	1	*Ae.circumluteolus*	Flavi/Alphavirus	Negative
12/12/2023	Mombasa	MBSA/S1/44344	F	1	*Ae.metallicus*	Flavi/Alphavirus	Negative
12/12/2023	Mombasa	MBSA/S1/44345	F	2	*Ae.dentatus*	Flavi/Alphavirus	Negative
12/12/2023	Mombasa	MBSA/S1/44346	F	1	*Cx.univittatus*	Flavi/Alphavirus	Negative
12/12/2023	Mombasa	MBSA/S1/44347	F	8	*Cx.pipiens*	Flavi/Alphavirus	Negative
12/12/2023	Mombasa	MBSA/S1/44348	F	7	*Cx.annulioris*	Flavi/Alphavirus	Negative
12/12/2023	Mombasa	MBSA/S1/44349	M	1	*Ae.aegypti*	Flavi/Alphavirus	Negative
12/12/2023	Mombasa	MBSA/S1/44350	F	14	*Ae.aegypti*	Flavi/Alphavirus	Negative
12/12/2023	Mombasa	MBSA/S1/44351	M	3	*Ae.vittatus*	Flavi/Alphavirus	Negative
12/12/2023	Mombasa	MBSA/S1/44352	F	10	*Ae.vittatus*	Flavi/Alphavirus	Negative
12/13/2023	Mombasa	MBSA/S2/44353	F	1	*Eret.chryogaster*	Flavi/Alphavirus	Negative
12/13/2023	Mombasa	MBSA/S2/44354	M	2	*Eret.chryogaster*	Flavi/Alphavirus	Negative
12/13/2023	Mombasa	MBSA/S2/44355	F	6	*Cx.annulioris*	Flavi/Alphavirus	Negative
12/13/2023	Mombasa	MBSA/S2/44356	F	9	*Cx.univittatus*	Flavi/Alphavirus	Negative
12/13/2023	Mombasa	MBSA/S2/44357	F	12	*Cx.pipiens*	Flavi/Alphavirus	Negative
12/13/2023	Mombasa	MBSA/S2/44358	M	7	*Ae.aegypti*	Flavi/Alphavirus	Positive - WNV
12/13/2023	Mombasa	MBSA/S2/44359	F	6	*Ae.aegypti*	Flavi/Alphavirus	Negative
12/13/2023	Mombasa	MBSA/S2/44360	F	25	*Ae.vittatus*	Flavi/Alphavirus	Negative
12/13/2023	Mombasa	MBSA/S2/44361	F	9	*Ae.vittatus*	Flavi/Alphavirus	Negative
12/13/2023	Mombasa	MBSA/S2/44362	M	21	*Ae.vittatus*	Flavi/Alphavirus	Negative
12/13/2023	Mombasa	MBSA/S2/44363	F	3	*Ae.simpsoni*	Flavi/Alphavirus	Negative
12/13/2023	Mombasa	MBSA/S2/44364	F	2	*Ae.quasiunivittatus*	Flavi/Alphavirus	Negative
12/13/2023	Mombasa	MBSA/S2/44365	F	7	*Cx.annulioris*	Flavi/Alphavirus	Negative
12/13/2023	Mombasa	MBSA/S2/44366	F	13	*Cx.univittatus*	Flavi/Alphavirus	Negative
12/13/2023	Mombasa	MBSA/S2/44367	F	4	*Cx.pipiens*	Flavi/Alphavirus	Negative
12/13/2023	Mombasa	MBSA/S2/44368	M	24	*Ae.aegypti*	Flavi/Alphavirus	Negative
12/13/2023	Mombasa	MBSA/S2/44369	F	17	*Ae.aegypti*	Flavi/Alphavirus	Negative
12/13/2023	Mombasa	MBSA/S2/44370	M	1	*Ae.vittatus*	Flavi/Alphavirus	Negative
12/13/2023	Mombasa	MBSA/S2/44371	F	10	*Ae.vittatus*	Flavi/Alphavirus	Negative
12/13/2023	Mombasa	MBSA/S2/44372	F	2	*Ae.simpsoni*	Flavi/Alphavirus	Negative
12/13/2023	Mombasa	MBSA/S2/44373	F	1	*An.pharoensis*	Flavi/Alphavirus	Negative
12/13/2023	Mombasa	MBSA/S2/44374	F	3	*Ae.quasiunivittatus*	Flavi/Alphavirus	Negative
12/13/2023	Mombasa	MBSA/S2/44375	M	1	*Eret.chryogaster*	Flavi/Alphavirus	Negative
12/13/2023	Mombasa	MBSA/S2/44376	F	5	*Eret.chryogaster*	Flavi/Alphavirus	Negative
12/13/2023	Mombasa	MBSA/S2/44377	F	8	*Cx.annulioris*	Flavi/Alphavirus	Negative
12/13/2023	Mombasa	MBSA/S2/44378	F	16	*Cx.univittatus*	Flavi/Alphavirus	Negative
12/13/2023	Mombasa	MBSA/S2/44379	F	20	*Cx.pipiens*	Flavi/Alphavirus	Negative
12/13/2023	Mombasa	MBSA/S2/44380	M	9	*Ae.aegypti*	Flavi/Alphavirus	Negative
12/13/2023	Mombasa	MBSA/S2/44381	F	12	*Ae.aegypti*	Flavi/Alphavirus	Negative
12/13/2023	Mombasa	MBSA/S2/44382	M	19	*Ae.vittatus*	Flavi/Alphavirus	Negative

12/13/2023	Mombasa	MBSA/S2/44383	F	25	*Ae.vittatus*	Flavi/Alphavirus	Negative
12/13/2023	Mombasa	MBSA/S2/44384	F	25	*Ae.vittatus*	Flavi/Alphavirus	Negative
12/13/2023	Mombasa	MBSA/S2/44385	F	25	*Ae.vittatus*	Flavi/Alphavirus	Negative
12/13/2023	Mombasa	MBSA/S2/44386	F	13	*Ae.vittatus*	Flavi/Alphavirus	Negative
12/13/2023	Mombasa	MBSA/S2/44387	F	1	*Ae.circumluteolus*	Flavi/Alphavirus	Negative
12/13/2023	Mombasa	MBSA/S2/44388	F	1	*Ae.simpsoni*	Flavi/Alphavirus	Negative
12/13/2023	Mombasa	MBSA/S2/44389	F	1	*Cx.tigripes*	Flavi/Alphavirus	Negative
12/13/2023	Mombasa	MBSA/S2/44390	F	2	*Eret.chryogaster*	Flavi/Alphavirus	Negative
12/13/2023	Mombasa	MBSA/S2/44391	F	1	*An.coustani*	Flavi/Alphavirus	Negative
12/13/2023	Mombasa	MBSA/S2/44392	F	5	*Cx.annulioris*	Flavi/Alphavirus	Negative
12/13/2023	Mombasa	MBSA/S2/44393	F	13	*Cx.univittatus*	Flavi/Alphavirus	Negative
12/13/2023	Mombasa	MBSA/S2/44394	F	2	*Eret.chryogaster*	Flavi/Alphavirus	Negative
12/13/2023	Mombasa	MBSA/S2/44395	F	2	*Ae.simpsoni*	Flavi/Alphavirus	Negative
12/13/2023	Mombasa	MBSA/S2/44396	F	7	*Cx.pipiens*	Flavi/Alphavirus	Negative
12/13/2023	Mombasa	MBSA/S2/44397	M	2	*Ae.aegypti*	Flavi/Alphavirus	Negative
12/13/2023	Mombasa	MBSA/S2/44398	F	11	*Ae.aegypti*	Flavi/Alphavirus	Negative
12/13/2023	Mombasa	MBSA/S2/44399	M	25	*Ae.vittatus*	Flavi/Alphavirus	Negative
12/13/2023	Mombasa	MBSA/S2/44400	M	25	*Ae.vittatus*	Flavi/Alphavirus	Negative
12/13/2023	Mombasa	MBSA/S2/44401	M	6	*Ae.vittatus*	Flavi/Alphavirus	Negative
12/13/2023	Mombasa	MBSA/S2/44402	F	25	*Ae.vittatus*	Flavi/Alphavirus	Negative
12/13/2023	Mombasa	MBSA/S2/44403	F	26	*Ae.vittatus*	Flavi/Alphavirus	Negative
12/13/2023	Mombasa	MBSA/S2/44404	F	3	*Cx.annulioris*	Flavi/Alphavirus	Negative
12/13/2023	Mombasa	MBSA/S2/44405	F	5	*Cx.univittatus*	Flavi/Alphavirus	Negative
12/13/2023	Mombasa	MBSA/S2/44406	F	11	*Cx.pipiens*	Flavi/Alphavirus	Negative
12/13/2023	Mombasa	MBSA/S2/44407	M	24	*Ae.aegypti*	Flavi/Alphavirus	Negative
12/13/2023	Mombasa	MBSA/S2/44408	F	25	*Ae.aegypti*	Flavi/Alphavirus	Negative
12/13/2023	Mombasa	MBSA/S2/44409	F	19	*Ae.aegypti*	Flavi/Alphavirus	Negative
12/13/2023	Mombasa	MBSA/S2/44410	M	14	*Ae.vittatus*	Flavi/Alphavirus	Negative
12/13/2023	Mombasa	MBSA/S2/44411	F	21	*Ae.vittatus*	Flavi/Alphavirus	Negative
12/13/2023	Mombasa	MBSA/S2/44412	F	2	*Eret.chryogaster*	Flavi/Alphavirus	Negative
12/13/2023	Mombasa	MBSA/S2/44413	F	1	*Cx.tigripes*	Flavi/Alphavirus	Negative
12/13/2023	Mombasa	MBSA/S2/44414	F	3	*Ae.simpsoni*	Flavi/Alphavirus	Negative
12/13/2023	Mombasa	MBSA/S2/44415	F	1	*Ae.quasiunivittatus*	Flavi/Alphavirus	Negative
12/13/2023	Mombasa	MBSA/S2/44416	F	8	*Cx.annulioris*	Flavi/Alphavirus	Negative
12/13/2023	Mombasa	MBSA/S2/44417	F	19	*Cx.univittatus*	Flavi/Alphavirus	Negative
12/13/2023	Mombasa	MBSA/S2/44418	F	22	*Cx.pipiens*	Flavi/Alphavirus	Negative
12/13/2023	Mombasa	MBSA/S2/44419	M	6	*Ae.aegypti*	Flavi/Alphavirus	Negative
12/13/2023	Mombasa	MBSA/S2/44420	F	24	*Ae.aegypti*	Flavi/Alphavirus	Negative
12/13/2023	Mombasa	MBSA/S2/44421	M	2	*Ae.vittatus*	Flavi/Alphavirus	Negative
12/13/2023	Mombasa	MBSA/S2/44422	F	10	*Ae.vittatus*	Flavi/Alphavirus	Negative
12/13/2023	Mombasa	MBSA/S2/44423	F	1	*An.gambiae*	Flavi/Alphavirus	Negative
12/13/2023	Mombasa	MBSA/S2/44424	F	4	*Eret.chryogaster*	Flavi/Alphavirus	Negative
12/13/2023	Mombasa	MBSA/S2/44425	F	1	*Mn.africana*	Flavi/Alphavirus	Negative
12/13/2023	Mombasa	MBSA/S2/44426	F	1	*Ae.sudanensis*	Flavi/Alphavirus	Negative
12/13/2023	Mombasa	MBSA/S2/44427	F	2	*Ae.quasiunivittatus*	Flavi/Alphavirus	Negative
12/13/2023	Mombasa	MBSA/S2/44428	F	3	*Ae.simpsoni*	Flavi/Alphavirus	Negative
12/13/2023	Mombasa	MBSA/S2/44429	F	1	*Ae.circumluteolus*	Flavi/Alphavirus	Negative
12/13/2023	Mombasa	MBSA/S2/44430	F	1	*Cx.tigripes*	Flavi/Alphavirus	Negative
12/13/2023	Mombasa	MBSA/S2/44431	F	16	*Cx.annulioris*	Flavi/Alphavirus	Negative
12/13/2023	Mombasa	MBSA/S2/44432	F	11	*Cx.univittatus*	Flavi/Alphavirus	Negative

12/13/2023	Mombasa	MBSA/S2/44433	F	12	*Cx.pipiens*	Flavi/Alphavirus	Negative
12/13/2023	Mombasa	MBSA/S2/44434	M	6	*Ae.aegypti*	Flavi/Alphavirus	Negative
12/13/2023	Mombasa	MBSA/S2/44435	F	20	*Ae.aegypti*	Flavi/Alphavirus	Negative
12/13/2023	Mombasa	MBSA/S2/44436	M	7	*Ae.vittatus*	Flavi/Alphavirus	Negative
12/13/2023	Mombasa	MBSA/S2/44437	F	11	*Ae.vittatus*	Flavi/Alphavirus	Negative
12/13/2023	Mombasa	MBSA/S2/44438	M	4	*Eret.chryogaster*	Flavi/Alphavirus	Negative
12/13/2023	Mombasa	MBSA/S2/44439	F	4	*Eret.chryogaster*	Flavi/Alphavirus	Positive - WNV
12/13/2023	Mombasa	MBSA/S2/44440	F	4	*Ae.quasiunivittatus*	Flavi/Alphavirus	Negative
12/13/2023	Mombasa	MBSA/S2/44441	F	1	*Ae.circumluteolus*	Flavi/Alphavirus	Negative
12/13/2023	Mombasa	MBSA/S2/44442	F	10	*Cx.annulioris*	Flavi/Alphavirus	Negative
12/13/2023	Mombasa	MBSA/S2/44443	F	27	*Cx.univittatus*	Flavi/Alphavirus	Negative
12/13/2023	Mombasa	MBSA/S2/44444	F	13	*Ae.aegypti*	Flavi/Alphavirus	Negative
12/13/2023	Mombasa	MBSA/S2/44445	M	25	*Ae.aegypti*	Flavi/Alphavirus	Negative
12/13/2023	Mombasa	MBSA/S2/44446	M	16	*Ae.aegypti*	Flavi/Alphavirus	Negative
12/13/2023	Mombasa	MBSA/S2/44447	M	25	*Ae.vittatus*	Flavi/Alphavirus	Negative
12/13/2023	Mombasa	MBSA/S2/44448	M	25	*Ae.vittatus*	Flavi/Alphavirus	Negative
12/13/2023	Mombasa	MBSA/S2/44449	M	24	*Ae.vittatus*	Flavi/Alphavirus	Negative
12/13/2023	Mombasa	MBSA/S2/44450	F	25	*Ae.vittatus*	Flavi/Alphavirus	Negative
12/13/2023	Mombasa	MBSA/S2/44451	F	25	*Ae.vittatus*	Flavi/Alphavirus	Negative
12/13/2023	Mombasa	MBSA/S2/44452	F	25	*Ae.vittatus*	Flavi/Alphavirus	Negative
12/13/2023	Mombasa	MBSA/S2/44453	F	9	*Ae.vittatus*	Flavi/Alphavirus	Negative
12/14/2023	Mombasa	MBSA/S3/44454	F	12	*Ae.simpsoni*	Flavi/Alphavirus	Negative
12/14/2023	Mombasa	MBSA/S3/44455	F	1	*Ae.pembaensis*	Flavi/Alphavirus	Negative
12/14/2023	Mombasa	MBSA/S3/44456	F	1	*Ae.circumluteolus*	Flavi/Alphavirus	Negative
12/14/2023	Mombasa	MBSA/S3/44457	F	19	*Cx.annulioris*	Flavi/Alphavirus	Negative
12/14/2023	Mombasa	MBSA/S3/44458	F	23	*Cx.univittatus*	Flavi/Alphavirus	Negative
12/14/2023	Mombasa	MBSA/S3/44459	M	6	*Ae.aegypti*	Flavi/Alphavirus	Negative
12/14/2023	Mombasa	MBSA/S3/44460	F	11	*Ae.aegypti*	Flavi/Alphavirus	Negative
12/14/2023	Mombasa	MBSA/S3/44461	M	1	*Ae.vittatus*	Flavi/Alphavirus	Negative
12/14/2023	Mombasa	MBSA/S3/44462	F	16	*Ae.vittatus*	Flavi/Alphavirus	Negative
12/14/2023	Mombasa	MBSA/S3/44463	F	1	*Cx.annulioris*	Flavi/Alphavirus	Negative
12/14/2023	Mombasa	MBSA/S3/44464	F	2	*Cx.univittatus*	Flavi/Alphavirus	Negative
12/14/2023	Mombasa	MBSA/S3/44465	M	1	*Ae.aegypti*	Flavi/Alphavirus	Negative
12/14/2023	Mombasa	MBSA/S3/44466	F	3	*Ae.aegypti*	Flavi/Alphavirus	Negative
12/14/2023	Mombasa	MBSA/S3/44467	M	1	*Ae.vittatus*	Flavi/Alphavirus	Negative
12/14/2023	Mombasa	MBSA/S3/44468	F	1	*Ae.vittatus*	Flavi/Alphavirus	Negative
12/14/2023	Mombasa	MBSA/S3/44469	F	1	*An.funestus*	Flavi/Alphavirus	Negative
12/14/2023	Mombasa	MBSA/S3/44470	F	9	*Cx.annulioris*	Flavi/Alphavirus	Negative
12/14/2023	Mombasa	MBSA/S3/44471	F	25	*Cx.univittatus*	Flavi/Alphavirus	Negative
12/14/2023	Mombasa	MBSA/S3/44472	M	5	*Ae.aegypti*	Flavi/Alphavirus	Negative
12/14/2023	Mombasa	MBSA/S3/44473	F	2	*Ae.aegypti*	Flavi/Alphavirus	Negative
12/14/2023	Mombasa	MBSA/S3/44474	F	2	*Ae.vittatus*	Flavi/Alphavirus	Negative
12/14/2023	Mombasa	MBSA/S3/44475	F	2	*Ae.simpsoni*	Flavi/Alphavirus	Negative
12/14/2023	Mombasa	MBSA/S3/44476	F	1	*Mn.uniformis*	Flavi/Alphavirus	Negative
12/14/2023	Mombasa	MBSA/S3/44477	F	1	*Ae.simpsoni*	Flavi/Alphavirus	Negative
12/14/2023	Mombasa	MBSA/S3/44478	F	2	*Cx.annulioris*	Flavi/Alphavirus	Negative
12/14/2023	Mombasa	MBSA/S3/44479	F	6	*Cx.univittatus*	Flavi/Alphavirus	Negative
12/14/2023	Mombasa	MBSA/S3/44480	M	4	*Ae.aegypti*	Flavi/Alphavirus	Negative
12/14/2023	Mombasa	MBSA/S3/44481	F	2	*Ae.aegypti*	Flavi/Alphavirus	Negative
12/14/2023	Mombasa	MBSA/S3/44482	M	1	*Ae.vittatus*	Flavi/Alphavirus	Negative

12/14/2023	Mombasa	MBSA/S3/44483	F	3	*Ae.vittatus*	Flavi/Alphavirus	Negative
12/14/2023	Mombasa	MBSA/S3/44484	F	3	*Cx.tigripes*	Flavi/Alphavirus	Negative
12/14/2023	Mombasa	MBSA/S3/44485	F	1	*Eret.chryogaster*	Flavi/Alphavirus	Negative
12/14/2023	Mombasa	MBSA/S3/44486	F	1	*Ae.simpsoni*	Flavi/Alphavirus	Negative
12/14/2023	Mombasa	MBSA/S3/44487	F	1	*Uranotaenia spps*	Flavi/Alphavirus	Negative
12/14/2023	Mombasa	MBSA/S3/44488	F	3	*Cx.annulioris*	Flavi/Alphavirus	Negative
12/14/2023	Mombasa	MBSA/S3/44489	F	5	*Cx.univittatus*	Flavi/Alphavirus	Negative
12/14/2023	Mombasa	MBSA/S3/44490	M	5	*Ae.aegypti*	Flavi/Alphavirus	Negative
12/14/2023	Mombasa	MBSA/S3/44491	F	11	*Ae.aegypti*	Flavi/Alphavirus	Positive - WNV
12/14/2023	Mombasa	MBSA/S3/44492	M	1	*Ae.vittatus*	Flavi/Alphavirus	Negative
12/14/2023	Mombasa	MBSA/S3/44493	F	16	*Ae.vittatus*	Flavi/Alphavirus	Negative
12/14/2023	Mombasa	MBSA/S3/44494	F	7	*Cx.annulioris*	Flavi/Alphavirus	Negative
12/14/2023	Mombasa	MBSA/S3/44495	M	1	*Ae.aegypti*	Flavi/Alphavirus	Negative
12/14/2023	Mombasa	MBSA/S3/44496	F	6	*Ae.aegypti*	Flavi/Alphavirus	Positive - WNV
12/14/2023	Mombasa	MBSA/S3/44497	F	4	*Ae.vittatus*	Flavi/Alphavirus	Negative
12/14/2023	Mombasa	MBSA/S3/44498	F	2	*Ae.simpsoni*	Flavi/Alphavirus	Negative
12/14/2023	Mombasa	MBSA/S3/44499	F	1	*Eret.chryogaster*	Flavi/Alphavirus	Negative
12/14/2023	Mombasa	MBSA/S3/44500	F	1	*Ae.quasiunivittatus*	Flavi/Alphavirus	Negative
12/14/2023	Mombasa	MBSA/S3/44501	F	1	*Cx.annulioris*	Flavi/Alphavirus	Negative
12/14/2023	Mombasa	MBSA/S3/44502	F	1	*Cx.univittatus*	Flavi/Alphavirus	Negative
12/14/2023	Mombasa	MBSA/S3/44503	M	3	*Ae.aegypti*	Flavi/Alphavirus	Negative
12/14/2023	Mombasa	MBSA/S3/44504	F	2	*Ae.aegypti*	Flavi/Alphavirus	Negative
12/14/2023	Mombasa	MBSA/S3/44505	F	1	*Mn.uniformis*	Flavi/Alphavirus	Negative
12/14/2023	Mombasa	MBSA/S3/44506	F	1	*An.coustani*	Flavi/Alphavirus	Negative
12/14/2023	Mombasa	MBSA/S3/44507	F	1	*Ae.simpsoni*	Flavi/Alphavirus	Negative
12/14/2023	Mombasa	MBSA/S3/44508	F	1	*Ae.pembaensis*	Flavi/Alphavirus	Negative
12/14/2023	Mombasa	MBSA/S3/44509	F	7	*Cx.annulioris*	Flavi/Alphavirus	Negative
12/14/2023	Mombasa	MBSA/S3/44510	F	1	*Cx.univittatus*	Flavi/Alphavirus	Negative
12/14/2023	Mombasa	MBSA/S3/44511	M	2	*Ae.aegypti*	Flavi/Alphavirus	Negative
12/14/2023	Mombasa	MBSA/S3/44512	F	6	*Ae.aegypti*	Flavi/Alphavirus	Negative
12/14/2023	Mombasa	MBSA/S3/44513	F	1	*Eret.chryogaster*	Flavi/Alphavirus	Negative
12/14/2023	Mombasa	MBSA/S3/44514	F	1	*Cx.tigripes*	Flavi/Alphavirus	Negative
12/14/2023	Mombasa	MBSA/S3/44515	F	1	*Ae.vittatus*	Flavi/Alphavirus	Negative
12/14/2023	Mombasa	MBSA/S3/44516	F	3	*Cx.univittatus*	Flavi/Alphavirus	Negative
12/14/2023	Mombasa	MBSA/S3/44517	F	8	*Ae.aegypti*	Flavi/Alphavirus	Negative
12/14/2023	Mombasa	MBSA/S3/44518	M	25	*Ae.aegypti*	Flavi/Alphavirus	Negative
12/14/2023	Mombasa	MBSA/S3/44519	M	24	*Ae.aegypti*	Flavi/Alphavirus	Negative
12/16/2023	Kwale	KWL/S4/44520	F	10	*Mn.uniformis*	Flavi/Alphavirus	Negative
12/16/2023	Kwale	KWL/S4/44521	F	1	*An.coustani*	Flavi/Alphavirus	Negative
12/16/2023	Kwale	KWL/S4/44522	F	1	*Coq.metallicus*	Flavi/Alphavirus	Negative
12/16/2023	Kwale	KWL/S4/44523	F	1	*An.gambiae*	Flavi/Alphavirus	Negative
12/16/2023	Kwale	KWL/S4/44524	F	3	*An.funestus*	Flavi/Alphavirus	Negative
12/16/2023	Kwale	KWL/S4/44525	F	1	*Cx.univittatus*	Flavi/Alphavirus	Negative
12/16/2023	Kwale	KWL/S4/44526	F	2	*Ae.aegypti*	Flavi/Alphavirus	Negative
12/16/2023	Kwale	KWL/S4/44527	F	3	*Ae.simpsoni*	Flavi/Alphavirus	Negative
12/16/2023	Kwale	KWL/S4/44528	F	10	*Cx.pipiens*	Flavi/Alphavirus	Negative
12/16/2023	Kwale	KWL/S4/44529	M	1	*Ae.aegypti*	Flavi/Alphavirus	Negative
12/16/2023	Kwale	KWL/S4/44530	F	7	*Ae.pembaensis*	Flavi/Alphavirus	Negative
12/16/2023	Kwale	KWL/S4/44531	F	23	*Cx.annulioris*	Flavi/Alphavirus	Negative
12/16/2023	Kwale	KWL/S4/44532	F	1	*Aedeomyia furfurea*	Flavi/Alphavirus	Negative

12/16/2023	Kwale	KWL/S4/44533	F	1	*Ae.simpsoni*	Flavi/Alphavirus	Negative
12/16/2023	Kwale	KWL/S4/44534	F	1	*Ae.aegypti*	Flavi/Alphavirus	Negative
12/16/2023	Kwale	KWL/S4/44535	F	1	*Coq.aurites*	Flavi/Alphavirus	Negative
12/16/2023	Kwale	KWL/S4/44536	F	3	*Ae.quasiunivittatus*	Flavi/Alphavirus	Negative
12/16/2023	Kwale	KWL/S4/44537	F	2	*Cx.cinereus*	Flavi/Alphavirus	Negative
12/16/2023	Kwale	KWL/S4/44538	F	1	*An.funestus*	Flavi/Alphavirus	Negative
12/16/2023	Kwale	KWL/S4/44539	F	5	*Cx.univittatus*	Flavi/Alphavirus	Negative
12/16/2023	Kwale	KWL/S4/44540	F	6	*Ae.pembaensis*	Flavi/Alphavirus	Negative
12/16/2023	Kwale	KWL/S4/44541	F	10	*Cx.pipiens*	Flavi/Alphavirus	Negative
12/16/2023	Kwale	KWL/S4/44542	F	23	*Mn.uniformis*	Flavi/Alphavirus	Negative
12/16/2023	Kwale	KWL/S4/44543	F	18	*Cx.annulioris*	Flavi/Alphavirus	Negative
12/16/2023	Kwale	KWL/S4/44544	F	7	*Ae.pembaensis*	Flavi/Alphavirus	Negative
12/16/2023	Kwale	KWL/S4/44545	F	1	*An.pharoensis*	Flavi/Alphavirus	Negative
12/16/2023	Kwale	KWL/S4/44546	F	1	*Coq.metallicus*	Flavi/Alphavirus	Negative
12/16/2023	Kwale	KWL/S4/44547	F	5	*Cx.univittatus*	Flavi/Alphavirus	Negative
12/16/2023	Kwale	KWL/S4/44548	F	13	*Cx.pipiens*	Flavi/Alphavirus	Negative
12/16/2023	Kwale	KWL/S4/44549	F	25	*Cx.annulioris*	Flavi/Alphavirus	Negative
12/16/2023	Kwale	KWL/S4/44550	F	7	*Cx.annulioris*	Flavi/Alphavirus	Negative
12/16/2023	Kwale	KWL/S4/44551	F	17	*Mn.uniformis*	Flavi/Alphavirus	Negative
12/16/2023	Kwale	KWL/S4/44552	F	1	*Eret.chryogaster*	Flavi/Alphavirus	Negative
12/16/2023	Kwale	KWL/S4/44553	F	1	*Coq.aurites*	Flavi/Alphavirus	Negative
12/16/2023	Kwale	KWL/S4/44554	F	7	*An.funestus*	Flavi/Alphavirus	Negative
12/16/2023	Kwale	KWL/S4/44555	F	2	*Cx.cinereus*	Flavi/Alphavirus	Negative
12/16/2023	Kwale	KWL/S4/44556	F	2	*An.gambiae*	Flavi/Alphavirus	Negative
12/16/2023	Kwale	KWL/S4/44557	F	2	*An.coustani*	Flavi/Alphavirus	Negative
12/16/2023	Kwale	KWL/S4/44558	M	1	*Ae.aegypti*	Flavi/Alphavirus	Negative
12/16/2023	Kwale	KWL/S4/44559	F	1	*Ae.simpsoni*	Flavi/Alphavirus	Negative
12/16/2023	Kwale	KWL/S4/44560	F	9	*Cx.univittatus*	Flavi/Alphavirus	Negative
12/16/2023	Kwale	KWL/S4/44561	F	2	*Ae.aegypti*	Flavi/Alphavirus	Negative
12/16/2023	Kwale	KWL/S4/44562	M	1	*Ae.aegypti*	Flavi/Alphavirus	Negative
12/16/2023	Kwale	KWL/S4/44563	F	1	*Ae.circumluteolus*	Flavi/Alphavirus	Negative
12/16/2023	Kwale	KWL/S4/44564	F	2	*Ae.simpsoni*	Flavi/Alphavirus	Negative
12/16/2023	Kwale	KWL/S4/44565	F	3	*An.pharoensis*	Flavi/Alphavirus	Negative
12/16/2023	Kwale	KWL/S4/44566	F	11	*An.funestus*	Flavi/Alphavirus	Negative
12/16/2023	Kwale	KWL/S4/44567	F	3	*Mn.uniformis*	Flavi/Alphavirus	Negative
12/16/2023	Kwale	KWL/S4/44568	F	23	*Cx.annulioris*	Flavi/Alphavirus	Negative
12/16/2023	Kwale	KWL/S4/44569	F	24	*Cx.pipiens*	Flavi/Alphavirus	Negative
12/16/2023	Kwale	KWL/S4/44570	F	17	*Cx.univittatus*	Flavi/Alphavirus	Negative
12/16/2023	Kwale	KWL/S4/44571	F	14	*Ae.pembaensis*	Flavi/Alphavirus	Negative
12/16/2023	Kwale	KWL/S4/44572	F	18	*Cx.pipiens*	Flavi/Alphavirus	Negative
12/16/2023	Kwale	KWL/S4/44573	F	27	*Cx.annulioris*	Flavi/Alphavirus	Negative
12/16/2023	Kwale	KWL/S4/44574	F	3	*An.squamosus*	Flavi/Alphavirus	Negative
12/16/2023	Kwale	KWL/S4/44575	F	1	*Coq.aurites*	Flavi/Alphavirus	Negative
12/16/2023	Kwale	KWL/S4/44576	F	1	*Cx.cinereus*	Flavi/Alphavirus	Negative
12/16/2023	Kwale	KWL/S4/44577	F	3	*Ae.quasiunivittatus*	Flavi/Alphavirus	Negative
12/16/2023	Kwale	KWL/S4/44578	M	2	*Ae.aegypti*	Flavi/Alphavirus	Negative
12/16/2023	Kwale	KWL/S4/44579	F	3	*Ae.aegypti*	Flavi/Alphavirus	Negative
12/16/2023	Kwale	KWL/S4/44580	F	12	*An.funestus*	Flavi/Alphavirus	Negative
12/16/2023	Kwale	KWL/S4/44581	F	14	*Mn.uniformis*	Flavi/Alphavirus	Negative
12/16/2023	Kwale	KWL/S4/44582	F	3	*Ae.pembaensis*	Flavi/Alphavirus	Negative

12/16/2023	Kwale	KWL/S4/44583	F	4	*Cx.univittatus*	Flavi/Alphavirus	Negative
12/16/2023	Kwale	KWL/S4/44584	F	18	*Cx.annulioris*	Flavi/Alphavirus	Negative
12/16/2023	Kwale	KWL/S4/44585	F	24	*Cx.pipiens*	Flavi/Alphavirus	Negative
12/16/2023	Kwale	KWL/S4/44586	F	16	*Mn.uniformis*	Flavi/Alphavirus	Negative
12/16/2023	Kwale	KWL/S4/44587	F	1	*Uranotaenia spps*	Flavi/Alphavirus	Negative
12/16/2023	Kwale	KWL/S4/44588	F	1	*An.funestus*	Flavi/Alphavirus	Negative
12/16/2023	Kwale	KWL/S4/44589	F	2	*Coq.metallicus*	Flavi/Alphavirus	Negative
12/16/2023	Kwale	KWL/S4/44590	F	7	*Ae.aegypti*	Flavi/Alphavirus	Negative
12/16/2023	Kwale	KWL/S4/44591	F	3	*Ae.simpsoni*	Flavi/Alphavirus	Negative
12/16/2023	Kwale	KWL/S4/44592	F	1	*Ae.simpsoni*	Flavi/Alphavirus	Negative
12/16/2023	Kwale	KWL/S4/44593	M	2	*Ae.aegypti*	Flavi/Alphavirus	Negative
12/16/2023	Kwale	KWL/S4/44594	F	5	*Ae.aegypti*	Flavi/Alphavirus	Negative
12/16/2023	Kwale	KWL/S4/44595	F	1	*Coq.aurites*	Flavi/Alphavirus	Negative
12/16/2023	Kwale	KWL/S4/44596	F	5	*Coq.metallicus*	Flavi/Alphavirus	Negative
12/16/2023	Kwale	KWL/S4/44597	F	2	*Cx.cinereus*	Flavi/Alphavirus	Negative
12/16/2023	Kwale	KWL/S4/44598	F	5	*An.funestus*	Flavi/Alphavirus	Negative
12/16/2023	Kwale	KWL/S4/44599	F	1	*Aedeomyia furfurea*	Flavi/Alphavirus	Negative
12/16/2023	Kwale	KWL/S4/44600	F	1	*Cx.tigripes*	Flavi/Alphavirus	Negative
12/16/2023	Kwale	KWL/S4/44601	F	2	*Ae.pembaensis*	Flavi/Alphavirus	Negative
12/16/2023	Kwale	KWL/S4/44602	F	19	*Cx.univittatus*	Flavi/Alphavirus	Negative
12/16/2023	Kwale	KWL/S4/44603	F	12	*Cx.pipiens*	Flavi/Alphavirus	Negative
12/16/2023	Kwale	KWL/S4/44604	F	9	*Mn.uniformis*	Flavi/Alphavirus	Negative
12/16/2023	Kwale	KWL/S4/44605	F	25	*Cx.annulioris*	Flavi/Alphavirus	Negative
12/16/2023	Kwale	KWL/S4/44606	F	16	*Cx.annulioris*	Flavi/Alphavirus	Negative
12/17/2023	Kwale	KWL/S5/44607	F	2	*Cx.univittatus*	Flavi/Alphavirus	Negative
12/17/2023	Kwale	KWL/S5/44608	F	9	*Ae.pembaensis*	Flavi/Alphavirus	Negative
12/17/2023	Kwale	KWL/S5/44609	F	13	*Cx.pipiens*	Flavi/Alphavirus	Negative
12/17/2023	Kwale	KWL/S5/44610	F	11	*Mn.uniformis*	Flavi/Alphavirus	Negative
12/17/2023	Kwale	KWL/S5/44611	F	7	*Cx.annulioris*	Flavi/Alphavirus	Negative
12/17/2023	Kwale	KWL/S5/44612	F	3	*Ae.aegypti*	Flavi/Alphavirus	Negative
12/17/2023	Kwale	KWL/S5/44613	M	1	*Ae.aegypti*	Flavi/Alphavirus	Negative
12/17/2023	Kwale	KWL/S5/44614	F	6	*Ae.simpsoni*	Flavi/Alphavirus	Negative
12/17/2023	Kwale	KWL/S5/44615	F	13	*Cx.univittatus*	Flavi/Alphavirus	Negative
12/17/2023	Kwale	KWL/S5/44616	F	13	*Cx.annulioris*	Flavi/Alphavirus	Negative
12/17/2023	Kwale	KWL/S5/44617	F	21	*Ae.pembaensis*	Flavi/Alphavirus	Negative
12/17/2023	Kwale	KWL/S5/44618	F	12	*Mn.africana*	Flavi/Alphavirus	Negative
12/17/2023	Kwale	KWL/S5/44619	F	25	*Mn.uniformis*	Flavi/Alphavirus	Negative
12/17/2023	Kwale	KWL/S5/44620	F	25	*Mn.uniformis*	Flavi/Alphavirus	Negative
12/17/2023	Kwale	KWL/S5/44621	F	25	*Mn.uniformis*	Flavi/Alphavirus	Negative
12/17/2023	Kwale	KWL/S5/44622	F	25	*Mn.uniformis*	Flavi/Alphavirus	Negative
12/17/2023	Kwale	KWL/S5/44623	F	13	*Mn.uniformis*	Flavi/Alphavirus	Negative
12/17/2023	Kwale	KWL/S5/44624	F	6	*An.coustani*	Flavi/Alphavirus	Negative
12/17/2023	Kwale	KWL/S5/44625	F	4	*An.squamosus*	Flavi/Alphavirus	Negative
12/17/2023	Kwale	KWL/S5/44626	F	1	*Ae.circumluteolus*	Flavi/Alphavirus	Negative
12/17/2023	Kwale	KWL/S5/44627	F	1	*Cx.rubinotus*	Flavi/Alphavirus	Negative
12/17/2023	Kwale	KWL/S5/44628	F	2	*An.funestus*	Flavi/Alphavirus	Negative
12/17/2023	Kwale	KWL/S5/44629	F	1	*Coq.aurites*	Flavi/Alphavirus	Negative
12/17/2023	Kwale	KWL/S5/44630	F	1	*Coq.metallicus*	Flavi/Alphavirus	Negative
12/17/2023	Kwale	KWL/S5/44631	F	1	*Ae.simpsoni*	Flavi/Alphavirus	Negative
12/17/2023	Kwale	KWL/S5/44632	F	1	*Ae.aegypti*	Flavi/Alphavirus	Positive - WNV

12/17/2023	Kwale	KWL/S5/44633	F	1	*Ae.simpsoni*	Flavi/Alphavirus	Negative
12/17/2023	Kwale	KWL/S5/44634	F	1	*Cx.rubinotus*	Flavi/Alphavirus	Negative
12/17/2023	Kwale	KWL/S5/44635	F	1	*An.squamosus*	Flavi/Alphavirus	Negative
12/17/2023	Kwale	KWL/S5/44636	F	2	*Cx.cinereus*	Flavi/Alphavirus	Negative
12/17/2023	Kwale	KWL/S5/44637	F	1	*An.gambiae*	Flavi/Alphavirus	Negative
12/17/2023	Kwale	KWL/S5/44638	F	1	*An.funestus*	Flavi/Alphavirus	Negative
12/17/2023	Kwale	KWL/S5/44639	F	3	*Cx.univittatus*	Flavi/Alphavirus	Negative
12/17/2023	Kwale	KWL/S5/44640	F	2	*Ae.pembaensis*	Flavi/Alphavirus	Negative
12/17/2023	Kwale	KWL/S5/44641	F	10	*Cx.annulioris*	Flavi/Alphavirus	Negative
12/17/2023	Kwale	KWL/S5/44642	F	18	*Mn.uniformis*	Flavi/Alphavirus	Negative
12/17/2023	Kwale	KWL/S5/44643	F	3	*Cx.annulioris*	Flavi/Alphavirus	Negative
12/17/2023	Kwale	KWL/S5/44644	F	5	*Cx.univittatus*	Flavi/Alphavirus	Negative
12/17/2023	Kwale	KWL/S5/44645	F	11	*Ae.pembaensis*	Flavi/Alphavirus	Negative
12/17/2023	Kwale	KWL/S5/44646	F	2	*Mn.africana*	Flavi/Alphavirus	Negative
12/17/2023	Kwale	KWL/S5/44647	F	17	*Mn.uniformis*	Flavi/Alphavirus	Negative
12/17/2023	Kwale	KWL/S5/44648	F	1	*Coq.aurites*	Flavi/Alphavirus	Negative
12/17/2023	Kwale	KWL/S5/44649	F	2	*Ae.simpsoni*	Flavi/Alphavirus	Negative
12/17/2023	Kwale	KWL/S5/44650	F	1	*Ae.aegypti*	Flavi/Alphavirus	Negative
12/17/2023	Kwale	KWL/S5/44651	F	1	*An.squamosus*	Flavi/Alphavirus	Negative
12/17/2023	Kwale	KWL/S5/44652	F	2	*An.funestus*	Flavi/Alphavirus	Negative
12/17/2023	Kwale	KWL/S5/44653	F	1	*An.gambiae*	Flavi/Alphavirus	Negative
12/17/2023	Kwale	KWL/S5/44654	F	2	*An.coustani*	Flavi/Alphavirus	Negative
12/17/2023	Kwale	KWL/S5/44655	F	1	*Coq.metallicus*	Flavi/Alphavirus	Negative
12/17/2023	Kwale	KWL/S5/44656	F	1	*Ae.quasiunivittatus*	Flavi/Alphavirus	Negative
12/17/2023	Kwale	KWL/S5/44657	F	1	*An.coustani*	Flavi/Alphavirus	Negative
12/17/2023	Kwale	KWL/S5/44658	F	1	*An.squamosus*	Flavi/Alphavirus	Negative
12/17/2023	Kwale	KWL/S5/44659	F	2	*Ae.simpsoni*	Flavi/Alphavirus	Negative
12/17/2023	Kwale	KWL/S5/44660	M	1	*Ae.simpsoni*	Flavi/Alphavirus	Negative
12/17/2023	Kwale	KWL/S5/44661	F	3	*Cx.univittatus*	Flavi/Alphavirus	Negative
12/17/2023	Kwale	KWL/S5/44662	F	2	*An.funestus*	Flavi/Alphavirus	Negative
12/17/2023	Kwale	KWL/S5/44663	F	5	*Cx.annulioris*	Flavi/Alphavirus	Negative
12/17/2023	Kwale	KWL/S5/44664	F	19	*Mn.uniformis*	Flavi/Alphavirus	Negative
12/17/2023	Kwale	KWL/S5/44665	F	1	*Ae.circumluteolus*	Flavi/Alphavirus	Negative
12/17/2023	Kwale	KWL/S5/44666	F	1	*Ae.quasiunivittatus*	Flavi/Alphavirus	Negative
12/17/2023	Kwale	KWL/S5/44667	F	1	*Coq.metallicus*	Flavi/Alphavirus	Negative
12/17/2023	Kwale	KWL/S5/44668	F	5	*Ae.simpsoni*	Flavi/Alphavirus	Negative
12/17/2023	Kwale	KWL/S5/44669	F	9	*An.coustani*	Flavi/Alphavirus	Negative
12/17/2023	Kwale	KWL/S5/44670	F	5	*Ae.aegypti*	Flavi/Alphavirus	Negative
12/17/2023	Kwale	KWL/S5/44671	F	17	*An.funestus*	Flavi/Alphavirus	Negative
12/17/2023	Kwale	KWL/S5/44672	F	7	*An.squamosus*	Flavi/Alphavirus	Negative
12/17/2023	Kwale	KWL/S5/44673	F	1	*Cx.rubinotus*	Flavi/Alphavirus	Negative
12/17/2023	Kwale	KWL/S5/44674	F	10	*Cx.univittatus*	Flavi/Alphavirus	Negative
12/17/2023	Kwale	KWL/S5/44675	F	19	*Cx.annulioris*	Flavi/Alphavirus	Negative
12/17/2023	Kwale	KWL/S5/44676	F	25	*Ae.pembaensis*	Flavi/Alphavirus	Negative
12/17/2023	Kwale	KWL/S5/44677	F	5	*Ae.pembaensis*	Flavi/Alphavirus	Negative
12/17/2023	Kwale	KWL/S5/44678	F	6	*Mn.africana*	Flavi/Alphavirus	Negative
12/17/2023	Kwale	KWL/S5/44679	F	25	*Mn.uniformis*	Flavi/Alphavirus	Negative
12/17/2023	Kwale	KWL/S5/44680	F	25	*Mn.uniformis*	Flavi/Alphavirus	Negative
12/17/2023	Kwale	KWL/S5/44681	F	23	*Mn.uniformis*	Flavi/Alphavirus	Negative
12/17/2023	Kwale	KWL/S5/44682	F	4	*Ae.simpsoni*	Flavi/Alphavirus	Negative

12/17/2023	Kwale	KWL/S5/44683	F	3	*Ae.aegypti*	Flavi/Alphavirus	Negative
12/17/2023	Kwale	KWL/S5/44684	F	9	*Cx.univittatus*	Flavi/Alphavirus	Negative
12/17/2023	Kwale	KWL/S5/44685	F	6	*Ae.pembaensis*	Flavi/Alphavirus	Negative
12/17/2023	Kwale	KWL/S5/44686	F	13	*Cx.annulioris*	Flavi/Alphavirus	Negative
12/17/2023	Kwale	KWL/S5/44687	F	25	*Mn.uniformis*	Flavi/Alphavirus	Negative
12/17/2023	Kwale	KWL/S5/44688	F	8	*Mn.uniformis*	Flavi/Alphavirus	Negative
12/17/2023	Kwale	KWL/S5/44689	F	1	*Coq.aurites*	Flavi/Alphavirus	Negative
12/17/2023	Kwale	KWL/S5/44690	F	2	*Ae.quasiunivittatus*	Flavi/Alphavirus	Negative
12/17/2023	Kwale	KWL/S5/44691	F	3	*An.squamosus*	Flavi/Alphavirus	Negative
12/17/2023	Kwale	KWL/S5/44692	F	9	*Cx.annulioris*	Flavi/Alphavirus	Negative
12/17/2023	Kwale	KWL/S5/44693	F	14	*Cx.univittatus*	Flavi/Alphavirus	Negative
12/17/2023	Kwale	KWL/S5/44694	F	11	*Cx.pipiens*	Flavi/Alphavirus	Negative
12/17/2023	Kwale	KWL/S5/44695	F	24	*Ae.pembaensis*	Flavi/Alphavirus	Negative
12/17/2023	Kwale	KWL/S5/44696	F	25	*Mn.uniformis*	Flavi/Alphavirus	Negative
12/17/2023	Kwale	KWL/S5/44697	F	25	*Mn.uniformis*	Flavi/Alphavirus	Negative
12/17/2023	Kwale	KWL/S5/44698	F	25	*Mn.uniformis*	Flavi/Alphavirus	Negative
12/17/2023	Kwale	KWL/S5/44699	F	25	*Mn.uniformis*	Flavi/Alphavirus	Negative
12/17/2023	Kwale	KWL/S5/44700	F	5	*Mn.uniformis*	Flavi/Alphavirus	Negative
12/17/2023	Kwale	KWL/S5/44701	F	7	*Ae.simpsoni*	Flavi/Alphavirus	Negative
12/17/2023	Kwale	KWL/S5/44702	F	7	*Coq.aurites*	Flavi/Alphavirus	Negative
12/17/2023	Kwale	KWL/S5/44703	F	1	*An.gambiae*	Flavi/Alphavirus	Negative
12/17/2023	Kwale	KWL/S5/44704	F	13	*An.funestus*	Flavi/Alphavirus	Negative
12/17/2023	Kwale	KWL/S5/44705	F	2	*An.squamosus*	Flavi/Alphavirus	Negative
12/17/2023	Kwale	KWL/S5/44706	F	8	*An.coustani*	Flavi/Alphavirus	Negative
12/17/2023	Kwale	KWL/S6/44707	F	5	*Cx.pipiens*	Flavi/Alphavirus	Negative
12/17/2023	Kwale	KWL/S6/44708	M	1	*Ae.aegypti*	Flavi/Alphavirus	Negative
12/17/2023	Kwale	KWL/S6/44709	F	1	*Cx.annulioris*	Flavi/Alphavirus	Negative
12/17/2023	Kwale	KWL/S6/44710	F	1	*Cx.annulioris*	Flavi/Alphavirus	Negative
4/24/2024	Kwale	KWL/S1/44711	F	7	*Ae.aegypti*	Flavi/Alphavirus	Negative
4/24/2024	Kwale	KWL/S1/44712	M	5	*Ae.aegypti*	Flavi/Alphavirus	Negative
4/24/2024	Kwale	KWL/S1/44713	F	1	*Mn.africana*	Flavi/Alphavirus	Negative
4/24/2024	Kwale	KWL/S1/44714	F	3	*Mn.uniformis*	Flavi/Alphavirus	Negative
4/24/2024	Kwale	KWL/S1/44715	F	2	*Cx.annulioris*	Flavi/Alphavirus	Negative
4/24/2024	Kwale	KWL/S1/44716	M	1	*Ae.aegypti*	Flavi/Alphavirus	Negative
4/24/2024	Kwale	KWL/S1/44717	F	25	*Ae.aegypti*	Flavi/Alphavirus	Negative
4/24/2024	Kwale	KWL/S1/44718	F	9	*Ae.aegypti*	Flavi/Alphavirus	Negative
4/24/2024	Kwale	KWL/S1/44719	F	4	*Mn.uniformis*	Flavi/Alphavirus	Negative
4/24/2024	Kwale	KWL/S1/44720	F	4	*Cx.pipiens*	Flavi/Alphavirus	Negative
4/24/2024	Kwale	KWL/S1/44721	F	11	*Cx.annulioris*	Flavi/Alphavirus	Negative
4/24/2024	Kwale	KWL/S1/44722	F	1	*Cx.univittatus*	Flavi/Alphavirus	Negative
4/24/2024	Kwale	KWL/S1/44723	F	2	*Mn.uniformis*	Flavi/Alphavirus	Negative
4/24/2024	Kwale	KWL/S1/44724	F	4	*Cx.pipiens*	Flavi/Alphavirus	Negative
4/24/2024	Kwale	KWL/S1/44725	F	8	*Ae.aegypti*	Flavi/Alphavirus	Negative
4/24/2024	Kwale	KWL/S1/44726	F	1	*Cx.annulioris*	Flavi/Alphavirus	Negative
4/24/2024	Kwale	KWL/S1/44727	F	15	*Ae.aegypti*	Flavi/Alphavirus	Negative
4/24/2024	Kwale	KWL/S1/44728	F	3	*Cx.pipiens*	Flavi/Alphavirus	Negative
4/24/2024	Kwale	KWL/S1/44729	F	1	*Cx.pipiens*	Flavi/Alphavirus	Negative
4/24/2024	Kwale	KWL/S1/44730	F	5	*Cx.annulioris*	Flavi/Alphavirus	Negative
4/24/2024	Kwale	KWL/S1/44731	F	6	*Ae.aegypti*	Flavi/Alphavirus	Negative
4/24/2024	Kwale	KWL/S1/44732	F	2	*Mn.uniformis*	Flavi/Alphavirus	Negative

4/24/2024	Kwale	KWL/S1/44733	F	1	*Ae.aegypti*	Flavi/Alphavirus	Negative
4/24/2024	Kwale	KWL/S1/44734	F	5	*Ae.aegypti*	Flavi/Alphavirus	Negative
4/24/2024	Kwale	KWL/S1/44735	M	2	*Ae.aegypti*	Flavi/Alphavirus	Negative
4/24/2024	Kwale	KWL/S1/44736	F	2	*Mn.uniformis*	Flavi/Alphavirus	Negative
4/24/2024	Kwale	KWL/S1/44737	F	20	*Ae.aegypti*	Flavi/Alphavirus	Negative
4/24/2024	Kwale	KWL/S1/44738	F	10	*Cx.annulioris*	Flavi/Alphavirus	Negative
4/24/2024	Kwale	KWL/S1/44739	F	2	*Mn.uniformis*	Flavi/Alphavirus	Negative
4/24/2024	Kwale	KWL/S1/44740	F	1	*Cx.univittatus*	Flavi/Alphavirus	Negative
4/24/2024	Kwale	KWL/S1/44741	M	2	*Ae.aegypti*	Flavi/Alphavirus	Negative
4/25/2024	Kwale	KWL/S5/44742	F	1	*Mn.africana*	Flavi/Alphavirus	Negative
4/25/2024	Kwale	KWL/S5/44743	F	1	*Mn.uniformis*	Flavi/Alphavirus	Negative
4/25/2024	Kwale	KWL/S5/44744	F	4	*Ae.aegypti*	Flavi/Alphavirus	Negative
4/25/2024	Kwale	KWL/S5/44745	F	1	*An.gambiae*	Flavi/Alphavirus	Negative
4/25/2024	Kwale	KWL/S5/44746	F	1	*Ae.aegypti*	Flavi/Alphavirus	Negative
4/25/2024	Kwale	KWL/S5/44747	F	3	*Mn.africana*	Flavi/Alphavirus	Negative
4/25/2024	Kwale	KWL/S5/44748	F	25	*Mn.uniformis*	Flavi/Alphavirus	Negative
4/25/2024	Kwale	KWL/S5/44749	F	7	*Mn.uniformis*	Flavi/Alphavirus	Negative
4/25/2024	Kwale	KWL/S5/44750	F	3	*Cx.annulioris*	Flavi/Alphavirus	Negative
4/25/2024	Kwale	KWL/S5/44751	F	3	*Ae.dentatus*	Flavi/Alphavirus	Negative
4/25/2024	Kwale	KWL/S5/44752	F	4	*Mn.uniformis*	Flavi/Alphavirus	Negative
4/25/2024	Kwale	KWL/S5/44753	F	3	*Cx.annulioris*	Flavi/Alphavirus	Negative
4/25/2024	Kwale	KWL/S5/44754	F	1	*Cx.univittatus*	Flavi/Alphavirus	Negative
4/25/2024	Kwale	KWL/S5/44755	F	6	*Cx.annulioris*	Flavi/Alphavirus	Negative
4/25/2024	Kwale	KWL/S5/44756	F	25	*Mn.uniformis*	Flavi/Alphavirus	Negative
4/25/2024	Kwale	KWL/S5/44757	F	13	*Mn.uniformis*	Flavi/Alphavirus	Negative
4/25/2024	Kwale	KWL/S5/44758	F	1	*Cx.pipiens*	Flavi/Alphavirus	Negative
4/25/2024	Kwale	KWL/S5/44759	F	2	*Coq.metallicus*	Flavi/Alphavirus	Negative
4/25/2024	Kwale	KWL/S5/44760	F	1	*Coq.metallicus*	Flavi/Alphavirus	Negative
4/25/2024	Kwale	KWL/S5/44761	F	2	*Ae.dentatus*	Flavi/Alphavirus	Negative
4/25/2024	Kwale	KWL/S5/44762	F	1	*Ae.circumluteolus*	Flavi/Alphavirus	Negative
4/25/2024	Kwale	KWL/S5/44763	F	2	*Mn.africana*	Flavi/Alphavirus	Negative
4/25/2024	Kwale	KWL/S5/44764	F	11	*Cx.annulioris*	Flavi/Alphavirus	Negative
4/25/2024	Kwale	KWL/S5/44765	F	5	*Ae.aegypti*	Flavi/Alphavirus	Negative
4/25/2024	Kwale	KWL/S5/44766	F	17	*Cx.pipiens*	Flavi/Alphavirus	Negative
4/25/2024	Kwale	KWL/S5/44767	F	25	*Mn.uniformis*	Flavi/Alphavirus	Negative
4/25/2024	Kwale	KWL/S5/44768	F	25	*Mn.uniformis*	Flavi/Alphavirus	Negative
4/25/2024	Kwale	KWL/S5/44769	F	25	*Mn.uniformis*	Flavi/Alphavirus	Negative
4/25/2024	Kwale	KWL/S5/44770	F	23	*Mn.uniformis*	Flavi/Alphavirus	Negative
4/25/2024	Kwale	KWL/S5/44771	F	25	*Mn.uniformis*	Flavi/Alphavirus	Negative
4/25/2024	Kwale	KWL/S5/44772	F	25	*Mn.uniformis*	Flavi/Alphavirus	Negative
4/25/2024	Kwale	KWL/S5/44773	F	25	*Mn.uniformis*	Flavi/Alphavirus	Negative
4/25/2024	Kwale	KWL/S5/44774	F	1	*An.funestus*	Flavi/Alphavirus	Negative
4/25/2024	Kwale	KWL/S5/44775	F	21	*Ae.dentatus*	Flavi/Alphavirus	Negative
4/25/2024	Kwale	KWL/S5/44776	F	1	*Ae.circumluteolus*	Flavi/Alphavirus	Negative
4/25/2024	Kwale	KWL/S5/44777	M	1	*Ae.aegypti*	Flavi/Alphavirus	Negative
4/25/2024	Kwale	KWL/S5/44778	F	6	*Ae.aegypti*	Flavi/Alphavirus	Negative
4/25/2024	Kwale	KWL/S5/44779	F	19	*Cx.annulioris*	Flavi/Alphavirus	Negative
4/25/2024	Kwale	KWL/S5/44780	F	10	*Cx.pipiens*	Flavi/Alphavirus	Negative
4/25/2024	Kwale	KWL/S5/44781	F	3	*Cx.univittatus*	Flavi/Alphavirus	Negative
4/25/2024	Kwale	KWL/S5/44782	F	1	*Cx.bitaeniorhynchus*	Flavi/Alphavirus	Negative

4/25/2024	Kwale	KWL/S5/44783	F	25	*Mn.uniformis*	Flavi/Alphavirus	Negative
4/25/2024	Kwale	KWL/S5/44784	F	25	*Mn.uniformis*	Flavi/Alphavirus	Negative
4/25/2024	Kwale	KWL/S5/44785	F	25	*Mn.uniformis*	Flavi/Alphavirus	Negative
4/25/2024	Kwale	KWL/S5/44786	F	25	*Mn.uniformis*	Flavi/Alphavirus	Negative
4/25/2024	Kwale	KWL/S5/44787	F	6	*Mn.uniformis*	Flavi/Alphavirus	Negative
4/25/2024	Kwale	KWL/S5/44788	F	1	*Ae.aegypti*	Flavi/Alphavirus	Negative
4/25/2024	Kwale	KWL/S5/44789	F	1	*Mn.africana*	Flavi/Alphavirus	Negative
4/25/2024	Kwale	KWL/S5/44790	F	1	*An.funestus*	Flavi/Alphavirus	Negative
4/25/2024	Kwale	KWL/S5/44791	F	3	*Cx.univittatus*	Flavi/Alphavirus	Negative
4/25/2024	Kwale	KWL/S5/44792	F	10	*Cx.pipiens*	Flavi/Alphavirus	Negative
4/25/2024	Kwale	KWL/S5/44793	F	9	*Cx.annulioris*	Flavi/Alphavirus	Negative
4/25/2024	Kwale	KWL/S5/44794	F	7	*Coq.metallicus*	Flavi/Alphavirus	Negative
4/25/2024	Kwale	KWL/S5/44795	F	12	*Ae.dentatus*	Flavi/Alphavirus	Negative
4/25/2024	Kwale	KWL/S5/44796	F	7	*Ae.circumluteolus*	Flavi/Alphavirus	Negative
4/25/2024	Kwale	KWL/S5/44797	F	2	*Coq.metallicus*	Flavi/Alphavirus	Negative
4/25/2024	Kwale	KWL/S5/44798	F	8	*Ae.circumluteolus*	Flavi/Alphavirus	Negative
4/25/2024	Kwale	KWL/S5/44799	F	4	*Mn.africana*	Flavi/Alphavirus	Negative
4/25/2024	Kwale	KWL/S5/44800	F	10	*Cx.pipiens*	Flavi/Alphavirus	Negative
4/25/2024	Kwale	KWL/S5/44801	F	8	*Ae.aegypti*	Flavi/Alphavirus	Negative
4/25/2024	Kwale	KWL/S5/44802	M	2	*Ae.aegypti*	Flavi/Alphavirus	Negative
4/25/2024	Kwale	KWL/S5/44803	F	10	*Cx.univittatus*	Flavi/Alphavirus	Negative
4/25/2024	Kwale	KWL/S5/44804	F	9	*Cx.annulioris*	Flavi/Alphavirus	Negative
4/25/2024	Kwale	KWL/S5/44805	F	9	*Ae.dentatus*	Flavi/Alphavirus	Negative
4/25/2024	Kwale	KWL/S5/44806	F	25	*Mn.uniformis*	Flavi/Alphavirus	Negative
4/25/2024	Kwale	KWL/S5/44807	F	25	*Mn.uniformis*	Flavi/Alphavirus	Negative
4/25/2024	Kwale	KWL/S5/44808	F	25	*Mn.uniformis*	Flavi/Alphavirus	Negative
4/25/2024	Kwale	KWL/S5/44809	F	25	*Mn.uniformis*	Flavi/Alphavirus	Negative
4/25/2024	Kwale	KWL/S5/44810	F	25	*Mn.uniformis*	Flavi/Alphavirus	Negative
4/25/2024	Kwale	KWL/S5/44811	F	16	*Mn.uniformis*	Flavi/Alphavirus	Negative
4/26/2024	Kwale	KWL/S4/44812	F	2	*Cx.univittatus*	Flavi/Alphavirus	Negative
4/26/2024	Kwale	KWL/S4/44813	F	1	*Coq.metallicus*	Flavi/Alphavirus	Negative
4/26/2024	Kwale	KWL/S4/44814	F	4	*Mn.uniformis*	Flavi/Alphavirus	Negative
4/26/2024	Kwale	KWL/S4/44815	F	14	*Cx.pipiens*	Flavi/Alphavirus	Negative
4/26/2024	Kwale	KWL/S4/44816	F	10	*Cx.annulioris*	Flavi/Alphavirus	Negative
4/26/2024	Kwale	KWL/S4/44817	F	7	*Ae.aegypti*	Flavi/Alphavirus	Negative
4/26/2024	Kwale	KWL/S4/44818	M	3	*Ae.aegypti*	Flavi/Alphavirus	Negative
4/26/2024	Kwale	KWL/S4/44819	F	2	*Ae.dentatus*	Flavi/Alphavirus	Negative
4/26/2024	Kwale	KWL/S4/44820	F	11	*Cx.pipiens*	Flavi/Alphavirus	Negative
4/26/2024	Kwale	KWL/S4/44821	F	3	*Cx.univittatus*	Flavi/Alphavirus	Negative
4/26/2024	Kwale	KWL/S4/44822	F	3	*Mn.africana*	Flavi/Alphavirus	Negative
4/26/2024	Kwale	KWL/S4/44823	F	25	*Mn.uniformis*	Flavi/Alphavirus	Negative
4/26/2024	Kwale	KWL/S4/44824	F	19	*Cx.annulioris*	Flavi/Alphavirus	Negative
4/26/2024	Kwale	KWL/S4/44825	F	4	*Ae.aegypti*	Flavi/Alphavirus	Negative
4/26/2024	Kwale	KWL/S4/44826	F	1	*An.gambiae*	Flavi/Alphavirus	Negative
4/26/2024	Kwale	KWL/S4/44827	F	1	*Mn.africana*	Flavi/Alphavirus	Negative
4/26/2024	Kwale	KWL/S4/44828	F	5	*Cx.annulioris*	Flavi/Alphavirus	Negative
4/26/2024	Kwale	KWL/S4/44829	F	3	*Cx.univittatus*	Flavi/Alphavirus	Negative
4/26/2024	Kwale	KWL/S4/44830	M	2	*Ae.aegypti*	Flavi/Alphavirus	Negative
4/26/2024	Kwale	KWL/S4/44831	F	7	*Ae.aegypti*	Flavi/Alphavirus	Negative
4/26/2024	Kwale	KWL/S4/44832	F	12	*Mn.uniformis*	Flavi/Alphavirus	Negative

4/26/2024	Kwale	KWL/S4/44833	F	11	*Cx.pipiens*	Flavi/Alphavirus	Negative
4/26/2024	Kwale	KWL/S4/44834	F	1	*Coq.metallicus*	Flavi/Alphavirus	Negative
4/26/2024	Kwale	KWL/S4/44835	F	25	*Mn.uniformis*	Flavi/Alphavirus	Negative
4/26/2024	Kwale	KWL/S4/44836	F	2	*Mn.africana*	Flavi/Alphavirus	Negative
4/26/2024	Kwale	KWL/S4/44837	F	1	*Cx.univittatus*	Flavi/Alphavirus	Negative
4/26/2024	Kwale	KWL/S4/44838	F	3	*Ae.dentatus*	Flavi/Alphavirus	Negative
4/26/2024	Kwale	KWL/S4/44839	M	2	*Ae.aegypti*	Flavi/Alphavirus	Negative
4/26/2024	Kwale	KWL/S4/44840	F	2	*Ae.aegypti*	Flavi/Alphavirus	Negative
4/26/2024	Kwale	KWL/S4/44841	F	3	*Cx.pipiens*	Flavi/Alphavirus	Negative
4/26/2024	Kwale	KWL/S4/44842	F	12	*Cx.annulioris*	Flavi/Alphavirus	Negative
4/26/2024	Kwale	KWL/S4/44843	F	2	*An.funestus*	Flavi/Alphavirus	Negative
4/26/2024	Kwale	KWL/S4/44844	F	6	*Mn.uniformis*	Flavi/Alphavirus	Negative
4/26/2024	Kwale	KWL/S4/44845	F	4	*Ae.dentatus*	Flavi/Alphavirus	Negative
4/26/2024	Kwale	KWL/S4/44846	F	1	*Coq.metallicus*	Flavi/Alphavirus	Negative
4/26/2024	Kwale	KWL/S4/44847	F	1	*Mn.africana*	Flavi/Alphavirus	Negative
4/26/2024	Kwale	KWL/S4/44848	F	13	*Cx.pipiens*	Flavi/Alphavirus	Negative
4/26/2024	Kwale	KWL/S4/44849	F	12	*Cx.annulioris*	Flavi/Alphavirus	Negative
4/26/2024	Kwale	KWL/S4/44850	F	3	*Ae.aegypti*	Flavi/Alphavirus	Negative
4/26/2024	Kwale	KWL/S4/44851	F	2	*Aedeomyia furfurea*	Flavi/Alphavirus	Negative
4/26/2024	Kwale	KWL/S4/44852	F	1	*Mn.africana*	Flavi/Alphavirus	Negative
4/26/2024	Kwale	KWL/S4/44853	F	2	*Cx.annulioris*	Flavi/Alphavirus	Negative
4/26/2024	Kwale	KWL/S4/44854	F	2	*Cx.univittatus*	Flavi/Alphavirus	Negative
4/26/2024	Kwale	KWL/S4/44855	F	3	*Cx.pipiens*	Flavi/Alphavirus	Negative
4/26/2024	Kwale	KWL/S4/44856	F	7	*Mn.uniformis*	Flavi/Alphavirus	Negative
4/26/2024	Kwale	KWL/S4/44857	F	10	*Cx.pipiens*	Flavi/Alphavirus	Negative
4/26/2024	Kwale	KWL/S4/44858	F	6	*Mn.uniformis*	Flavi/Alphavirus	Negative
4/26/2024	Kwale	KWL/S4/44859	F	1	*Cx.univittatus*	Flavi/Alphavirus	Negative
4/26/2024	Kwale	KWL/S4/44860	F	1	*Coq.metallicus*	Flavi/Alphavirus	Negative
4/26/2024	Kwale	KWL/S4/44861	F	5	*Ae.dentatus*	Flavi/Alphavirus	Negative
4/26/2024	Kwale	KWL/S4/44862	F	14	*Cx.annulioris*	Flavi/Alphavirus	Negative
4/26/2024	Kwale	KWL/S4/44863	F	1	*Cx.bitaeniorhynchus*	Flavi/Alphavirus	Negative
4/26/2024	Kwale	KWL/S4/44864	M	3	*Ae.aegypti*	Flavi/Alphavirus	Negative
4/26/2024	Kwale	KWL/S4/44865	F	4	*Ae.aegypti*	Flavi/Alphavirus	Negative
4/26/2024	Kwale	KWL/S4/44866	F	1	*Cx.bitaeniorhynchus*	Flavi/Alphavirus	Negative
4/26/2024	Kwale	KWL/S4/44867	F	11	*Mn.uniformis*	Flavi/Alphavirus	Negative
4/26/2024	Kwale	KWL/S4/44868	F	1	*Cx.univittatus*	Flavi/Alphavirus	Negative
4/26/2024	Kwale	KWL/S4/44869	F	2	*Coq.metallicus*	Flavi/Alphavirus	Negative
4/26/2024	Kwale	KWL/S4/44870	F	11	*Ae.dentatus*	Flavi/Alphavirus	Negative
4/26/2024	Kwale	KWL/S4/44871	F	14	*Cx.annulioris*	Flavi/Alphavirus	Negative
4/26/2024	Kwale	KWL/S4/44872	F	18	*Cx.pipiens*	Flavi/Alphavirus	Negative
4/26/2024	Kwale	KWL/S4/44873	F	8	*Ae.aegypti*	Flavi/Alphavirus	Negative
4/26/2024	Kwale	KWL/S4/44874	M	3	*Ae.aegypti*	Flavi/Alphavirus	Negative
4/27/2024	Kwale	KWL/S1/44875	F	2	*Aedeomyia furfurea*	Flavi/Alphavirus	Negative
4/27/2024	Kwale	KWL/S1/44876	F	3	*Ae.pembaensis*	Flavi/Alphavirus	Negative
4/27/2024	Kwale	KWL/S1/44877	F	1	*Mn.uniformis*	Flavi/Alphavirus	Negative
4/27/2024	Kwale	KWL/S1/44878	F	4	*Cx.annulioris*	Flavi/Alphavirus	Negative
4/27/2024	Kwale	KWL/S1/44879	F	4	*Cx.univittatus*	Flavi/Alphavirus	Negative
4/27/2024	Kwale	KWL/S1/44880	F	5	*Cx.pipiens*	Flavi/Alphavirus	Negative
4/27/2024	Kwale	KWL/S1/44881	F	2	*Coq.metallicus*	Flavi/Alphavirus	Negative
4/27/2024	Kwale	KWL/S1/44882	F	8	*Ae.aegypti*	Flavi/Alphavirus	Negative

4/27/2024	Kwale	KWL/S1/44883	M	3	*Ae.aegypti*	Flavi/Alphavirus	Negative
4/27/2024	Kwale	KWL/S1/44884	F	1	*Eret.chryogaster*	Flavi/Alphavirus	Negative
4/27/2024	Kwale	KWL/S1/44885	F	2	*Ae.pembaensis*	Flavi/Alphavirus	Negative
4/27/2024	Kwale	KWL/S1/44886	F	4	*Cx.pipiens*	Flavi/Alphavirus	Negative
4/27/2024	Kwale	KWL/S1/44887	F	5	*Cx.annulioris*	Flavi/Alphavirus	Negative
4/27/2024	Kwale	KWL/S1/44888	F	2	*Coq.metallicus*	Flavi/Alphavirus	Negative
4/27/2024	Kwale	KWL/S1/44889	F	2	*Cx.univittatus*	Flavi/Alphavirus	Negative
4/27/2024	Kwale	KWL/S1/44890	F	12	*Mn.uniformis*	Flavi/Alphavirus	Negative
4/27/2024	Kwale	KWL/S1/44891	F	18	*Ae.aegypti*	Flavi/Alphavirus	Negative
4/27/2024	Kwale	KWL/S1/44892	M	6	*Ae.aegypti*	Flavi/Alphavirus	Negative
4/27/2024	Kwale	KWL/S1/44893	F	3	*Coq.metallicus*	Flavi/Alphavirus	Negative
4/27/2024	Kwale	KWL/S1/44894	F	1	*Eret.chryogaster*	Flavi/Alphavirus	Negative
4/27/2024	Kwale	KWL/S1/44895	F	3	*Ae.pembaensis*	Flavi/Alphavirus	Negative
4/27/2024	Kwale	KWL/S1/44896	F	6	*Cx.annulioris*	Flavi/Alphavirus	Negative
4/27/2024	Kwale	KWL/S1/44897	F	14	*Cx.pipiens*	Flavi/Alphavirus	Negative
4/27/2024	Kwale	KWL/S1/44898	F	1	*Cx.univittatus*	Flavi/Alphavirus	Negative
4/27/2024	Kwale	KWL/S1/44899	F	16	*Mn.uniformis*	Flavi/Alphavirus	Negative
4/27/2024	Kwale	KWL/S1/44900	F	13	*Ae.aegypti*	Flavi/Alphavirus	Negative
4/27/2024	Kwale	KWL/S1/44901	F	3	*Ae.aegypti*	Flavi/Alphavirus	Positive - WNV
4/27/2024	Kwale	KWL/S1/44902	F	1	*Coq.metallicus*	Flavi/Alphavirus	Negative
4/27/2024	Kwale	KWL/S1/44903	F	2	*Cx.annulioris*	Flavi/Alphavirus	Negative
4/27/2024	Kwale	KWL/S1/44904	F	1	*Ae.aegypti*	Flavi/Alphavirus	Positive - WNV
4/27/2024	Kwale	KWL/S1/44905	F	8	*Cx.pipiens*	Flavi/Alphavirus	Negative
4/27/2024	Kwale	KWL/S1/44906	F	9	*Ae.aegypti*	Flavi/Alphavirus	Negative
4/27/2024	Kwale	KWL/S1/44907	F	1	*Cx.annulioris*	Flavi/Alphavirus	Negative
4/27/2024	Kwale	KWL/S1/44908	F	3	*Cx.pipiens*	Flavi/Alphavirus	Negative
4/27/2024	Kwale	KWL/S1/44909	F	25	*Ae.aegypti*	Flavi/Alphavirus	Negative
4/27/2024	Kwale	KWL/S1/44910	M	3	*Ae.aegypti*	Flavi/Alphavirus	Negative
4/27/2024	Kwale	KWL/S1/44911	F	5	*Mn.uniformis*	Flavi/Alphavirus	Negative
4/27/2024	Kwale	KWL/S1/44912	F	1	*Coq.metallicus*	Flavi/Alphavirus	Negative
4/27/2024	Kwale	KWL/S1/44913	F	5	*Ae.pembaensis*	Flavi/Alphavirus	Negative
4/27/2024	Kwale	KWL/S1/44914	F	8	*Cx.annulioris*	Flavi/Alphavirus	Negative
4/27/2024	Kwale	KWL/S1/44915	F	7	*Cx.pipiens*	Flavi/Alphavirus	Negative
4/27/2024	Kwale	KWL/S1/44916	F	3	*Cx.annulioris*	Flavi/Alphavirus	Negative
4/27/2024	Kwale	KWL/S1/44917	F	2	*Cx.pipiens*	Flavi/Alphavirus	Negative
4/27/2024	Kwale	KWL/S1/44918	F	1	*Coq.metallicus*	Flavi/Alphavirus	Negative
4/27/2024	Kwale	KWL/S1/44919	F	1	*Mn.uniformis*	Flavi/Alphavirus	Negative
4/27/2024	Kwale	KWL/S1/44920	F	1	*Ae.pembaensis*	Flavi/Alphavirus	Negative
4/27/2024	Kwale	KWL/S1/44921	F	8	*Ae.aegypti*	Flavi/Alphavirus	Negative
4/27/2024	Kwale	KWL/S1/44922	F	2	*Cx.univittatus*	Flavi/Alphavirus	Negative
4/27/2024	Kwale	KWL/S1/44923	F	6	*Mn.uniformis*	Flavi/Alphavirus	Negative
4/27/2024	Kwale	KWL/S1/44924	F	2	*Cx.annulioris*	Flavi/Alphavirus	Negative
4/27/2024	Kwale	KWL/S1/44925	F	8	*Cx.pipiens*	Flavi/Alphavirus	Negative
4/27/2024	Kwale	KWL/S1/44926	F	6	*Ae.aegypti*	Flavi/Alphavirus	Negative
4/27/2024	Kwale	KWL/S1/44927	M	1	*Ae.aegypti*	Flavi/Alphavirus	Negative
4/28/2024	Kwale	KWL/S5/44928	F	3	*Cx.pipiens*	Flavi/Alphavirus	Negative
4/28/2024	Kwale	KWL/S5/44929	F	1	*Ae.aegypti*	Flavi/Alphavirus	Negative
4/28/2024	Kwale	KWL/S5/44930	F	2	*Mn.africana*	Flavi/Alphavirus	Negative
4/28/2024	Kwale	KWL/S5/44931	F	6	*Cx.univittatus*	Flavi/Alphavirus	Negative
4/28/2024	Kwale	KWL/S5/44932	F	18	*Mn.uniformis*	Flavi/Alphavirus	Negative

4/28/2024	Kwale	KWL/S5/44933	F	5	*Cx.univittatus*	Flavi/Alphavirus	Negative
4/28/2024	Kwale	KWL/S5/44934	F	12	*Mn.uniformis*	Flavi/Alphavirus	Negative
4/28/2024	Kwale	KWL/S5/44935	F	5	*Ae.aegypti*	Flavi/Alphavirus	Negative
4/28/2024	Kwale	KWL/S5/44936	F	1	*Cx.annulioris*	Flavi/Alphavirus	Negative
4/28/2024	Kwale	KWL/S5/44937	F	1	*Cx.pipiens*	Flavi/Alphavirus	Negative
4/28/2024	Kwale	KWL/S5/44938	F	1	*Coq.metallicus*	Flavi/Alphavirus	Negative
4/28/2024	Kwale	KWL/S5/44939	F	1	*Ae.pembaensis*	Flavi/Alphavirus	Negative
4/28/2024	Kwale	KWL/S5/44940	F	3	*Cx.pipiens*	Flavi/Alphavirus	Negative
4/28/2024	Kwale	KWL/S5/44941	F	5	*Coq.metallicus*	Flavi/Alphavirus	Negative
4/28/2024	Kwale	KWL/S5/44942	F	5	*Cx.annulioris*	Flavi/Alphavirus	Negative
4/28/2024	Kwale	KWL/S5/44943	F	6	*Cx.univittatus*	Flavi/Alphavirus	Negative
4/28/2024	Kwale	KWL/S5/44944	F	5	*Ae.aegypti*	Flavi/Alphavirus	Negative
4/28/2024	Kwale	KWL/S5/44945	F	9	*Mn.africana*	Flavi/Alphavirus	Negative
4/28/2024	Kwale	KWL/S5/44946	F	25	*Mn.uniformis*	Flavi/Alphavirus	Negative
4/28/2024	Kwale	KWL/S5/44947	F	25	*Mn.uniformis*	Flavi/Alphavirus	Negative
4/28/2024	Kwale	KWL/S5/44948	F	19	*Mn.uniformis*	Flavi/Alphavirus	Negative
4/28/2024	Kwale	KWL/S5/44949	F	1	*Ae.aegypti*	Flavi/Alphavirus	Negative
4/28/2024	Kwale	KWL/S5/44950	F	1	*An.coustani*	Flavi/Alphavirus	Negative
4/28/2024	Kwale	KWL/S5/44951	F	2	*Ae.pembaensis*	Flavi/Alphavirus	Negative
4/28/2024	Kwale	KWL/S5/44952	F	5	*Cx.annulioris*	Flavi/Alphavirus	Negative
4/28/2024	Kwale	KWL/S5/44953	F	1	*Cx.univittatus*	Flavi/Alphavirus	Negative
4/28/2024	Kwale	KWL/S5/44954	F	5	*Mn.africana*	Flavi/Alphavirus	Negative
4/28/2024	Kwale	KWL/S5/44955	F	25	*Mn.uniformis*	Flavi/Alphavirus	Negative
4/28/2024	Kwale	KWL/S5/44956	F	9	*Mn.uniformis*	Flavi/Alphavirus	Negative
4/28/2024	Kwale	KWL/S5/44957	F	1	*Cx.pipiens*	Flavi/Alphavirus	Negative
4/28/2024	Kwale	KWL/S5/44958	F	1	*Ae.pembaensis*	Flavi/Alphavirus	Negative
4/28/2024	Kwale	KWL/S5/44959	F	2	*Ae.aegypti*	Flavi/Alphavirus	Negative
4/28/2024	Kwale	KWL/S5/44960	F	1	*Ae.circumluteolus*	Flavi/Alphavirus	Negative
4/28/2024	Kwale	KWL/S5/44961	F	3	*Cx.pipiens*	Flavi/Alphavirus	Negative
4/28/2024	Kwale	KWL/S5/44962	F	5	*Cx.univittatus*	Flavi/Alphavirus	Negative
4/28/2024	Kwale	KWL/S5/44963	F	1	*Coq.metallicus*	Flavi/Alphavirus	Negative
4/28/2024	Kwale	KWL/S5/44964	F	4	*Cx.annulioris*	Flavi/Alphavirus	Negative
4/28/2024	Kwale	KWL/S5/44965	F	5	*Mn.africana*	Flavi/Alphavirus	Negative
4/28/2024	Kwale	KWL/S5/44966	F	25	*Mn.uniformis*	Flavi/Alphavirus	Negative
4/28/2024	Kwale	KWL/S5/44967	F	1	*Cx.pipiens*	Flavi/Alphavirus	Negative
4/28/2024	Kwale	KWL/S5/44968	F	1	*Ae.aegypti*	Flavi/Alphavirus	Negative
4/28/2024	Kwale	KWL/S5/44969	F	5	*Mn.uniformis*	Flavi/Alphavirus	Negative
4/28/2024	Kwale	KWL/S5/44970	F	5	*Ae.aegypti*	Flavi/Alphavirus	Negative
4/28/2024	Kwale	KWL/S5/44971	F	6	*Cx.pipiens*	Flavi/Alphavirus	Negative
4/28/2024	Kwale	KWL/S5/44972	F	4	*Ae.pembaensis*	Flavi/Alphavirus	Negative
4/28/2024	Kwale	KWL/S5/44973	F	8	*Cx.annulioris*	Flavi/Alphavirus	Negative
4/28/2024	Kwale	KWL/S5/44974	F	11	*Cx.univittatus*	Flavi/Alphavirus	Negative
4/28/2024	Kwale	KWL/S5/44975	F	7	*Coq.metallicus*	Flavi/Alphavirus	Negative
4/28/2024	Kwale	KWL/S5/44976	F	7	*Mn.africana*	Flavi/Alphavirus	Negative
4/28/2024	Kwale	KWL/S5/44977	F	25	*Mn.uniformis*	Flavi/Alphavirus	Negative
4/28/2024	Kwale	KWL/S5/44978	F	25	*Mn.uniformis*	Flavi/Alphavirus	Negative
4/28/2024	Kwale	KWL/S5/44979	F	25	*Mn.uniformis*	Flavi/Alphavirus	Negative
4/28/2024	Kwale	KWL/S5/44980	F	8	*Mn.uniformis*	Flavi/Alphavirus	Negative
4/28/2024	Kwale	KWL/S5/44981	F	9	*Mn.africana*	Flavi/Alphavirus	Negative
4/28/2024	Kwale	KWL/S5/44982	F	8	*Cx.pipiens*	Flavi/Alphavirus	Negative

4/28/2024	Kwale	KWL/S5/44983	F	2	*Ae.aegypti*	Flavi/Alphavirus	Negative
4/28/2024	Kwale	KWL/S5/44984	F	3	*Coq.metallicus*	Flavi/Alphavirus	Negative
4/28/2024	Kwale	KWL/S5/44985	F	2	*Ae.pembaensis*	Flavi/Alphavirus	Negative
4/28/2024	Kwale	KWL/S5/44986	F	25	*Mn.uniformis*	Flavi/Alphavirus	Negative
4/28/2024	Kwale	KWL/S5/44987	F	20	*Mn.uniformis*	Flavi/Alphavirus	Negative
5/30/2024	Mombasa	MBSA/S7/44888	F	4	*Ae.aegypti*	Flavi/Alphavirus	Negative
5/30/2024	Mombasa	MBSA/S7/44889	F	9	*Cx.pipiens*	Flavi/Alphavirus	Negative
5/30/2024	Mombasa	MBSA/S7/44890	M	1	*Ae.aegypti*	Flavi/Alphavirus	Negative
5/30/2024	Mombasa	MBSA/S7/44891	F	9	*Ae.aegypti*	Flavi/Alphavirus	Negative
5/30/2024	Mombasa	MBSA/S7/44892	F	1	*Ae.vittatus*	Flavi/Alphavirus	Negative
5/30/2024	Mombasa	MBSA/S7/44893	F	25	*Cx.pipiens*	Flavi/Alphavirus	Negative
5/30/2024	Mombasa	MBSA/S7/44894	F	2	*Cx.pipiens*	Flavi/Alphavirus	Negative
5/30/2024	Mombasa	MBSA/S7/44895	F	1	*Mn.uniformis*	Flavi/Alphavirus	Negative
5/30/2024	Mombasa	MBSA/S7/44896	F	3	*Ae.aegypti*	Flavi/Alphavirus	Negative
5/30/2024	Mombasa	MBSA/S7/44897	F	25	*Cx.pipiens*	Flavi/Alphavirus	Negative
5/30/2024	Mombasa	MBSA/S7/44898	F	25	*Cx.pipiens*	Flavi/Alphavirus	Negative
5/30/2024	Mombasa	MBSA/S7/44899	F	18	*Cx.pipiens*	Flavi/Alphavirus	Negative
5/30/2024	Mombasa	MBSA/S7/44900	M	16	*Ae.aegypti*	Flavi/Alphavirus	Negative
5/30/2024	Mombasa	MBSA/S7/44901	F	13	*Ae.aegypti*	Flavi/Alphavirus	Negative
5/30/2024	Mombasa	MBSA/S7/44902	F	3	*Cx.pipiens*	Flavi/Alphavirus	Negative
5/30/2024	Mombasa	MBSA/S7/44903	M	4	*Ae.aegypti*	Flavi/Alphavirus	Negative
5/30/2024	Mombasa	MBSA/S7/44904	F	14	*Ae.aegypti*	Flavi/Alphavirus	Negative
5/31/2024	Mombasa	MBSA/S9/44905	F	25	*Mn.africana*	Flavi/Alphavirus	Negative
5/31/2024	Mombasa	MBSA/S9/44906	F	25	*Mn.africana*	Flavi/Alphavirus	Negative
5/31/2024	Mombasa	MBSA/S9/44907	F	5	*Mn.africana*	Flavi/Alphavirus	Negative
5/31/2024	Mombasa	MBSA/S9/44908	F	1	*Ae.vittatus*	Flavi/Alphavirus	Negative
5/31/2024	Mombasa	MBSA/S9/44909	F	1	*Ae.aegypti*	Flavi/Alphavirus	Negative
5/31/2024	Mombasa	MBSA/S9/44910	F	25	*Mn.africana*	Flavi/Alphavirus	Negative
5/31/2024	Mombasa	MBSA/S9/44911	F	25	*Mn.africana*	Flavi/Alphavirus	Negative
5/31/2024	Mombasa	MBSA/S9/44912	F	25	*Mn.africana*	Flavi/Alphavirus	Negative
5/31/2024	Mombasa	MBSA/S9/44913	F	25	*Mn.africana*	Flavi/Alphavirus	Negative
5/31/2024	Mombasa	MBSA/S9/44914	F	25	*Mn.africana*	Flavi/Alphavirus	Negative
5/31/2024	Mombasa	MBSA/S9/44915	F	9	*Cx.univittatus*	Flavi/Alphavirus	Negative
5/31/2024	Mombasa	MBSA/S9/44916	F	2	*Cx.annulioris*	Flavi/Alphavirus	Negative
5/31/2024	Mombasa	MBSA/S9/44917	F	5	*An.squamosus*	Flavi/Alphavirus	Negative
5/31/2024	Mombasa	MBSA/S9/44918	F	1	*An.funestus*	Flavi/Alphavirus	Negative
5/31/2024	Mombasa	MBSA/S9/44919	F	25	*Mn.africana*	Flavi/Alphavirus	Negative
5/31/2024	Mombasa	MBSA/S9/44920	F	25	*Mn.africana*	Flavi/Alphavirus	Negative
5/31/2024	Mombasa	MBSA/S9/44921	F	25	*Mn.africana*	Flavi/Alphavirus	Negative
5/31/2024	Mombasa	MBSA/S9/44922	F	24	*Mn.africana*	Flavi/Alphavirus	Negative
5/31/2024	Mombasa	MBSA/S9/44923	F	16	*Cx.univittatus*	Flavi/Alphavirus	Negative
5/31/2024	Mombasa	MBSA/S9/44924	F	5	*An.squamosus*	Flavi/Alphavirus	Negative
5/31/2024	Mombasa	MBSA/S9/44925	F	1	*Cx.rubinotus*	Flavi/Alphavirus	Negative
5/31/2024	Mombasa	MBSA/S9/44926	F	1	*Ae.circumluteolus*	Flavi/Alphavirus	Negative
5/31/2024	Mombasa	MBSA/S9/44927	F	25	*Mn.africana*	Flavi/Alphavirus	Negative
5/31/2024	Mombasa	MBSA/S9/44928	F	25	*Mn.africana*	Flavi/Alphavirus	Negative
5/31/2024	Mombasa	MBSA/S9/44929	F	25	*Mn.africana*	Flavi/Alphavirus	Negative
5/31/2024	Mombasa	MBSA/S9/44930	F	16	*Mn.africana*	Flavi/Alphavirus	Negative
5/31/2024	Mombasa	MBSA/S9/44931	F	1	*Cx.poicilipes*	Flavi/Alphavirus	Negative
5/31/2024	Mombasa	MBSA/S9/44932	F	1	*An.squamosus*	Flavi/Alphavirus	Negative

5/31/2024	Mombasa	MBSA/S9/44933	F	1	*An.coustani*	Flavi/Alphavirus	Negative
5/31/2024	Mombasa	MBSA/S9/44934	F	24	*Cx.univittatus*	Flavi/Alphavirus	Negative
5/31/2024	Mombasa	MBSA/S9/44935	F	1	*Aedeomyia africana*	Flavi/Alphavirus	Negative
5/31/2024	Mombasa	MBSA/S9/44936	F	5	*Cx.univittatus*	Flavi/Alphavirus	Negative
5/31/2024	Mombasa	MBSA/S9/44937	F	6	*Mn.africana*	Flavi/Alphavirus	Negative
5/31/2024	Mombasa	MBSA/S9/44938	F	25	*Mn.africana*	Flavi/Alphavirus	Negative
5/31/2024	Mombasa	MBSA/S9/44939	F	25	*Mn.africana*	Flavi/Alphavirus	Negative
5/31/2024	Mombasa	MBSA/S9/44940	F	25	*Mn.africana*	Flavi/Alphavirus	Negative
5/31/2024	Mombasa	MBSA/S9/44941	F	25	*Mn.africana*	Flavi/Alphavirus	Negative
5/31/2024	Mombasa	MBSA/S9/44942	F	17	*Mn.africana*	Flavi/Alphavirus	Negative
5/31/2024	Mombasa	MBSA/S9/44943	F	1	*Ae.vittatus*	Flavi/Alphavirus	Negative
5/31/2024	Mombasa	MBSA/S9/44944	F	1	*Aedeomyia africana*	Flavi/Alphavirus	Negative
5/31/2024	Mombasa	MBSA/S9/44945	F	3	*Ae.circumluteolus*	Flavi/Alphavirus	Negative
5/31/2024	Mombasa	MBSA/S9/44946	F	4	*An.squamosus*	Flavi/Alphavirus	Negative
5/31/2024	Mombasa	MBSA/S9/44947	F	3	*Cx.annulioris*	Flavi/Alphavirus	Negative
5/31/2024	Mombasa	MBSA/S9/44948	F	19	*Cx.univittatus*	Flavi/Alphavirus	Negative
5/28/2024	Mombasa	MBSA/S4/44949	F	2	*Ae.pembaensis*	Flavi/Alphavirus	Negative
5/28/2024	Mombasa	MBSA/S4/44950	F	1	*Eret.chryogaster*	Flavi/Alphavirus	Negative
5/28/2024	Mombasa	MBSA/S4/44951	F	7	*Cx.pipiens*	Flavi/Alphavirus	Negative
5/28/2024	Mombasa	MBSA/S4/44952	F	4	*Cx.univittatus*	Flavi/Alphavirus	Negative
5/28/2024	Mombasa	MBSA/S4/44953	F	2	*Mn.uniformis*	Flavi/Alphavirus	Negative
5/28/2024	Mombasa	MBSA/S4/44954	F	1	*Ae.aegypti*	Flavi/Alphavirus	Negative
5/28/2024	Mombasa	MBSA/S4/44955	F	1	*Ae.pembaensis*	Flavi/Alphavirus	Negative
5/28/2024	Mombasa	MBSA/S4/44956	F	1	*Cx.pipiens*	Flavi/Alphavirus	Negative
5/28/2024	Mombasa	MBSA/S4/44957	F	1	*Cx.annulioris*	Flavi/Alphavirus	Negative
5/28/2024	Mombasa	MBSA/S4/44958	F	5	*Mn.uniformis*	Flavi/Alphavirus	Negative
5/28/2024	Mombasa	MBSA/S4/44959	F	1	*Ae.vittatus*	Flavi/Alphavirus	Negative
5/28/2024	Mombasa	MBSA/S4/44960	F	1	*Ae.aegypti*	Flavi/Alphavirus	Negative
5/28/2024	Mombasa	MBSA/S4/44961	F	4	*Cx.pipiens*	Flavi/Alphavirus	Negative
5/28/2024	Mombasa	MBSA/S4/44962	F	2	*Ae.pembaensis*	Flavi/Alphavirus	Negative
5/28/2024	Mombasa	MBSA/S4/44963	F	1	*Mn.uniformis*	Flavi/Alphavirus	Negative
5/28/2024	Mombasa	MBSA/S4/44964	F	1	*Ae.vittatus*	Flavi/Alphavirus	Negative
5/28/2024	Mombasa	MBSA/S4/44965	F	1	*Cx.annulioris*	Flavi/Alphavirus	Negative
5/28/2024	Mombasa	MBSA/S4/44966	F	3	*Ae.aegypti*	Flavi/Alphavirus	Negative
5/28/2024	Mombasa	MBSA/S4/44967	F	3	*Ae.vittatus*	Flavi/Alphavirus	Negative
5/28/2024	Mombasa	MBSA/S4/44968	F	7	*Cx.pipiens*	Flavi/Alphavirus	Negative
5/28/2024	Mombasa	MBSA/S4/44969	F	3	*Mn.uniformis*	Flavi/Alphavirus	Negative
10/5/2023	Mombasa	MBSA/S5/44970	F	1	*Cx.cinereus*	Flavi/Alphavirus	Negative
10/5/2023	Mombasa	MBSA/S5/44971	F	3	*Cx.pipiens*	Flavi/Alphavirus	Negative
10/5/2023	Mombasa	MBSA/S5/44972	F	14	*Cx.pipiens*	Flavi/Alphavirus	Negative
10/5/2023	Mombasa	MBSA/S5/44973	F	1	*Ae.aegypti*	Flavi/Alphavirus	Negative
10/5/2023	Mombasa	MBSA/S5/44974	M	1	*Ae.aegypti*	Flavi/Alphavirus	Negative
10/5/2023	Mombasa	MBSA/S5/44975	F	5	*Cx.pipiens*	Flavi/Alphavirus	Negative
10/5/2023	Mombasa	MBSA/S5/44976	F	1	*Cx.pipiens*	Flavi/Alphavirus	Negative
10/5/2023	Mombasa	MBSA/S5/44977	F	1	*Ae.aegypti*	Flavi/Alphavirus	Negative
10/5/2023	Mombasa	MBSA/S5/44978	F	4	*Ae.aegypti*	Flavi/Alphavirus	Negative
10/5/2023	Mombasa	MBSA/S5/44979	F	1	*Cx.pipiens*	Flavi/Alphavirus	Negative
10/5/2023	Mombasa	MBSA/S5/44980	F	3	*Cx.pipiens*	Flavi/Alphavirus	Negative
10/5/2023	Mombasa	MBSA/S5/44981	F	1	*Ae.aegypti*	Flavi/Alphavirus	Negative
10/5/2023	Mombasa	MBSA/S5/44982	M	1	*Ae.aegypti*	Flavi/Alphavirus	Negative

10/5/2023	Mombasa	MBSA/S5/44983	F	2	*Cx.pipiens*	Flavi/Alphavirus	Negative
10/5/2023	Mombasa	MBSA/S5/44984	F	1	*Ae.aegypti*	Flavi/Alphavirus	Negative
10/5/2023	Mombasa	MBSA/S5/44985	F	1	*Cx.pipiens*	Flavi/Alphavirus	Negative
10/5/2023	Mombasa	MBSA/S5/44986	F	4	*Ae.aegypti*	Flavi/Alphavirus	Negative
10/5/2023	Mombasa	MBSA/S5/44987	M	8	*Ae.aegypti*	Flavi/Alphavirus	Negative
10/5/2023	Mombasa	MBSA/S5/44988	F	5	*Ae.aegypti*	Flavi/Alphavirus	Negative
10/5/2023	Mombasa	MBSA/S5/44989	M	10	*Ae.aegypti*	Flavi/Alphavirus	Negative
10/5/2023	Mombasa	MBSA/S5/44990	F	25	*Cx.pipiens*	Flavi/Alphavirus	Negative
10/5/2023	Mombasa	MBSA/S5/44991	F	23	*Cx.pipiens*	Flavi/Alphavirus	Negative
10/5/2023	Mombasa	MBSA/S5/44992	F	2	*Ae.aegypti*	Flavi/Alphavirus	Negative
10/5/2023	Mombasa	MBSA/S5/44993	M	6	*Ae.aegypti*	Flavi/Alphavirus	Negative
10/5/2023	Mombasa	MBSA/S5/44994	F	4	*Cx.pipiens*	Flavi/Alphavirus	Negative
10/5/2023	Mombasa	MBSA/S5/44995	F	1	*Ae.aegypti*	Flavi/Alphavirus	Negative
10/5/2023	Mombasa	MBSA/S5/44996	M	1	*Ae.aegypti*	Flavi/Alphavirus	Negative
10/5/2023	Mombasa	MBSA/S5/44997	F	25	*Cx.pipiens*	Flavi/Alphavirus	Negative
10/5/2023	Mombasa	MBSA/S5/44998	F	10	*Cx.pipiens*	Flavi/Alphavirus	Negative
10/5/2023	Mombasa	MBSA/S5/44999	M	2	*Ae.aegypti*	Flavi/Alphavirus	Negative
10/5/2023	Mombasa	MBSA/S5/45000	F	24	*Cx.pipiens*	Flavi/Alphavirus	Negative
10/6/2023	Mombasa	MBSA/S1/45001	F	5	*Cx.pipiens*	Flavi/Alphavirus	Negative
10/6/2023	Mombasa	MBSA/S1/45002	M	25	*Ae.vittatus*	Flavi/Alphavirus	Negative
10/6/2023	Mombasa	MBSA/S1/45003	M	25	*Ae.vittatus*	Flavi/Alphavirus	Negative
10/6/2023	Mombasa	MBSA/S1/45004	M	10	*Ae.vittatus*	Flavi/Alphavirus	Negative
10/6/2023	Mombasa	MBSA/S1/45005	F	25	*Ae.vittatus*	Flavi/Alphavirus	Negative
10/6/2023	Mombasa	MBSA/S1/45006	F	25	*Ae.vittatus*	Flavi/Alphavirus	Negative
10/6/2023	Mombasa	MBSA/S1/45007	F	25	*Ae.vittatus*	Flavi/Alphavirus	Negative
10/6/2023	Mombasa	MBSA/S1/45008	F	25	*Ae.vittatus*	Flavi/Alphavirus	Negative
10/6/2023	Mombasa	MBSA/S1/45009	F	25	*Ae.vittatus*	Flavi/Alphavirus	Negative
10/6/2023	Mombasa	MBSA/S1/45010	F	1	*Eret.chryogaster*	Flavi/Alphavirus	Negative
10/6/2023	Mombasa	MBSA/S1/45011	F	8	*Cx.pipiens*	Flavi/Alphavirus	Negative
10/6/2023	Mombasa	MBSA/S1/45012	F	4	*Ae.vittatus*	Flavi/Alphavirus	Negative
10/6/2023	Mombasa	MBSA/S1/45013	M	7	*Ae.vittatus*	Flavi/Alphavirus	Negative
10/6/2023	Mombasa	MBSA/S1/45014	F	13	*Ae.aegypti*	Flavi/Alphavirus	Negative
10/6/2023	Mombasa	MBSA/S1/45015	M	3	*Ae.aegypti*	Flavi/Alphavirus	Negative
10/6/2023	Mombasa	MBSA/S1/45016	F	3	*Ae.aegypti*	Flavi/Alphavirus	Negative
10/6/2023	Mombasa	MBSA/S1/45017	F	6	*Cx.pipiens*	Flavi/Alphavirus	Negative
10/6/2023	Mombasa	MBSA/S1/45018	F	4	*Ae.vittatus*	Flavi/Alphavirus	Negative
10/6/2023	Mombasa	MBSA/S1/45019	F	10	*Cx.pipiens*	Flavi/Alphavirus	Negative
10/6/2023	Mombasa	MBSA/S1/45020	F	1	*Ae.aegypti*	Flavi/Alphavirus	Negative
10/6/2023	Mombasa	MBSA/S1/45021	M	6	*Ae.vittatus*	Flavi/Alphavirus	Negative
10/6/2023	Mombasa	MBSA/S1/45022	F	20	*Ae.vittatus*	Flavi/Alphavirus	Negative
10/6/2023	Mombasa	MBSA/S1/45023	F	1	*Cx.pipiens*	Flavi/Alphavirus	Negative
10/6/2023	Mombasa	MBSA/S1/45024	F	3	*Ae.aegypti*	Flavi/Alphavirus	Negative
10/6/2023	Mombasa	MBSA/S1/45025	F	1	*Ae.vittatus*	Flavi/Alphavirus	Negative
10/6/2023	Mombasa	MBSA/S1/45026	M	1	*Ae.vittatus*	Flavi/Alphavirus	Negative
10/6/2023	Mombasa	MBSA/S1/45027	F	3	*Cx.pipiens*	Flavi/Alphavirus	Negative
10/6/2023	Mombasa	MBSA/S1/45028	M	4	*Ae.aegypti*	Flavi/Alphavirus	Negative
10/6/2023	Mombasa	MBSA/S1/45029	F	1	*Ae.aegypti*	Flavi/Alphavirus	Negative
10/6/2023	Mombasa	MBSA/S1/45030	M	3	*Ae.aegypti*	Flavi/Alphavirus	Negative
10/6/2023	Mombasa	MBSA/S1/45031	F	16	*Cx.pipiens*	Flavi/Alphavirus	Negative
10/6/2023	Mombasa	MBSA/S1/45032	F	2	*Ae.aegypti*	Flavi/Alphavirus	Negative

10/6/2023	Mombasa	MBSA/S1/45033	F	9	*Cx.pipiens*	Flavi/Alphavirus	Negative
10/6/2023	Mombasa	MBSA/S1/45034	F	1	*Cx.pipiens*	Flavi/Alphavirus	Negative
10/6/2023	Mombasa	MBSA/S1/45035	F	1	*Ae.aegypti*	Flavi/Alphavirus	Negative
10/6/2023	Mombasa	MBSA/S1/45036	M	8	*Ae.aegypti*	Flavi/Alphavirus	Negative
10/6/2023	Mombasa	MBSA/S1/45037	M	13	*Ae.aegypti*	Flavi/Alphavirus	Negative
10/6/2023	Mombasa	MBSA/S1/45038	F	5	*Ae.aegypti*	Flavi/Alphavirus	Negative
10/6/2023	Mombasa	MBSA/S1/45039	F	21	*Cx.pipiens*	Flavi/Alphavirus	Negative
10/6/2023	Mombasa	MBSA/S1/45040	M	3	*Ae.aegypti*	Flavi/Alphavirus	Negative
10/6/2023	Mombasa	MBSA/S1/45041	F	2	*Ae.aegypti*	Flavi/Alphavirus	Negative
10/6/2023	Mombasa	MBSA/S1/45042	F	25	*Cx.pipiens*	Flavi/Alphavirus	Negative
10/6/2023	Mombasa	MBSA/S1/45043	F	24	*Cx.pipiens*	Flavi/Alphavirus	Negative
10/7/2023	Mombasa	MBSA/S7/45044	F	1	*Cx.pipiens*	Flavi/Alphavirus	Negative
10/7/2023	Mombasa	MBSA/S7/45045	M	1	*Ae.aegypti*	Flavi/Alphavirus	Negative
10/7/2023	Mombasa	MBSA/S7/45046	F	1	*Mn.uniformis*	Flavi/Alphavirus	Negative
10/7/2023	Mombasa	MBSA/S7/45047	F	2	*Ae.vittatus*	Flavi/Alphavirus	Negative
10/7/2023	Mombasa	MBSA/S7/45048	F	3	*Ae.aegypti*	Flavi/Alphavirus	Negative
10/7/2023	Mombasa	MBSA/S7/45049	F	9	*Cx.univittatus*	Flavi/Alphavirus	Negative
10/7/2023	Mombasa	MBSA/S7/45050	F	1	*Eret.chryogaster*	Flavi/Alphavirus	Negative
10/7/2023	Mombasa	MBSA/S7/45051	M	4	*Ae.aegypti*	Flavi/Alphavirus	Negative
10/7/2023	Mombasa	MBSA/S7/45052	F	10	*Ae.aegypti*	Flavi/Alphavirus	Negative
10/7/2023	Mombasa	MBSA/S7/45053	F	3	*Cx.pipiens*	Flavi/Alphavirus	Negative
10/7/2023	Mombasa	MBSA/S7/45054	M	1	*Ae.aegypti*	Flavi/Alphavirus	Negative
10/7/2023	Mombasa	MBSA/S7/45055	F	3	*Ae.aegypti*	Flavi/Alphavirus	Negative
10/7/2023	Mombasa	MBSA/S7/45056	F	5	*Cx.pipiens*	Flavi/Alphavirus	Negative
10/7/2023	Mombasa	MBSA/S7/45057	F	5	*Cx.univittatus*	Flavi/Alphavirus	Negative
10/7/2023	Mombasa	MBSA/S7/45058	F	1	*Ae.aegypti*	Flavi/Alphavirus	Negative
10/7/2023	Mombasa	MBSA/S7/45059	F	1	*Cx.pipiens*	Flavi/Alphavirus	Negative
10/7/2023	Mombasa	MBSA/S7/45060	F	3	*Cx.univittatus*	Flavi/Alphavirus	Negative
10/7/2023	Mombasa	MBSA/S7/45061	F	4	*Ae.aegypti*	Flavi/Alphavirus	Negative
10/7/2023	Mombasa	MBSA/S7/45062	F	2	*Cx.pipiens*	Flavi/Alphavirus	Negative
10/7/2023	Mombasa	MBSA/S7/45063	F	2	*Cx.univittatus*	Flavi/Alphavirus	Negative
10/7/2023	Mombasa	MBSA/S7/45064	F	1	*Ae.aegypti*	Flavi/Alphavirus	Negative
10/7/2023	Mombasa	MBSA/S7/45065	F	2	*Ae.aegypti*	Flavi/Alphavirus	Negative
10/7/2023	Mombasa	MBSA/S7/45066	F	1	*Cx.pipiens*	Flavi/Alphavirus	Negative
10/7/2023	Mombasa	MBSA/S7/45067	F	1	*Eret.chryogaster*	Flavi/Alphavirus	Negative
10/7/2023	Mombasa	MBSA/S7/45068	F	10	*Ae.aegypti*	Flavi/Alphavirus	Negative
10/7/2023	Mombasa	MBSA/S7/45069	M	13	*Ae.aegypti*	Flavi/Alphavirus	Negative
10/7/2023	Mombasa	MBSA/S7/45070	F	2	*Cx.pipiens*	Flavi/Alphavirus	Negative
10/7/2023	Mombasa	MBSA/S7/45071	F	1	*Cx.univittatus*	Flavi/Alphavirus	Negative
10/7/2023	Mombasa	MBSA/S7/45072	F	3	*Ae.aegypti*	Flavi/Alphavirus	Negative
10/7/2023	Mombasa	MBSA/S7/45073	F	25	*Cx.pipiens*	Flavi/Alphavirus	Negative
10/7/2023	Mombasa	MBSA/S7/45074	F	14	*Cx.pipiens*	Flavi/Alphavirus	Negative
10/7/2023	Mombasa	MBSA/S7/45075	F	10	*Ae.aegypti*	Flavi/Alphavirus	Negative
10/7/2023	Mombasa	MBSA/S7/45076	M	13	*Ae.aegypti*	Flavi/Alphavirus	Negative
10/7/2023	Mombasa	MBSA/S7/45077	F	1	*Ae.aegypti*	Flavi/Alphavirus	Negative
10/7/2023	Mombasa	MBSA/S7/45078	F	2	*Cx.pipiens*	Flavi/Alphavirus	Negative
10/7/2023	Mombasa	MBSA/S1/45079	M	5	*Ae.aegypti*	Flavi/Alphavirus	Negative
10/7/2023	Mombasa	MBSA/S1/45080	F	25	*Cx.pipiens*	Flavi/Alphavirus	Negative
10/7/2023	Mombasa	MBSA/S1/45081	F	24	*Cx.pipiens*	Flavi/Alphavirus	Negative
10/7/2023	Mombasa	MBSA/S1/45082	M	2	*Ae.aegypti*	Flavi/Alphavirus	Negative

10/7/2023	Mombasa	MBSA/S1/45083	F	1	*Ae.aegypti*	Flavi/Alphavirus	Negative
10/7/2023	Mombasa	MBSA/S1/45084	F	25	*Cx.pipiens*	Flavi/Alphavirus	Negative
10/7/2023	Mombasa	MBSA/S1/45085	F	20	*Cx.pipiens*	Flavi/Alphavirus	Negative
10/7/2023	Mombasa	MBSA/S1/45086	M	5	*Ae.aegypti*	Flavi/Alphavirus	Negative
10/7/2023	Mombasa	MBSA/S1/45087	F	1	*Ae.aegypti*	Flavi/Alphavirus	Negative
10/7/2023	Mombasa	MBSA/S1/45088	F	4	*Cx.pipiens*	Flavi/Alphavirus	Negative
10/7/2023	Mombasa	MBSA/S1/45089	M	1	*Ae.aegypti*	Flavi/Alphavirus	Negative
10/8/2023	Mombasa	MBSA/S1/45090	M	3	*Ae.aegypti*	Flavi/Alphavirus	Negative
10/8/2023	Mombasa	MBSA/S1/45091	F	9	*Cx.pipiens*	Flavi/Alphavirus	Negative
10/8/2023	Mombasa	MBSA/S1/45092	M	1	*Ae.aegypti*	Flavi/Alphavirus	Negative
10/8/2023	Mombasa	MBSA/S1/45093	F	1	*Ae.aegypti*	Flavi/Alphavirus	Negative
10/8/2023	Mombasa	MBSA/S1/45094	F	2	*Cx.pipiens*	Flavi/Alphavirus	Negative
10/8/2023	Mombasa	MBSA/S1/45095	F	10	*Cx.pipiens*	Flavi/Alphavirus	Negative
10/8/2023	Mombasa	MBSA/S1/45096	F	3	*Cx.pipiens*	Flavi/Alphavirus	Negative
10/8/2023	Mombasa	MBSA/S1/45097	F	3	*Cx.pipiens*	Flavi/Alphavirus	Negative
10/8/2023	Mombasa	MBSA/S1/45098	M	16	*Ae.aegypti*	Flavi/Alphavirus	Negative
10/8/2023	Mombasa	MBSA/S1/45099	F	25	*Ae.aegypti*	Flavi/Alphavirus	Negative
10/8/2023	Mombasa	MBSA/S1/45100	F	7	*Cx.pipiens*	Flavi/Alphavirus	Negative
10/8/2023	Mombasa	MBSA/S1/45101	M	5	*Ae.aegypti*	Flavi/Alphavirus	Negative
10/8/2023	Mombasa	MBSA/S1/45102	F	20	*Ae.aegypti*	Flavi/Alphavirus	Negative
10/8/2023	Mombasa	MBSA/S1/45103	M	2	*Ae.vittatus*	Flavi/Alphavirus	Negative
10/8/2023	Mombasa	MBSA/S1/45104	F	25	*Ae.vittatus*	Flavi/Alphavirus	Negative
10/8/2023	Mombasa	MBSA/S1/45105	F	20	*Ae.vittatus*	Flavi/Alphavirus	Negative
10/8/2023	Mombasa	MBSA/S1/45106	F	7	*Ae.aegypti*	Flavi/Alphavirus	Negative
10/8/2023	Mombasa	MBSA/S1/45107	M	15	*Ae.aegypti*	Flavi/Alphavirus	Negative
10/8/2023	Mombasa	MBSA/S1/45108	F	4	*Cx.pipiens*	Flavi/Alphavirus	Negative
10/8/2023	Mombasa	MBSA/S1/45109	M	2	*Ae.vittatus*	Flavi/Alphavirus	Negative
10/8/2023	Mombasa	MBSA/S1/45110	F	9	*Ae.vittatus*	Flavi/Alphavirus	Negative
10/8/2023	Mombasa	MBSA/S1/45111	M	18	*Ae.aegypti*	Flavi/Alphavirus	Negative
10/8/2023	Mombasa	MBSA/S1/45112	F	25	*Ae.aegypti*	Flavi/Alphavirus	Negative
10/8/2023	Mombasa	MBSA/S1/45113	F	1	*Eret.chryogaster*	Flavi/Alphavirus	Negative
10/8/2023	Mombasa	MBSA/S1/45114	M	23	*Ae.aegypti*	Flavi/Alphavirus	Negative
10/8/2023	Mombasa	MBSA/S1/45115	F	25	*Ae.aegypti*	Flavi/Alphavirus	Negative
10/8/2023	Mombasa	MBSA/S1/45116	M	18	*Ae.vittatus*	Flavi/Alphavirus	Negative
10/8/2023	Mombasa	MBSA/S1/45117	F	25	*Ae.vittatus*	Flavi/Alphavirus	Negative
10/8/2023	Mombasa	MBSA/S1/45118	F	18	*Ae.vittatus*	Flavi/Alphavirus	Negative
10/8/2023	Mombasa	MBSA/S1/45119	F	7	*Cx.pipiens*	Flavi/Alphavirus	Negative
10/8/2023	Mombasa	MBSA/S1/45120	F	25	*Ae.vittatus*	Flavi/Alphavirus	Negative
10/8/2023	Mombasa	MBSA/S1/45121	F	25	*Ae.vittatus*	Flavi/Alphavirus	Negative
10/8/2023	Mombasa	MBSA/S1/45122	F	25	*Ae.vittatus*	Flavi/Alphavirus	Negative
10/8/2023	Mombasa	MBSA/S1/45123	F	14	*Ae.vittatus*	Flavi/Alphavirus	Negative
10/8/2023	Mombasa	MBSA/S1/45124	M	25	*Ae.vittatus*	Flavi/Alphavirus	Negative
10/8/2023	Mombasa	MBSA/S1/45125	M	14	*Ae.vittatus*	Flavi/Alphavirus	Negative
10/8/2023	Mombasa	MBSA/S1/45126	F	2	*Cx.pipiens*	Flavi/Alphavirus	Negative
10/8/2023	Mombasa	MBSA/S1/45127	F	7	*Ae.aegypti*	Flavi/Alphavirus	Negative
10/8/2023	Mombasa	MBSA/S1/45128	M	6	*Ae.aegypti*	Flavi/Alphavirus	Negative
10/8/2023	Mombasa	MBSA/S1/45129	F	13	*Ae.vittatus*	Flavi/Alphavirus	Negative
